# *Helicobacter pylori* CagA Protein Regulating the Biological Characteristics of Gastric Cancer through the *miR-155-5p*/SMAD2/SP1 axis

**DOI:** 10.3390/pathogens11080846

**Published:** 2022-07-28

**Authors:** Leilei Wu, Fei Jiang, Xiaobing Shen

**Affiliations:** 1 Education Ministry Key Laboratory for Environmental Medicine Engineering, Department of Occupation and Environment Health, School of Public Health, Southeast University, Nanjing 210009, China; 15735178726@163.com (L.W.); fei.jiang@seu.edu.cn (F.J.); 2 Department of Epidemiology and Health Statistics, School of Public Health, Southeast University, Nanjing 210009, China

**Keywords:** gastric cancer, CagA, *miR-155-5p*, SMAD2, SP1

## Abstract

*Helicobacter pylori* (*Hp*) is a grade Ι carcinogen of gastric cancer (GC), and its high infection rate seriously affects human health. Cytotoxin-associated gene A (CagA) plays a key role in the carcinogenesis of *Hp* as one of its main virulence factors. *miR-155-5p* is abnormally expressed in patients with GC, associated with the occurrence and development of cancer. However, little is known about the association between CagA and *miR-155-5p*. (1) Background: This study explored the association and mechanism of CagA and *miR-155-5p* in GC. (2) Methods: The *CagA* sequence was obtained from the NCBI. After sequence optimization, it was connected to the pcDNA3.1 vector to construct a CagA eukaryotic expression plasmid (pcDNA-*CagA*). Quantitative real-time polymerase chain reaction (qRT-PCR) was used to investigate the expression of *miR-155-5p* and *CagA* in GC cells. The function of CagA on GC cells was detected by CCK8, wound healing, and Transwell assays. Similarly, the function of *miR-155-5p* was also studied through the above functional experiments after the *miR-155-5p* overexpression and knockdown models had successfully been constructed. The associations among CagA, *miR-155-5p*, and SMAD2/SP1 were evaluated using RNA immunoprecipitation (RIP) and rescue experiments. (3) Results: The expression of *miR-155-5p* was significantly reduced in GC cells, and the expression of *miR-155-5p* was further reduced after CagA induction. Both overexpressed CagA and knockdown *miR-155-5p* cell models enhanced malignant transformation, whereas overexpressed *miR-155-5p* inhibited malignant transformation in vitro. The function of *miR-155-5p* on GC cells could be influenced by CagA. We also found that the influence of *miR-155-5p* on SMAD2 and SP1 could be regulated by CagA. (4) Conclusions: CagA potentially regulates the biological function of GC cells through the *miR-155-5p*/SMAD2/SP1 axis. *miR-155-5p* could be a therapeutic target for GC related to CagA.

## 1. Introduction

*Helicobacter pylori* (*Hp*) is a Gram-negative micro-aerobic spiral bacterium, which can colonize the human body for a long time, promote the inflammatory reaction of gastric mucosa, trigger further progression, and lead to cancer. It is a grade I carcinogen [1]. The global infection rate of *Hp* is about 50% [2,3], 10–15% of infected people develop ulcers, and nearly 1% develop gastric adenocarcinoma [4]. However, about 90% of noncardiac gastric cancer (GC) in the world is caused by *Hp* [5], and the infection rate of *Hp* in China is as high as 80% [6]. It has been confirmed that eradicating *Hp* is related to reducing the incidence rate of GC. A study in Taiwan found that the average incidence of GC decreased from 40.3/100,000 person years to 30.4/100,000 person years after eradicating *Hp*, indicating that eradicating *Hp* could help prevent the occurrence of GC [7].

*Hp* has a variety of virulence factors, and each of them plays different role in the carcinogenesis of *Hp*. As one of the main virulence factors of *Hp*, cytotoxin-associated gene A (CagA) plays a key role in the occurrence of GC caused by *Hp* infection. It was found that infection with *CagA*-positive *Hp* is related to the severity and poor clinical outcome of patients with GC [8], which was also considered to be a bacterial oncoprotein. Therefore, inactivating CagA may be an effective method for the treatment of GC [8,9].

miRNA, as a small endogenous functional molecule with 19–22 nucleotides, can target mRNA molecules, regulate their expression by degrading the target mRNA or inhibiting their translation level, and participate in a variety of biological processes in all stages of cancer, such as cell proliferation, autophagy, and apoptosis [10,11]. *miR-155-5p* is downregulated in GC [12,13], and the degree of downregulation was related to the grading and metastasis of advanced gastric tumors [13]. In addition, Zhu et al. found that *miR-155-5p* inhibition promotes the transformation of bone marrow mesenchymal stem cells into GC-tissue-derived MSC-like cells via nuclear factor kappa B p65 activation [14]. As a tumor suppressor, it plays an important role in the development of GC [15]. However, the carcinogenic mechanism of *miR-155-5p* in GC is still unclear.

CagA plays an important role in the progression of GC, and *miR-155-5p* is also involved in the development of GC. Therefore, our study raises the question: can the carcinogenic impact of *miR-155-5p* can be regulated by CagA? In order to prove the association between CagA and *miR-155-5p*, we constructed a CagA eukaryotic expression plasmid and designed functional experiments, further exploring the potential mechanism between *miR-155-5p* and CagA. In conclusion, our study aimed to explore the potential mechanism of CagA and provide a theoretical basis for the targeted treatment of CagA-related GC.

## 2. Materials and Methods

### 2.1. Construction of CagA Eukaryotic Expression Plasmid

The eukaryotic expression plasmid pcDNA3.1 (+) used in the experiment was purchased from the Jie Rui Biological Company (Shanghai, China). Firstly, the empty plasmid vector and the full-length *CagA* gene were digested with EcoRI-XhoI. Secondly, the treated vector and gene were recovered, and the full-length *CagA* gene/EcoRI-XhoI 4 μL, pcDNA3.1/EcoRI-XhoI 2 μL, T4 DNA ligase 1 μL, 10 × T4 buffer 2 μL, and 11 μL water were taken to configure a 20 μL ligation reaction system, incuvate them at 22 °C for 30 min, and preliminarily synthesize the CagA eukaryotic expression plasmid. Then, this was transformed into XL10-Gold competent cells, and the plasmid was identified by colony screening and Sanger sequencing; this is shown in File S1 and File S2. After the plasmid construction was proven to be successful, the plasmid was extracted using the plasmid small amount extraction kit GK2004 and the GoldHi EndoFree Plasmid Midi Kit CW2581S for subsequent experiments.

### 2.2. Cell Culture and Transfection

The normal gastric mucosal epithelial cells GES-1 and GC cells HGC-27 were purchased from Saiku Biological Company (Guangzhou, China) and human gastric adenocarcinoma cells AGS from Wobixin Biological Company (Nanjing, China). All cells were cultured in RPMI 1640 (Gibico, Waltham, MA, USA), containing 10% FBS (BI, Kibbutz Beit Haemek, Israel), with100 IU/mL penicillin and 100 IU/mL streptomycin (Gibico, Waltham, MA, USA), incubated at 37 °C with 5% CO_2_.

The sequences of *miR-155-5p* mimics, *miR-155-5p* mimics NC (normal control), *miR-155-5p* inhibitors, and *miR-155-5p* inhibitors NC are shown in Table S1. Cells were plated into 6-well, 24-well, or 96-well plates and transfected using Lipofectamine 2000 (Invitrogen, Carlsbad, CA, USA), according to the manufacturer’s instructions.

### 2.3. Quantitative Real-Time Polymerase Chain Reaction (qRT-PCR) 

Total RNA was isolated from cell lines with TRIGene reagent (Genestar, Beijing, China) by strictly following the manufacturer’s instructions. RNA was reverse-transcribed into cDNA according to the protocol of the PrimeScript RT Regent Kit (TaKaRa, Tokyo, Japan) and miRNA First Strand cDNA Synthesis (Tailing Reaction) (Sangon, Shanghai, China), respectively. qRT-PCR was used to detect the expression levels of *CagA*, *miR-155-5p*, *SMAD2* and *SP1* using the SYBR Green Master Mixture (Yisheng, Shanghai, China) reagent in QuantStudio 7 (ThermoFisher, Waltham, MA, USA). The *CagA* and mRNA levels were normalized with GAPDH. The miRNA levels were normalized with U6. The qRT-PCR primer sequences were synthesized by the JieRui Company (Shanghai, China) or Sangon Company (Shanghai, China), and are listed in Table S2. The 2^−ΔΔCt^ method was used to calculate the relative expression.

### 2.4. Western Blot

Cells were lysed in RIPA buffer (Beyotime Biotechnology, Shanghai, China). A BCA assay was used to detect the protein concentration. Total protein samples were separated by SDS-PAGE polyacrylamide gel and transferred onto a PVDF membrane (Millipore, Billerica, MA, USA). The membranes were incubated overnight at 4 °C with primary antibodies. Their concentrations are as follows: CagA (1:500; Santa Cruz, Santa Cruz Biotechnology (Shanghai) Co., Ltd, Shanghai, China); SMAD2 (1:1000, ABclonal, Wuhan, China); SP1 (1:1000, ABclonal, Wuhan, China); GAPDH (1:1000; ABclonal, Wuhan, China). PVDF membranes were washed with TBST 3 times, then incubated with secondary antibody (HRP-conjugated goat/rabbit IgG (1:2000; Transgene, Strasbourg, France) for 1 h at room temperature. Lastly, the protein bands were detected after washing with TBST 5 times. Protein expression was normalized with GAPDH levels. Image J (Version 1.53, National Institutes of Health, Bethesda, MD, USA) was used to quantify the density of each band.

### 2.5. RNA Immunoprecipitation (RIP) 

The RIP assay was carried out using an Immunoprecipitation Kit (Millipore, Billerica, MA, USA), according to the manufacturer’s instructions. AGS cells were harvested and lysed in RIP lysis buffer with a ribonuclease inhibitor and protease inhibitor cocktail. Then, magnetic beads were configured for immunoprecipitation, and protein antibodies SMAD2/SP1/IgG were added for a further 30 min incubation at room temperature. Then, anti-SMAD2/SP1 or anti-IgG magnetic bead antibodies were added to the supernatant of the AGS cell lysate after centrifugation, which were incubated overnight at 4 °C. After the beads were washed with wash buffer, immunocomplexes of SMAD2/SP1/IgG and RNAs were de-crosslinked with protease K buffer at 55 °C for 30 min. The immunoprecipitated RNAs were then purified using TRIzol and ethanol precipitation and subjected to qRT-PCR analysis. 

### 2.6. CCK8 Assay 

The CCK8 assay was performed to measure the proliferative capacity of the GC cells. After cells were seeded in a 96-well plate, we transfected AGS cells with pcDNA-*CagA*, pcDNA-NC (pcDNA3.1, empty vector), *miR-155-5p* mimics, *miR-155-5p* mimics NC, *miR-155-5p* inhibitors, or *miR-155-5p* inhibitors NC. The original medium was removed from each well and they were treated with 90 μL new medium and 10 μL CCK-8 regents after 10, 22, 46 and 70 h incubation at 37 °C with 5% CO_2_, followed by incubation at 37 °C for 2 h. The results were measured at a 450 nm OD value using a microplate reader (Bio-Rad, Hercules, CA, USA). All experiments were performed in triplicate.

### 2.7. Wound Healing Assay 

AGS cells were seeded in 6-well plates. Cell layers in serum-free medium were scratched with a sterile 20 μL pipette tip. After scratching, the debris was removed with PBS. After 48 h incubation, 3 fields were randomly visualized to assess migration. The total wound area was analyzed using ImageJ (Version 1.53, National Institutes of Health, USA) software to assess the cell migration capacity. All experiments had 3 parallel sets.

### 2.8. Transwell Assays 

We assessed cell migrative and invasive abilities. We filled the lower chamber with 500 μL of medium containing 10% FBS, and GC AGS cells were seeded on Matrigel-coated Transwell with 500 μL of serum-free medium. After incubation for 24 h, the cells were fixed with methanol for 15 min, and stained with crystal violet for 10 min. Finally, we took photographs of the cells for quantification. Similarly, the migration procedure of the GC cells was the same as that of invasion experiment, but there was no matrix gel.

### 2.9. Statistical Analysis 

Data analysis was performed using SPSS 22.0, and the results are presented as mean ± standard deviation; data were analyzed with two independent sample t-tests or one-way ANOVA, and further pairwise comparisons were performed using the least significant difference (LSD) method. The significant difference was *p* < 0.05.

## 3. Results

### 3.1. CagA Promoted the Proliferation, Migration, and Invasion of GC Cells 

**Figure 1:**
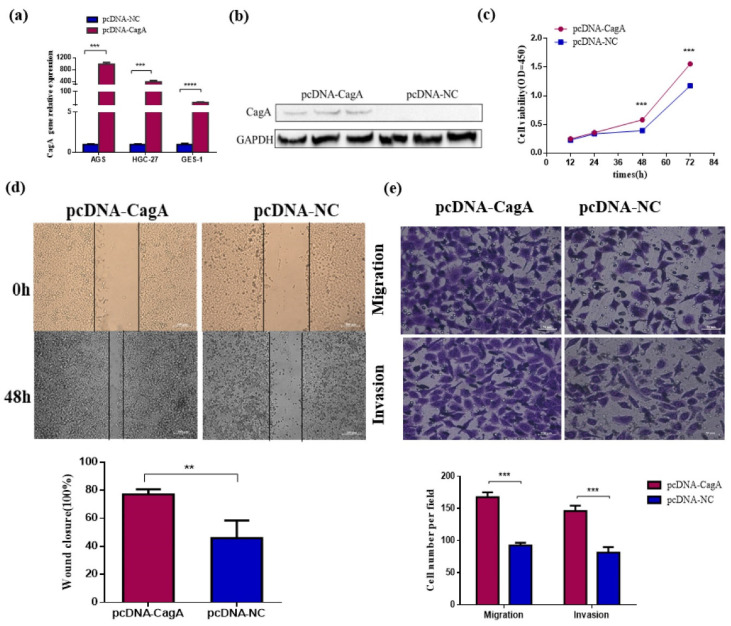
The expression of CagA in AGS cells and the effects of CagA overexpression on the biological characteristics of GC AGS cells. (**a**) qRT-PCR analysis of CagA mRNA expression after treatment with pcDNA-*CagA* and pcDNA-NC. (**b**) Western blot analysis of CagA. (**c**) Assessment of the proliferation of AGS cells transfected with pcDNA-*CagA* and pcDNA-NC by CCK-8 assay. (**d**) Wound healing assay to detect the effect of CagA on cell migration. (**e**) CagA overexpression regulated the migration and invasion of AGS cells. ** *p* < 0.01; *** *p* < 0.001; **** *p* < 0.0001.

We transfected AGS, HGC-27 and GES-1 with pcDNA-*CagA* and pcDNA-NC. Then, detected the expression of *CagA* by qRT-PCR. It was found that *CagA* was overexpressed nearly 1000-fold compared with the pcDNA-NC control group (*p* < 0.001, Figure 1a). AGS cells were used in subsequent experiments for CagA, having the highest transfection efficiency. The results of the Western blot experiment also showed that CagA protein was successfully expressed in AGS (Figure 1b). 

Therefore, we further explored the function of CagA overexpression on GC AGS cells. A CCK-8 assay, wound healing assay, and Transwell assay were used to detect the effects of CagA on cell proliferation, migration, and invasion. The CCK8 assay results showed that CagA overexpression significantly increased cell viability at 48 h and 72 h as compared with NC (*p* < 0.001, Figure 1c). According to the results of wound healing experiments, the overexpression of CagA increased cell migration, (*p* < 0.01, Figure 1d). Similarly, the overexpression of CagA significantly increased the invasion ability of AGS (Figure 1e).

### 3.2. miR-155-Inhibited the Proliferation, Migration, and Invasion of GC Cells

**Figure 2:**
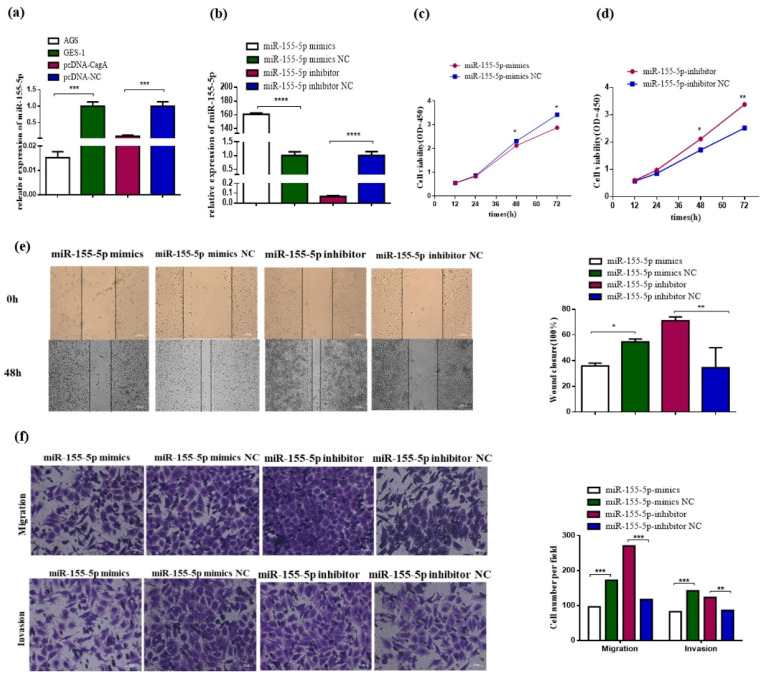
The expression of *miR-155-5p* in AGS cells and the effects of overexpression and knockdown of *miR-155-5p* on the biological characteristics of AGS. (**a**,**b**) qRT-PCR analysis of *miR-155-5p* mRNA expression after treatment with pcDNA-*CagA*, *miR-155-5p* mimics and inhibitor regents. (**c**,**d**) Assessment of the proliferation of AGS cells transfected with *miR-155-5p* mimics and inhibitors by CCK-8 assay. (**e**) Wound healing assay to detect the effect of *miR-155-5p* on cell migration. (**f**) *miR-155-5p* overexpression and knockdown regulated the migration and invasion of AGS cells. * *p* < 0.05; ** *p* < 0.01; *** *p* < 0.001; **** *p* < 0.0001.

Compared with GES-1, *miR-155-5p* was significantly downregulated in AGS, and the overexpression of CagA further downregulated the expression of *miR-155-5p* compared with NC (*p* < 0.001, Figure 2a), which indicated that *miR-155-5p* may be involved in the carcinogenic process of CagA. 

Then, we constructed *miR-155-5p* overexpression and knockdown models (*p* < 0.001, Figure 2b). Gain- and loss-of-function experiments were used to evaluate the effects of *miR-155-5p* on AGS cells. The results indicated that *miR-155-5p* overexpression decreased cell viability (*p* < 0.05, Figure 2c); in contrast, *miR-155-5p* depletion significantly increased cell viability at 48 h and 72 h compared with the respective NC (*p* < 0.05, Figure 2d). According to the results of the wound healing experiments, the overexpression of *miR-155-5p* in AGS cells reduced migration, whereas knocking down *miR-155-5p* in AGS cells increased migration (*p* < 0.05, Figure 2e). The results of the Transwell migration experiment were consistent with the wound healing experiment; similarly, the overexpression of *miR-155-5p* significantly reduced the invasion ability in AGS cells, whereas downregulating *miR-155-5p* in AGS cells significantly increased the invasion ability (Figure 2f). 

### 3.3. CagA Promoted the Proliferation, Migration, and Invasion of GC Cells by Regulating miR-155-5p

**Figure 3:**
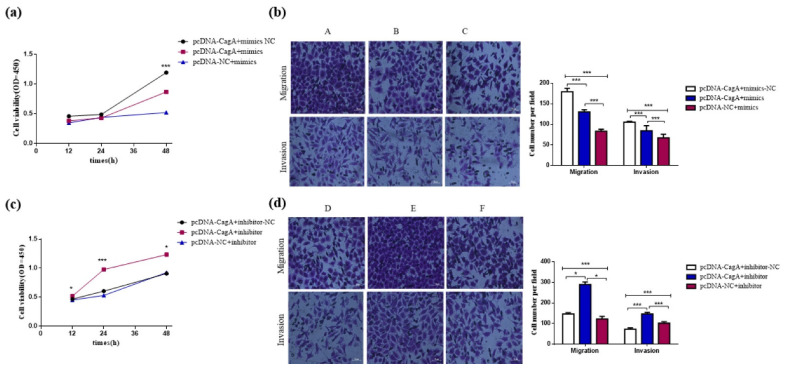
CagA regulates malignant transformation through *miR-155-5p* in vitro. (**a**,**b**) Co-transfection of *miR-155-5p* mimics and pcDNA-*CagA* to investigate malignant transformation of cells with CCK-8 and Transwell assays in the AGS cell line. (**c**,**d**) Co-transfection of *miR-155-5p* inhibitor and pcDNA-*CagA* to investigate malignant transformation of cells with CCK-8 and Transwell assays in the AGS cell line A: pcDNA-*CagA* + *miR-155-5p* mimics NC; B: pcDNA-*CagA* + *miR-155-5p* mimics; C: pcDNA-NC + *miR-155-5p* mimics; D: pcDNA-*CagA* + *miR-155-5p* inhibitors NC; E: pcDNA-*CagA* + *miR-155-5p* inhibitors; F: pcDNA-NC + *miR-155-5p* inhibitors; * *p* < 0.05; *** *p* < 0.001.

We designed rescue experiments to explore whether the role of *miR-155-5p* on AGS could be regulated by CagA. pcDNA-*CagA* + *miR-155-5p* mimics/inhibitors NC, pcDNA-*CagA* + *miR-155-5p* mimics/inhibitors and pcDNA-NC + *miR-155-5p* mimics/inhibitors were co-transfected into GC AGS cells. The results showed that the proliferation, migration, and invasion abilities of the pcDNA-*CagA* + *miR-155-5p* mimics NC group was significantly higher than those of the pcDNA-*CagA* + *miR-155-5p* mimics group, and these abilities of the pcDNA-*CagA* + *miR-155-5p* mimics group were significantly higher than those of the pcDNA-NC + *miR-155-5p* mimics group (Figure 3a,b). When *miR-155-5p* inhibitor was used, the opposite result was produced (Figure 3c,d). These results indicate that the function of *miR-155-5p* on GC cells could be influenced by CagA, and the carcinogenic effects of CagA could be assessed using *miR-155-5p*.

### 3.4. SP1 and SMAD2 Were Targets of miR-155-5p

**Figure 4:**
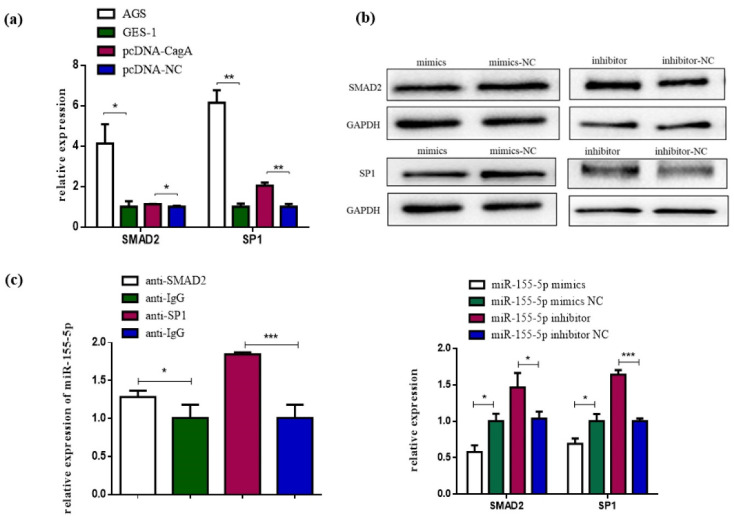
SMAD2 and SP1 were targets of *miR-155-5p*. (**a**) qRT-PCR of *SMAD2* and *SP1* expression in AGS cells transfected with *miR-155-5p* mimics and inhibitor. (**b**) RIP assays with anti-SMAD2, anti-SP1, and anti-IgG antibodies in AGS cell lines were used to detect *miR-155-5p* mRNA levels. (**c**) Western blot analysis of SMAD2 and SP1 transfected with *miR-155-5p* mimics and inhibitor. * *p* < 0.05; ** *p* < 0.01; *** *p* < 0.001.

The downstream target genes of *miR-155-5p* were predicted by Targetscan 8.0 [16] (https://www.targetscan.org/vert_80/) (accessed on 1 October 2021), miRDB 6.0 [17] (http://mirdb.org/index.html) (accessed on 1 October 2021). and miRTarbase 9.0 [18](https://mirtarbase.cuhk.edu.cn/) (accessed on 1 October 2021). RAC1, MITF, EGFR, FOS, ETS1, SMAD2, and SP1 were selected for further investigation based on the literature. Further analysis showed that *SMAD2* and *SP1* were upregulated in AGS cells compared with GES-1 cells, which is in contrast to the expression of *miR-155-5p*, and only the expression levels of *SMAD2* and *SP1* were decreased after *miR-155-5p* overexpression and increased after *miR-155-5p* knockdown (Figure 4a). Therefore, SMAD2 and SP1 were selected for the next Western blot and RIP experiments.

Using Western blots, we found that the expression levels of SMAD2 and SP1 protein in the *miR-155-5p* mimics group decreased compared with the *miR-155-5p* mimics NC group; the expression levels of SMAD2 and SP1 protein in the *miR-155-5p* inhibitors group were increased compared with the *miR-155-5p* inhibitors NC group (Figure 4b).

Then, SMAD2/SP1 antibodies were used to perform RIP experiments in order to analyze whether *miR-155-5p* could interact with SMAD2/SP1 directly. Results showed that the expression levels of *miR-155-5p* in the SMAD2 and SP1 immunoprecipitation complex were 1.27 and 1.85 times higher than negative control group (anti-IgG), respectively (Figure 4c), which proved that *miR-155-5p* could directly bind to SMAD2/SP1. Taken together, our results have demonstrated that *miR-155-5p* regulates SMAD2 and SP1 expression at both the post-transcriptional and protein levels.

### 3.5. CagA Promotes the Proliferation, Migration, and Invasion of GC Cells through the miR-155-5p-SP1/SMAD2 axis

**Figure 5:**
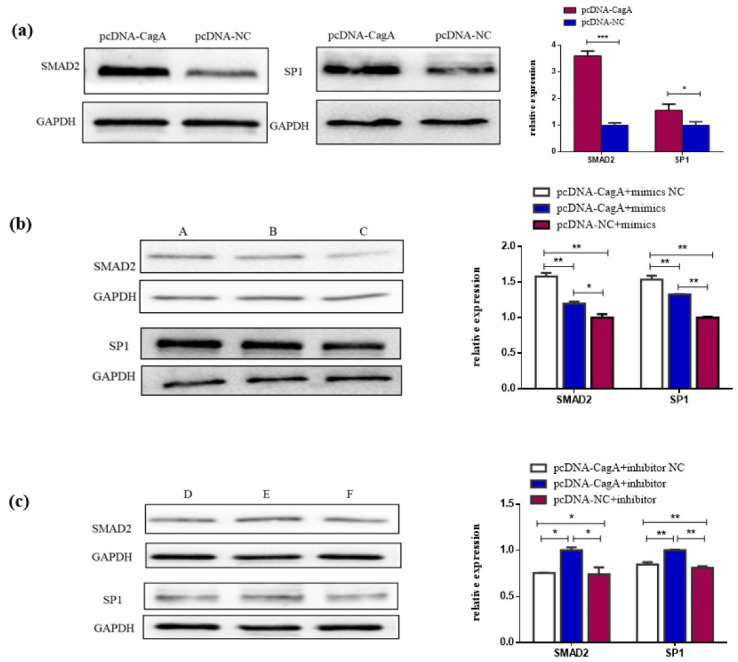
CagA regulates malignant transformation through the *miR-155-5p*/SMAD2/SP1 axis in vitro. (**a**) Western blot analysis of SMAD2 and SP1 transfected with pcDNA-*CagA*. (**b**) Co-transfection of *miR-155-5p* mimics and pcDNA-*CagA* to investigate the level of SMAD2 and SP1 expression with Western blots in the AGS cell line. (**c**)**.** Co-transfection of *miR-155-5p* inhibitors and pcDNA-*CagA* to investigate the levels of SMAD2 and SP1 expression with Western blots in the AGS cell line A: pcDNA-*CagA* + *miR-155-5p* mimics NC; B: pcDNA-*CagA* + *miR-155-5p* mimics; C: pcDNA-NC + *miR-155-5p* mimics; D: pcDNA-*CagA* + *miR-155-5p* inhibitors NC; E: pcDNA-*CagA* + *miR-155-5p* inhibitors; F: pcDNA-NC + *miR-155-5p* inhibitor; * *p* < 0.05; ** *p* < 0.01; *** *p* < 0.001.

Our study found that the overexpression of CagA could increase the expression of SMAD2 and SP1 protein (Figure 5a). Thus, we designed a rescue experiment to explore whether the effects of *miR-155-5p* on SMAD2 and SP1 were regulated by CagA. A Western blot showed that the expression levels of SMAD2 and SP1 protein in the pcDNA-*CagA* + *miR-155-5p* mimics group were significantly decreased compared with the pcDNA-*CagA* + *miR-155-5p* mimics NC group (Figure 5b); the expression levels of SMAD2 and SP1 protein in the pcDNA-*CagA* + *miR-155-5p* mimics group were significantly higher compared with the pcDNA-NC + *miR-155-5p* mimics group (Figure 5b). However, when the inhibitor was used, the results were the opposite

## 4. Discussion

In the process of exploring the carcinogenic ability of *Hp*, have researchers focused their attention on the virulence gene of *Hp*, and they agreed that the *CagA* virulence gene plays an important role in the carcinogenic ability of *Hp* [19,20,21,22]. miRNA participates in the occurrence and development of GC through interaction with oncogenes, tumor suppressor genes, and other cancer-related genes [23,24].

Our research focused on the relationship between CagA and miRNA and explored the mechanism of carcinogenic effects of CagA through miRNA so as to provide some new ideas for the treatment of patients infected with *CagA*-positive *Hp*. The results showed that CagA can promote the cell proliferation, migration, and invasion of GC AGS cells, which indicated that CagA could promote the malignant phenotype of AGS cells. Previous research reported that *CagA*-positive *Hp* infection was associated with a worse disease stage and clinical outcome [19,20,21,25], and our study demonstrates the carcinogenic ability of CagA at the cellular level, which was consistent with previous studies. 

Then, we found the expression of *miR-155-5p* was downregulated in GC AGS cells and further downregulated under the action of CagA, which means that *miR-155-5p* may be a key molecule in the carcinogenic pathway of CagA. Thus, we next verified the function of *miR-155-5p.* After the overexpression and knockdown of *miR-155-5p*, it was found that the overexpression of *miR-155-5p* inhibited the proliferation, migration, and invasion of GC AGS cells, whereas the knockdown of *miR-155-5p* enhanced the proliferation, migration and invasion of cells, indicating that *miR-155-5p* may play an important role in promoting cancer progression as an important functional molecule. However, the expression of *miR-155-5p* is tissue-specific, upregulated in kidney cancer, bladder cancer, and cervical cancer [26,27], and downregulated in liver cancer, atherosclerosis, and GC [12,13,15,28,29], which is consistent with our study, suggesting that *miR-155-5p* is a molecule which warrants further exploration.

Yang et al. found that CagA could upregulate the expression of *miR-223-3p* and participate in the progression of GC by reducing the expression of ARID1A [30]. In this study, we found that CagA could cause \further downregulation of *miR-155-5p* in AGS cells. We also verified the functional role of *miR-155-5p* on AGS. In order to prove whether CagA plays a carcinogenic role through *miR-155-5p*, rescue experiments were designed. Results showed that the overexpression of *miR-155-5p* could inhibit the proliferation, migration, and invasion of AGS cells, which could be restored by the overexpression of CagA; similarly, the knockdown of *miR-155-5p* could enhance the proliferation, migration, and invasion of AGS cells, which could be enhanced by the overexpression of CagA, which indicates that the function of *miR-155-5p* on AGS cells was regulated by CagA. Therefore, we speculate that cells being affected by CagA further lead to the downregulation of *miR-155-5p*, which may promote the occurrence and development of cancer.

The abnormal expression of miRNA could further cause the abnormal expression of downstream target mRNA molecules and promote cancer [31]. In this study, SMAD2 and SP1 were identified as two targets of *miR-155-5p* by qRT-PCR, RIP, and Western blot experiments. SMAD2 could be activated by the TGF-β1 signaling pathway, which, in turn, promotes KLF8 accumulation, leading to cancer progression in breast cancer [32]. In GC, it was found that the downregulation of the phosphorylation level of SMAD2 significantly inhibited the proliferation and migration ability of GC cells [33]. In addition, Niu et al. found that SMAD2 was a target of *miR-155-5p* and played an oncogenic role in the progression of cancer [34], which also supports the results of our study and proves its carcinogenic effects. SP1 was highly expressed in GC, and its activation may serve as a biomarker for poor prognosis and contribute to the development of GC [35]. Lei et al. found that transcription factor SP1 could activate PCAT19, promoting GC progression and triggering poor prognosis [36]. It could also activate p62 to decrease autophagy flux and promote cell proliferation in GC cells [37]. In addition, a meta-analysis related to SP1 showed that SP1 expression was significantly higher in GC tissues than in normal gastric mucosa, and was positively correlated with the invasion depth and TNM stage of GC [38]. The above studies showed that SMAD2 and SP1 could induce carcinogenic effects in GC.

In our study, we verified the association of CagA with SMAD2 and SP1 proteins at the protein level via Western blot and proved that *miR-155-5p* can directly bind SMAD2 and SP1 via an RIP assay. The results showed that the overexpression of *miR-155-5p* caused the downregulation of SMAD2 protein and SP1 protein levels, which could be partially reverted after the overexpression of CagA. Additionally, the knockdown of *miR-155-5p* caused the upregulation of expression levels of SMAD2 and SP1 protein, which were enhanced after the overexpression of CagA. These results indicated that CagA could cause the downregulation of *miR-155-5p* and further upregulation of SMAD2 and SP1 expression, thereby exerting its effect on the proliferation, migration, and invasion ability of GC cells. It is well illustrated that CagA can exert its effects on the proliferation, migration, and invasion abilities of GC cells through the CagA/*miR-155-5p*/SMAD2/SP1 axis. 

## 5. Conclusions

**Scheme 1:**
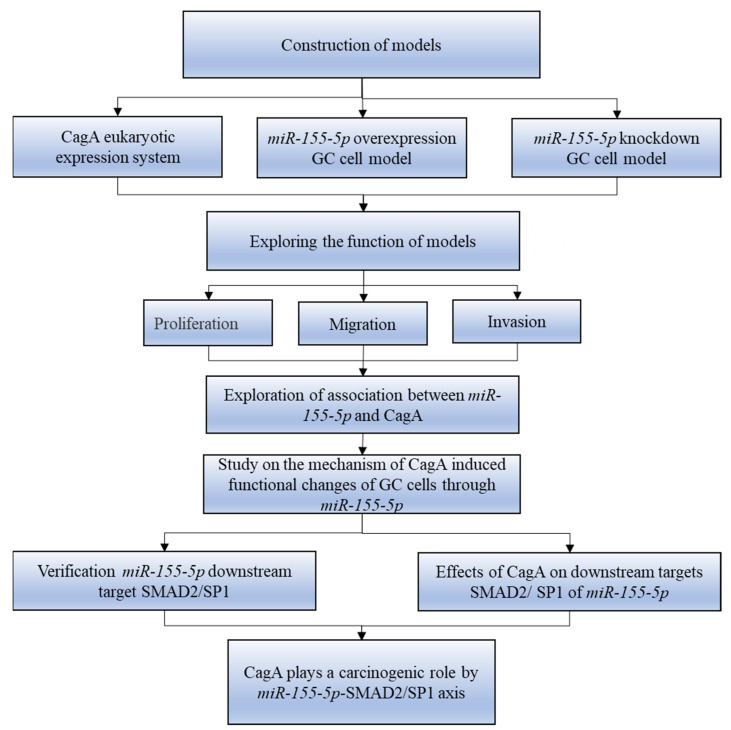
General diagram summarizing the discovery and pathway.

In summary, this study explored a novel pathway in which CagA caused changes to the function of GC cells. CagA influenced the expression of SMAD2/SP1 protein through *miR-155-5p*, which, in turn, promoted cell proliferation, migration, and invasion (Scheme 1) 

This study associated CagA and miRNA molecules with the function of the development and progression in GC, which is innovation. It is intended to provide a certain theoretical basis for research on the treatment of CagA-associated GC, and also provide a basis for research on the current relevant aspects in the field of GC. However, this study is still far from achieving this goal. There are several shortcomings of this study, which need to be improved in depth. Our study was only conducted based on cell experiments and did not validate the findings at the population level, which is required for the study to achieve greater value. Future research will be performed at the population level to further illustrate this conclusion.

## Data Availability

Not applicable.

## Supplementary Materials

The following supporting information can be downloaded at: https://www.mdpi.com/article/10.3390/pathogens11080846/s1, File S1: Plasmid Synthesis Report; File S2: Sanger sequencing alignment file; Table S1: *miR-155-5p* interference sequences; Table S2: Sequence-specific primers.
