# Supplementary material for: Helicobacter pylori CagA Protein Regulating the Biological Characteristics of Gastric Cancer through the miR-155-5p/SMAD2/SP1 axis
Source: Pathogens. 2022 Jul 28;11(8):846. doi: 10.3390/pathogens11080846 (PMC9414533; doi:10.3390/pathogens11080846)
Supplement: Supplementary file 1 [file pathogens-11-00846-s001.zip › File S1.pdf]

Gene synthesis report

Gene name: ABSX01000003.1 (CagA)

Gene length: 3552 bp

Cloning vector: pcDNA3.1

Cloning site: EcoRI/XhoI

Clonal vector resistance: Amp

Sample	Sample concentration (ng/μl)	A260/A280	A260/A230	A260	A280
CagA	827.61	1.92	2.22	16.55	8.63

Quality Control:

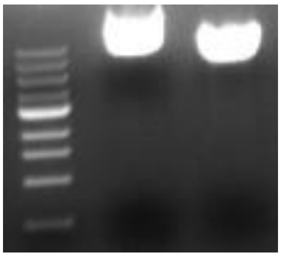	Line2: digested with plasmid +EcoRI XhoI<br>Line1: ladder	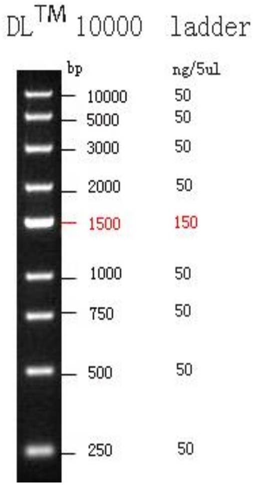

Structure diagram:

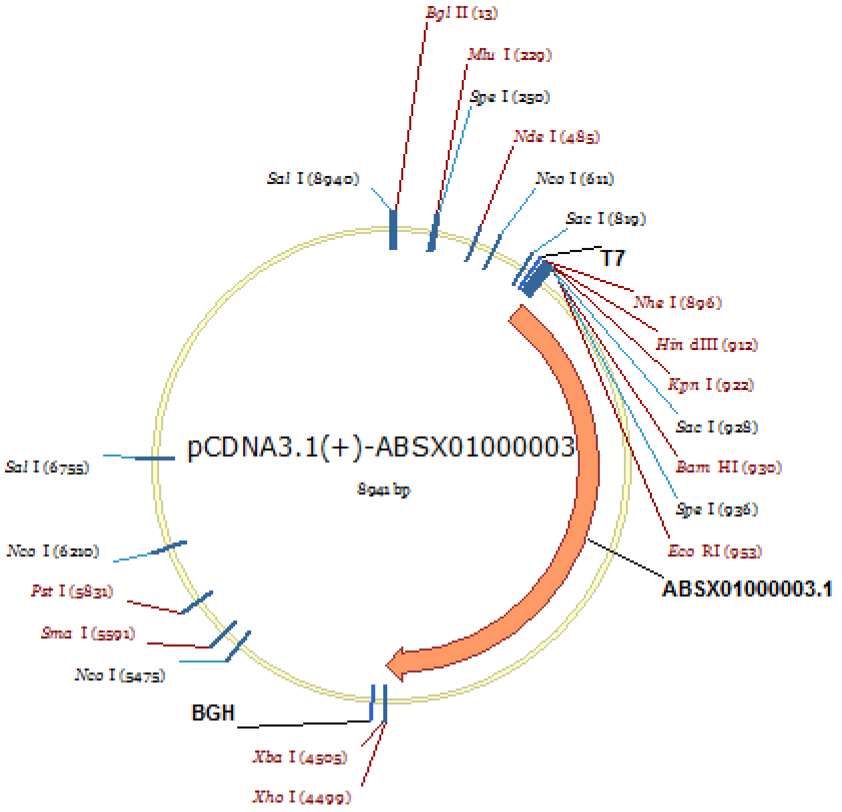

Full sequence: (Forward)

1	GACGGATCGG	GAGATCTCCC	GATCCCCTAT	GGTGCACTCT	CAGTACAATC
51	TGCTCTGATG	CCGCATAGTT	AAGCCAGTAT	CTGCTCCCTG	CTTGTGTGTT
101	GGAGGTCGCT	GAGTAGTGCG	CGAGCAAAAT	TTAAGCTACA	ACAAGGCAAG
151	GCTTGACCGA	CAATTGCATG	AAGAATCTGC	TTAGGGTTAG	GCGTTTTGCG
201	CTGCTTCGCG	ATGTACGGGC	CAGATATACG	CGTTGACATT	GATTATTGAC
251	TAGTTATTAA	TAGTAATCAA	TTACGGGGTC	ATTAGTTCAT	AGCCCATATA
301	TGGAGTTCCG	CGTTACATAA	CTTACGGTAA	ATGGCCCGCC	TGGCTGACCG
351	CCCAACGACC	CCCGCCCATT	GACGTCAATA	ATGACGTATG	TTCCCATAGT
401	AACGCCAATA	GGGACTTTCC	ATTGACGTCA	ATGGGTGGAG	TATTTACGGT
451	AAACTGCCCA	CTTGGCAGTA	CATCAAGTGT	ATCATATGCC	AAGTACGCCC
501	CCTATTGACG	TCAATGACGG	TAAATGGCCC	GCCTGGCATT	ATGCCCAGTA
551	CATGACCTTA	TGGGACTTTC	CTACTTGGCA	GTACATCTAC	GTATTAGTCA
601	TCGCTATTAC	CATGGTGATG	CGGTTTTGGC	AGTACATCAA	TGGGCGTGGA
651	TAGCGGTTTG	ACTCACGGGG	ATTTCCAAGT	CTCCACCCCA	TTGACGTCAA
701	TGGGAGTTTG	TTTTGGCACC	AAAATCAACG	GGACTTTCCA	AAATGTCGTA
751	ACAACTCCGC	CCCATTGACG	CAAATGGGCG	GTAGGCGTGT	ACGGTGGGAG
801	GTCTATATAA	GCAGAGCTCT	CTGGCTAACT	AGAGAACCCA	CTGCTTACTG
851	GCTTATCGAA	ATTAATACGA	CTCACTATAG	GGAGACCCAA	GCTGGCTAGC
901	GTTTAAACTT	AAGCTTGGTA	CCGAGCTCGG	ATCCACTAGT	CCAGTGTGGT
951	GGAATTCGCC	ACCATGATTA	ACGAGACCAT	TGACCAGACC	ACCACCCCCG
1001	ACCAGACCCC	AAACCAGACC	GGCTTCGTGC	CTCAGCGGTT	CATTAACAAC
1051	CTACAGGTGG	CCTTCATTAA	GGTGGACAAC	GCCGTGGCCT	CTTTCGACCC
1101	CGACCAGAAG	CCTATTGTGG	ACAAGAACGA	CCGCGACAAC	AGACAGGCCT
1151	TCGAGAAAAT	ATCTCAGCTG	CGGGAGGAGT	ACGCCAACAA	GGCCATTAAG
1201	AACCCAGCCA	AGAAGAACCA	GTACTTCTCT	GACTTCATTA	ACAAGTCTAA
1251	CGACCTCATT	AACAAGGACA	ACCTGATTGC	CGTGGACTCC	TCTGTGGAGT
1301	CTTTCCGGAA	GTTCGGCGAC	CAGCGATACC	AGATTTTCAC	ATCTTGGGTG

1351	TCCCTCCAAA	AGGACCCATC	TAAGATTAAC	ACACAGCAGA	TTCGGAACTT
1401	CATGGAGAAC	ATCATTCAGC	CCCCTATCAG	CGACGACAAG	GAGAAGGCCG
1451	AGTTCCTGCG	GTCTGCCAAG	CAGTCCTTCG	CCGGCATCAT	TATTGGCAAC
1501	CAGATCCGGT	CTGACCAGAA	GTTCATGGGC	GTGTTCGACG	AGTCTCTGAA
1551	GGAGAGACAG	GAGGCCGAGA	AGAACGCCGA	GCCCGCCGGC	GGCGACTGGC
1601	TGGACATTTT	CCTGTCTTTC	GTGTTCAACA	AGAAGCAGTC	TAGCGACCTC
1651	AAGGAGACAC	TGAACCAGGA	GCCCCGCCCC	GACTTCGAGC	AGAACCTCGC
1701	CACCACCACC	ACCGACATCC	AGGGCCTCCC	CCCAGAGGCA	CGCGACCTGC
1751	TGGACGAGCG	GGGCAACTTC	TTCAAGTTCA	CACTGGGCGA	CGTGGAGATG
1801	CTCGACGTGG	AGGGCGTGGC	CGACAAGGAC	CCAAACTACA	AGTTCAACCA
1851	GCTGCTCATC	CACAACAACG	CCCTGTCCAG	CGTGCTGATG	GGCGGCCACT
1901	CTAACATTGA	GCCCGAGAAG	GTGTCCCTGC	TGTACGGCAA	CAACGGCGGC
1951	CCCGAGGCCC	GGCACGACTG	GAACGCCACA	GTGGGCTACA	AGGACCAGCA
2001	GGGCAACAAC	GTGGCCACAC	TGATTAACGT	GCACCTGAAC	AACGGCTCTG
2051	GCCTCATTGT	GGCCGGCAAC	GAGGACGGCA	TTAAGAACCC	ATCTTTCTAC
2101	CTGTACAAGG	AGGACCAGCT	CACCGGCCTG	AAGCAGGCCC	TGTCTCAGGA
2151	GGAGATTCAG	AACAAGGTGG	ACTTCATGGA	GTTCCTCGCC	CAGAACAACG
2201	CCAAGCTGGA	CAACCTGTCC	GAGAAGGAGA	AGGAGAAGCT	CCAGACAGAG
2251	ATTGAGAACT	TCCAGAAGGA	CCGGAAGGCC	TACCTCGACG	CCCTGGGCAA
2301	CGACCACATT	GCCTTCGTGT	CCAAGAAGGA	CCCTAAGCAC	CTCGCCCTGG
2351	TGACAGAGTT	CGGCAACGGC	GAGCTGTCCT	ACACACTGAA	GGACTACGGC
2401	AAGAAGCAGG	ACAAGGCCCT	CGACGGCGAG	ACAAAGACCA	CACTTCAAGG
2451	CTCTCTCAAG	TACGACGGCG	TGATGTTCGT	GAACTACTCT	AACTTCAAGT
2501	ACACAAACGC	CTCTAAGTCC	CCAAACAAGG	GCGTGGGCGC	CACAAACGGC
2551	GTGTCCCACT	TAGAGGCCAA	CTTCTCTAAG	GTGGCCGTGT	TCAACCTCCC
2601	AAACCTGAAC	AACCTCGCCA	TCACAAACTA	CATCCGCAGG	GACCTGGAAG
2651	ATAAGCTGTG	GTCTAAGGGC	CTGTCCTCTC	AGGAGGCCAA	CAAGCTGATT
2701	AAGGACTTCC	TCAACTCTAA	CAAGGAGATG	GTGGGCAAGG	TGCTGAACTT
2751	CAACAACGCC	GTGGCCGAGG	CCAAGAACAC	CGGCAACTAC	GACGGCGTGA
2801	AGAAGGCCCA	GAAGGACCTG	GAGAAGTCCC	TGCGGAAGCG	GGAGCACCTT
2851	GAAAAGGAGG	TGGCCAAGAA	GTTGGAATCT	CGGAACGACA	ACAAGAACCG
2901	CATGGAGGCC	AAGGCCCAGG	CCAACTCCCA	GAAGGACAAG	ATTTTCGCCC
2951	TGATTAACAA	GGAGGCCTCT	AAGGAGGCCC	GCGCCGTGGC	CTTCGACCCT
3001	AACCTGAAGG	GCATTGGCTC	TGAGCTGTCT	GACAAGCTGG	AGAACATTAA
3051	CAAGAACCTG	AAGGACTTCG	GCAAGTCTTT	CGACGAACTA	AAGAACGGCA
3101	AGAACAACGA	CTTCTCTAAG	GCCGAGGAGA	CACTGAAGGC	CCTCAAGGAC
3151	TCCGTGAAGG	ACCTGGGCAT	TAACCCCGAG	TGGATTTCTA	AGATTGAGAA
3201	CCTCAACGCC	GCCCTCAACG	ACTTCAAGAA	CGGCAAGAAC	AAGGACTTCT
3251	CTAAGGTGAT	CCAGGCCAAG	TCTGACCTAG	AAAACTCCAT	CAAGGACGTG
3301	ATCATTAACC	AGAAGATCAC	AGACAAGGTG	AACAACCTGA	ACCAGGCCGT
3351	GTCTGAGACA	AAGCTCACCG	GCGACTTCTC	TAAGGTGGAG	CAGGCCCTCG
3401	CCGAACTAAA	GAACCTCAGC	CTCGACCTGG	GCAAGAACTC	CGACCTCCAG
3451	AAGTCCGTGA	AGAACGGCGT	GAACCGCACA	CTGGTGGGCA	ACGGCCTGTC
3501	TAAGACAGAG	GCCACAAAGC	TCACAAAGAA	CTTCAGCGAC	ATCCGGAAGG
3551	AACTGAACGA	GAAGCTGTTC	GGCAACTCTA	ACAACAACAA	CAACGGCCTC
3601	AAGAACAACA	CAGAGCCTAT	TTACGCCCAG	GTGAACAAGA	AAAAGACAGG
3651	CCAGGTGGCC	TCCCCTGAGG	AGCCTATTTA	CGCCCAGGTG	GCCAAGAAGG
3701	TGTCTGCCAA	GATTGACAGA	CTGAACGAGG	CCACATCTGC	CATTAACCGG
3751	AAGATTGACC	GGATTAACAA	GATTGCCTCC	GCCGGCAAGG	GCGTGGGCGG
3801	CTTCAGCGGC	GCCGGCCGCT	CCGCCTCTCC	CGAGCCTATT	TACGCCACCA
3851	TTGACTTCGA	CGAGGCCAAC	CAGGCCGGCT	TCCCACTGCG	CCGATCCGCC
3901	GCCGTGAACG	ACCTGTCTAA	GGTGGGCCTG	AGTCGAGAGC	AGGAGCTTAC
3951	CCGCCGCATT	GGCGACCTGT	CTCAGGCCGT	GAGCGAGGCC	AAGACAGGCC
4001	ACTTCGACAA	CCTGGAGCAG	AAGATTGACG	AGCTGAAGGA	CTCTACAAAG
4051	AAGAACGCCC	TGAAGCTGTG	GGTGGAGTCT	GCCAAGCAGG	TGCCTACCGG
4101	CTTGCAGGCC	AAGCTGGACA	ACTACGCCAC	AAACTCCCAC	ACCCGCATTA
4151	ACTCTAACGT	GCAGCACGGC	GCCATTAACG	AGAAGGCCAC	GGGGATGCTG
4201	ACACAGAAGA	ACCCCGAGTG	GCTCAAGCTG	GTGAACGACA	AGATTGTGGC
4251	CCACAACGTG	GGCTCTACCC	CACTGTCTGA	GTACGACAAG	ATCGGCTTCA
4301	ACCAGAAGAA	CATGAAGGAC	TACTCCGACT	CTTTCAAGTT	CAGCACAAAG
4351	CTCAACAACG	CCGTGAAGGA	CATTAAGTCT	TCTTTCGTGC	AGTTCCTCAC
4401	AAACACATTC	TCCACCGGCT	CTTACTCCCT	GATTAAGGCC	AACGCCGAGC

4451	ACGGCGTGAA	GAACACAAAC	ACAAAGGGCG	GCTTCCAGAA	GTCTTGACTC
4501	GAGTCTAGAG	GGCCCGTTTA	AACCCGCTGA	TCAGCCTCGA	CTGTGCCTTC
4551	TAGTTGCCAG	CCATCTGTTG	TTTGCCCCTC	CCCCGTGCCT	TCCTTGACCC
4601	TGGAAGGTGC	CACTCCCACT	GTCCTTTCCT	AATAAAATGA	GGAAATTGCA
4651	TCGCATTGTC	TGAGTAGGTG	TCATTCTATT	CTGGGGGGTG	GGGTGGGGCA
4701	GGACAGCAAG	GGGGAGGATT	GGGAAGACAA	TAGCAGGCAT	GCTGGGGATG
4751	CGGTGGGCTC	TATGGCTTCT	GAGGCGGAAA	GAACCAGCTG	GGGCTCTAGG
4801	GGGTATCCCC	ACGCGCCCTG	TAGCGGCGCA	TTAAGCGCGG	CGGGTGTGGT
4851	GGTTACGCGC	AGCGTGACCG	CTACACTTGC	CAGCGCCCTA	GCGCCCGCTC
4901	CTTTCGCTTT	CTTCCCTTCC	TTTCTCGCCA	CGTTCGCCGG	CTTTCCCCGT
4951	CAAGCTCTAA	ATCGGGGGCT	CCCTTTAGGG	TTCCGATTTA	GTGCTTTACG
5001	GCACCTCGAC	CCCAAAAAAC	TTGATTAGGG	TGATGGTTCA	CGTAGTGGGC
5051	CATCGCCCTG	ATAGACGGTT	TTTCGCCCTT	TGACGTTGGA	GTCCACGTTC
5101	TTTAATAGTG	GACTCTTGTT	CCAAACTGGA	ACAACACTCA	ACCCTATCTC
5151	GGTCTATTCT	TTTGATTTAT	AAGGGATTTT	GCCGATTTCG	GCCTATTGGT
5201	TAAAAAATGA	GCTGATTTAA	CAAAAATTTA	ACGCGAATTA	ATTCTGTGGA
5251	ATGTGTGTCA	GTTAGGGTGT	GGAAAGTCCC	CAGGCTCCCC	AGCAGGCAGA
5301	AGTATGCAAA	GCATGCATCT	CAATTAGTCA	GCAACCAGGT	GTGGAAAGTC
5351	CCCAGGCTCC	CCAGCAGGCA	GAAGTATGCA	AAGCATGCAT	CTCAATTAGT
5401	CAGCAACCAT	AGTCCCGCCC	CTAACTCCGC	CCATCCCGCC	CCTAACTCCG
5451	CCCAGTTCCG	CCCATTCTCC	GCCCCATGGC	TGACTAATTT	TTTTTATTTA
5501	TGCAGAGGCC	GAGGCCGCCT	CTGCCTCTGA	GCTATTCCAG	AAGTAGTGAG
5551	GAGGCTTTTT	TGGAGGCCTA	GGCTTTTGCA	AAAAGCTCCC	GGGAGCTTGT
5601	ATATCCATTT	TCGGATCTGA	TCAAGAGACA	GGATGAGGAT	CGTTTCGCAT
5651	GATTGAACAA	GATGGATTGC	ACGCAGGTTC	TCCGGCCGCT	TGGGTGGAGA
5701	GGCTATTCGG	CTATGACTGG	GCACAACAGA	CAATCGGCTG	CTCTGATGCC
5751	GCCGTGTTCC	GGCTGTCAGC	GCAGGGGCGC	CCGGTTCTTT	TTGTCAAGAC
5801	CGACCTGTCC	GGTGCCCTGA	ATGAACTGCA	GGACGAGGCA	GCGCGGCTAT
5851	CGTGGCTGGC	CACGACGGGC	GTTCCTTGCG	CAGCTGTGCT	CGACGTTGTC
5901	ACTGAAGCGG	GAAGGGACTG	GCTGCTATTG	GGCGAAGTGC	CGGGGCAGGA
5951	TCTCCTGTCA	TCTCACCTTG	CTCCTGCCGA	GAAAGTATCC	ATCATGGCTG
6001	ATGCAATGCG	GCGGCTGCAT	ACGCTTGATC	CGGCTACCTG	CCCATTCGAC
6051	CACCAAGCGA	AACATCGCAT	CGAGCGAGCA	CGTACTCGGA	TGGAAGCCGG
6101	TCTTGTCGAT	CAGGATGATC	TGGACGAAGA	GCATCAGGGG	CTCGCGCCAG
6151	CCGAACTGTT	CGCCAGGCTC	AAGGCGCGCA	TGCCCGACGG	CGAGGATCTC
6201	GTCGTGACCC	ATGGCGATGC	CTGCTTGCCG	AATATCATGG	TGGAAAATGG
6251	CCGCTTTTCT	GGATTCATCG	ACTGTGGCCG	GCTGGGTGTG	GCGGACCGCT
6301	ATCAGGACAT	AGCGTTGGCT	ACCCGTGATA	TTGCTGAAGA	GCTTGGCGGC
6351	GAATGGGCTG	ACCGCTTCCT	CGTGCTTTAC	GGTATCGCCG	CTCCCGATTC
6401	GCAGCGCATC	GCCTTCTATC	GCCTTCTTGA	CGAGTTCTTC	TGAGCGGGAC
6451	TCTGGGGTTC	GAAATGACCG	ACCAAGCGAC	GCCCAACCTG	CCATCACGAG
6501	ATTTCGATTC	CACCGCCGCC	TTCTATGAAA	GGTTGGGCTT	CGGAATCGTT
6551	TTCCGGGACG	CCGGCTGGAT	GATCCTCCAG	CGCGGGGATC	TCATGCTGGA
6601	GTTCTTCGCC	CACCCCAACT	TGTTTATTGC	AGCTTATAAT	GGTTACAAAT
6651	AAAGCAATAG	CATCACAAAT	TTCACAAATA	AAGCATTTTT	TTCACTGCAT
6701	TCTAGTTGTG	GTTTGTCCAA	ACTCATCAAT	GTATCTTATC	ATGTCTGTAT
6751	ACCGTCGACC	TCTAGCTAGA	GCTTGGCGTA	ATCATGGTCA	TAGCTGTTTC
6801	CTGTGTGAAA	TTGTTATCCG	CTCACAATTC	CACACAACAT	ACGAGCCGGA
6851	AGCATAAAGT	GTAAAGCCTG	GGGTGCCTAA	TGAGTGAGCT	AACTCACATT
6901	AATTGCGTTG	CGCTCACTGC	CCGCTTTCCA	GTCGGGAAAC	CTGTCGTGCC
6951	AGCTGCATTA	ATGAATCGGC	CAACGCGCGG	GGAGAGGCGG	TTTGCGTATT
7001	GGGCGCTCTT	CCGCTTCCTC	GCTCACTGAC	TCGCTGCGCT	CGGTCGTTCG
7051	GCTGCGGCGA	GCGGTATCAG	CTCACTCAAA	GGCGGTAATA	CGGTTATCCA
7101	CAGAATCAGG	GGATAACGCA	GGAAAGAACA	TGTGAGCAAA	AGGCCAGCAA
7151	AAGGCCAGGA	ACCGTAAAAA	GGCCGCGTTG	CTGGCGTTTT	TCCATAGGCT
7201	CCGCCCCCCT	GACGAGCATC	ACAAAAATCG	ACGCTCAAGT	CAGAGGTGGC
7251	GAAACCCGAC	AGGACTATAA	AGATACCAGG	CGTTTCCCCC	TGGAAGCTCC
7301	CTCGTGCGCT	CTCCTGTTCC	GACCCTGCCG	CTTACCGGAT	ACCTGTCCGC
7351	CTTTCTCCCT	TCGGGAAGCG	TGGCGCTTTC	TCATAGCTCA	CGCTGTAGGT
7401	ATCTCAGTTC	GGTGTAGGTC	GTTCGCTCCA	AGCTGGGCTG	TGTGCACGAA
7451	CCCCCCGTTC	AGCCCGACCG	CTGCGCCTTA	TCCGGTAACT	ATCGTCTTGA
7501	GTCCAACCCG	GTAAGACACG	ACTTATCGCC	ACTGGCAGCA	GCCACTGGTA

7551	ACAGGATTAG	CAGAGCGAGG	TATGTAGGCG	GTGCTACAGA	GTTCTTGAAG
7601	TGGTGGCCTA	ACTACGGCTA	CACTAGAAGA	ACAGTATTTG	GTATCTGCGC
7651	TCTGCTGAAG	CCAGTTACCT	TCGGAAAAAG	AGTTGGTAGC	TCTTGATCCG
7701	GCAAACAAAC	CACCGCTGGT	AGCGGTTTTT	TTGTTTGCAA	GCAGCAGATT
7751	ACGCGCAGAA	AAAAAGGATC	TCAAGAAGAT	CCTTTGATCT	TTTCTACGGG
7801	GTCTGACGCT	CAGTGGAACG	AAAACTCACG	TTAAGGGATT	TTGGTCATGA
7851	GATTATCAAA	AAGGATCTTC	ACCTAGATCC	TTTTAAATTA	AAAATGAAGT
7901	TTTAAATCAA	TCTAAAGTAT	ATATGAGTAA	ACTTGGTCTG	ACAGTTACCA
7951	ATGCTTAATC	AGTGAGGCAC	CTATCTCAGC	GATCTGTCTA	TTTCGTTCAT
8001	CCATAGTTGC	CTGACTCCCC	GTCGTGTAGA	TAACTACGAT	ACGGGAGGGC
8051	TTACCATCTG	GCCCCAGTGC	TGCAATGATA	CCGCGAGACC	CACGCTCACC
8101	GGCTCCAGAT	TTATCAGCAA	TAAACCAGCC	AGCCGGAAGG	GCCGAGCGCA
8151	GAAGTGGTCC	TGCAACTTTA	TCCGCCTCCA	TCCAGTCTAT	TAATTGTTGC
8201	CGGGAAGCTA	GAGTAAGTAG	TTCGCCAGTT	AATAGTTTGC	GCAACGTTGT
8251	TGCCATTGCT	ACAGGCATCG	TGGTGTCACG	CTCGTCGTTT	GGTATGGCTT
8301	CATTCAGCTC	CGGTTCCCAA	CGATCAAGGC	GAGTTACATG	ATCCCCCATG
8351	TTGTGCAAAA	AAGCGGTTAG	CTCCTTCGGT	CCTCCGATCG	TTGTCAGAAG
8401	TAAGTTGGCC	GCAGTGTTAT	CACTCATGGT	TATGGCAGCA	CTGCATAATT
8451	CTCTTACTGT	CATGCCATCC	GTAAGATGCT	TTTCTGTGAC	TGGTGAGTAC
8501	TCAACCAAGT	CATTCTGAGA	ATAGTGTATG	CGGCGACCGA	GTTGCTCTTG
8551	CCCGGCGTCA	ATACGGGATA	ATACCGCGCC	ACATAGCAGA	ACTTTAAAAG
8601	TGCTCATCAT	TGGAAAACGT	TCTTCGGGGC	GAAAACTCTC	AAGGATCTTA
8651	CCGCTGTTGA	GATCCAGTTC	GATGTAACCC	ACTCGTGCAC	CCAACTGATC
8701	TTCAGCATCT	TTTACTTTCA	CCAGCGTTTC	TGGGTGAGCA	AAAACAGGAA
8751	GGCAAAATGC	CGCAAAAAAG	GGAATAAGGG	CGACACGGAA	ATGTTGAATA
8801	CTCATACTCT	TCCTTTTTCA	ATATTATTGA	AGCATTTATC	AGGGTTATTG
8851	TCTCATGAGC	GGATACATAT	TTGAATGTAT	TTAGAAAAAT	AAACAAATAG
8901	GGGTTCCGCG	CACATTTCCC	CGAAAAGTGC	CACCTGACGT	C
8951					
