# Supplementary material for: Helicobacter pylori CagA Protein Regulating the Biological Characteristics of Gastric Cancer through the miR-155-5p/SMAD2/SP1 axis
Source: Pathogens. 2022 Jul 28;11(8):846. doi: 10.3390/pathogens11080846 (PMC9414533; doi:10.3390/pathogens11080846)
Supplement: Supplementary file 1 [file pathogens-11-00846-s001.zip › Table S1 and Table S2.pdf]

Supplement

Table S1 *miR-155-5p* interference sequences

***miR-155-5p* interference**	**Sequence (5′-3′)**
miR-155-5p mimics	5′-UUAAUGCUAAUCGUGAUAGGGGUU-3′
	5′-AACCCCUAUCACGAUUAGCAUUAA-3′
miR-155-5p mimics NC	5′-UCACAACCUCCUAGAAAGAGUAGA-3′
	5′-UCUACUCUUUCUAGGAGGUUGUGA-3′
miR-155-5p inhibitor	5′-AACCCCUAUCACGAUUAGCAUUAA-3′
miR-155-5p inhibitor NC	5′-UCUACUCUUUCUAGGAGGUUGUGA-3′

Table S2 Sequence-specific primers

**Primers**	**Sequence (5′-3′)**
miR-155-5p	Forward: 5′-CCGCTTAATGCTAATCGTGATAGGGGT-3′
CagA	Forward: 5′- AACAACCTCGCCATCACA-3′
	Reverse: 5′- CACCTTGCCCACCATCTC-3′
SMAD2	Forward: 5′- CTCTTCTGGCTCAGTCTGTTAA-3′
	Reverse: 5′- AAGGAGTACTTGTTACCGTCTG-3′
SP1	Forward: 5′- TCACTCCATGGATGAAATGACA-3′
	Reverse: 5′- CAGAGGAGGAAGAGATGATCTG-3′
GAPDH	Forward: 5′- GGGAAACTGTGGCGTGAT-3′
	Reverse: 5′- GAGTGGGTGTCGCTGTTGA-3′

MiRNA reverse primer and U6 internal reference were provided by the miRNA First Strand cDNA Synthesis (Tailing Reaction) from Sangon Biological Company.
