# Supplementary figures and images for: Helicobacter pylori CagA Protein Regulating the Biological Characteristics of Gastric Cancer through the miR-155-5p/SMAD2/SP1 axis
Source: Pathogens. 2022 Jul 28;11(8):846. doi: 10.3390/pathogens11080846 (PMC9414533; doi:10.3390/pathogens11080846)

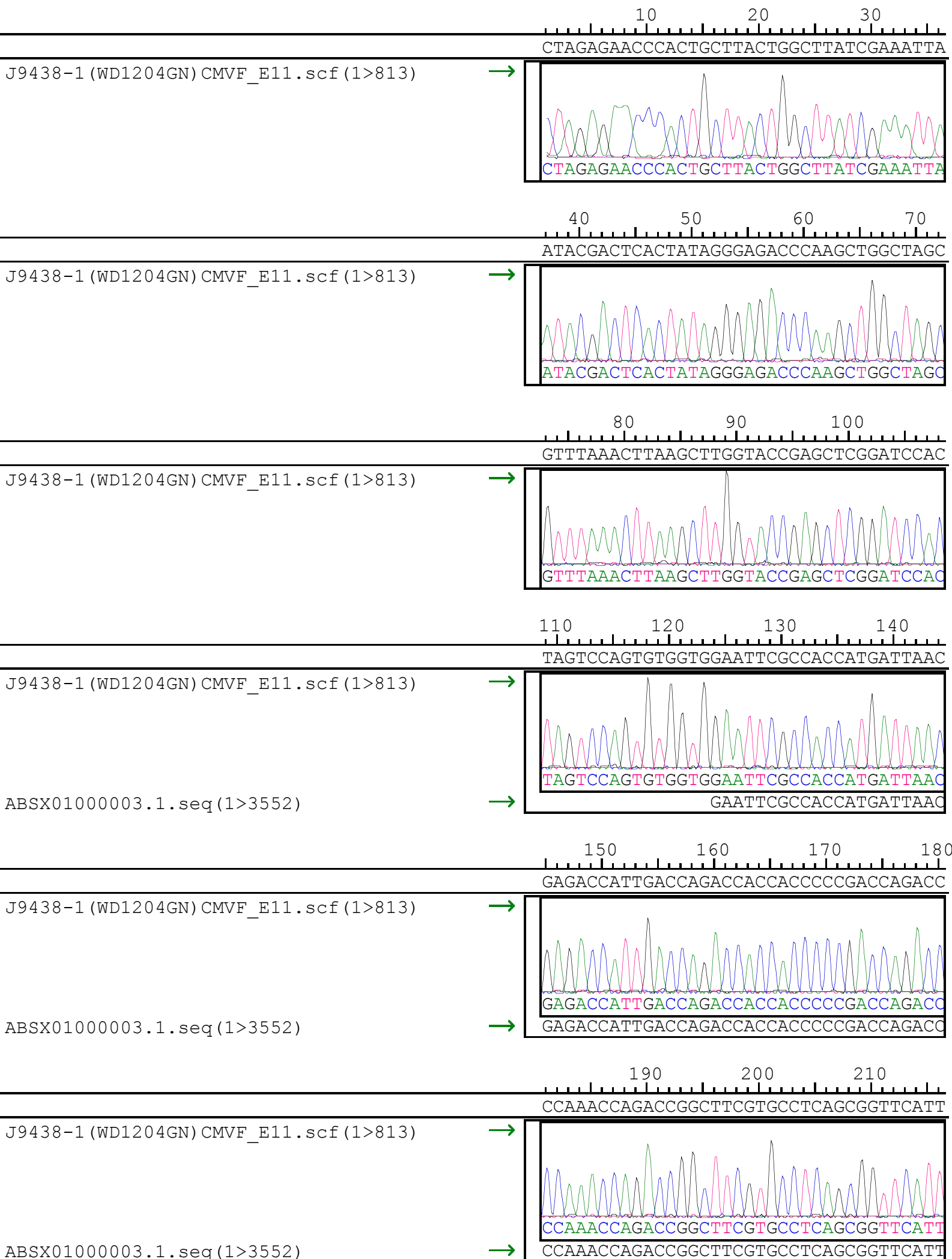

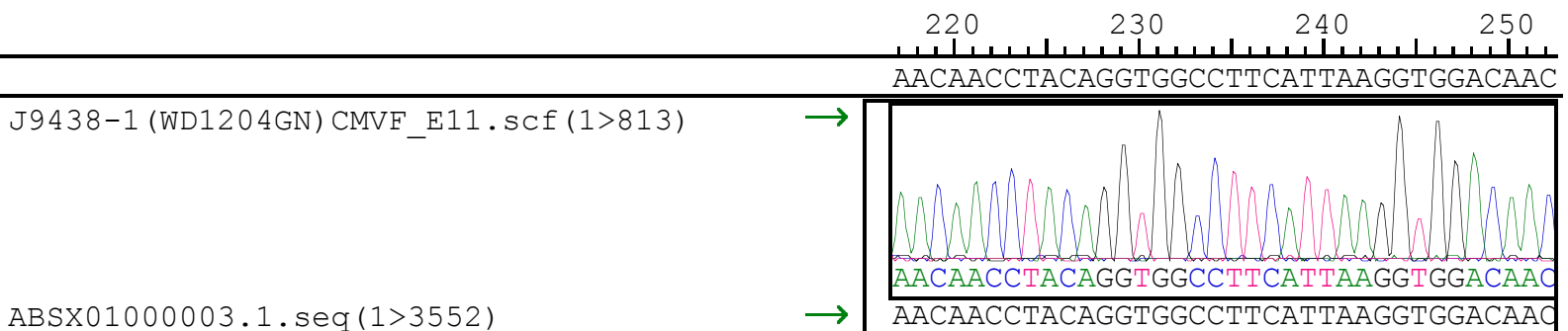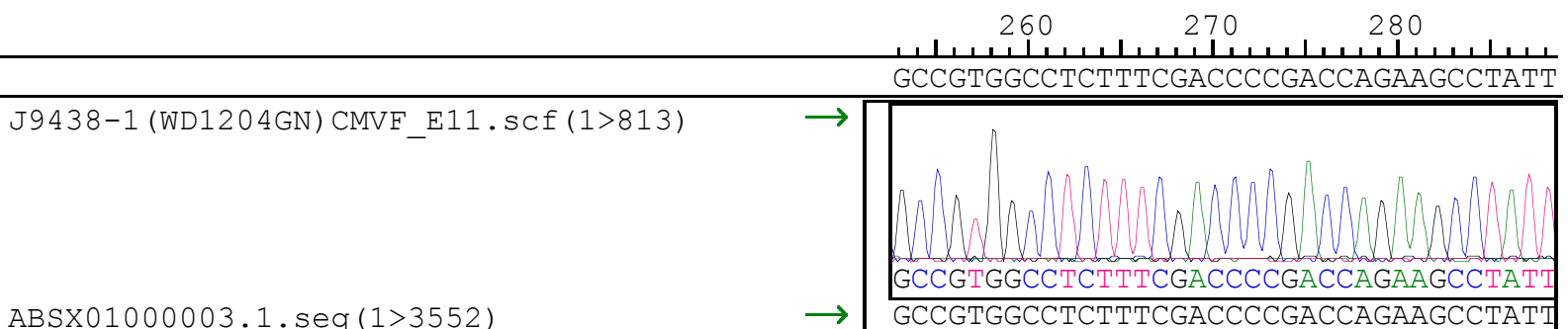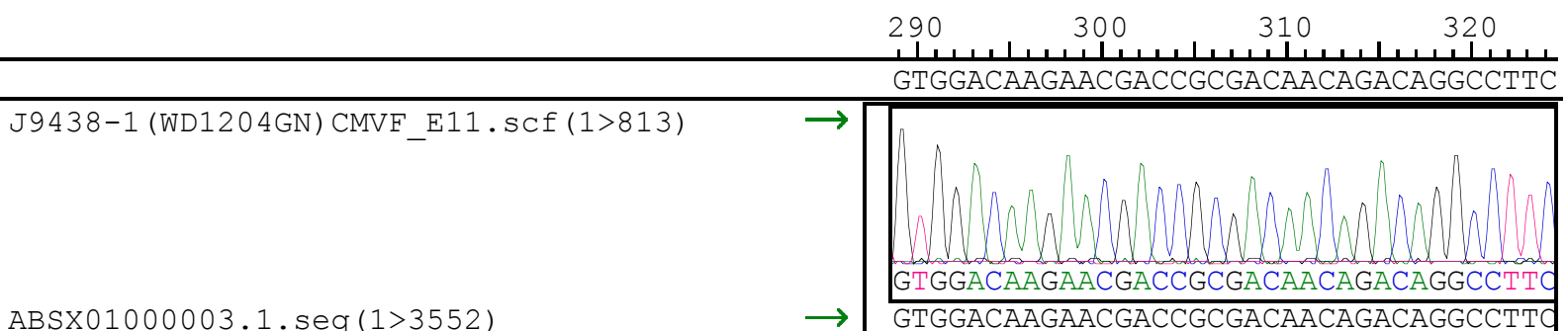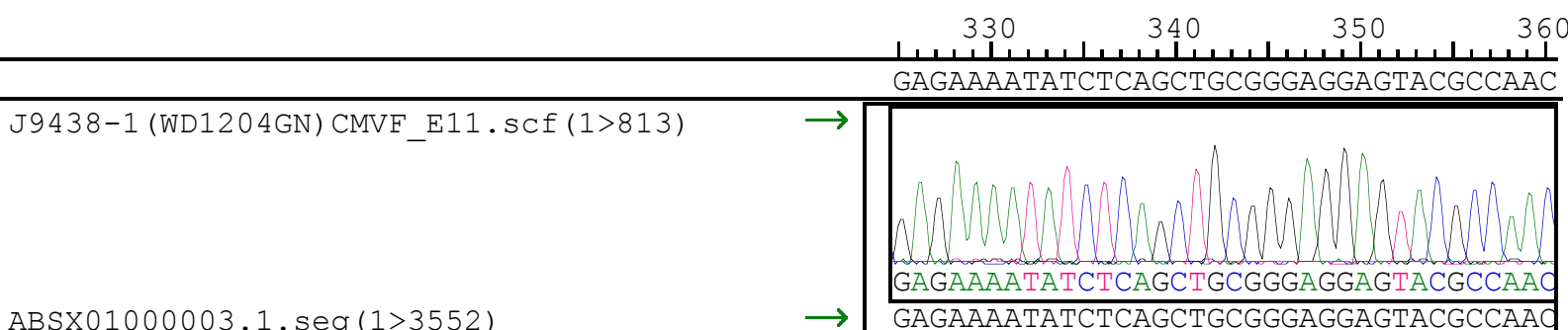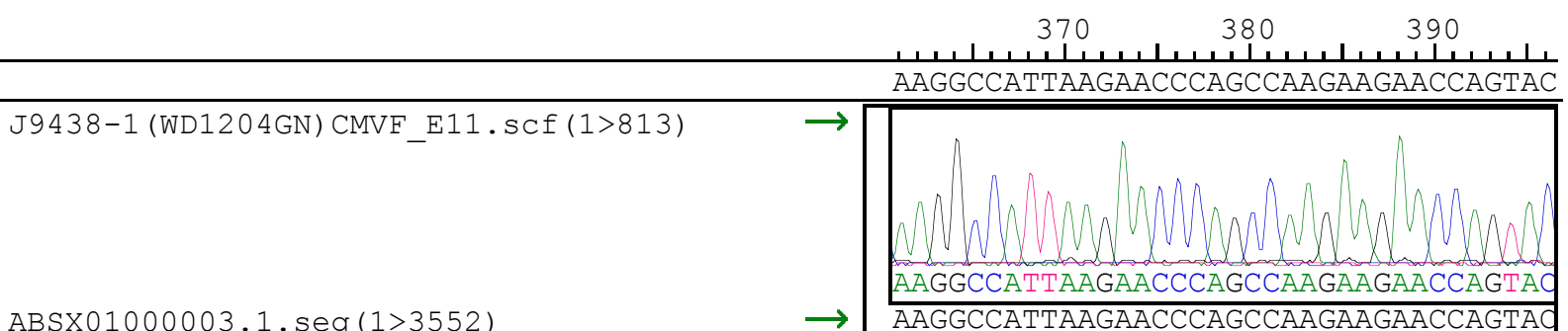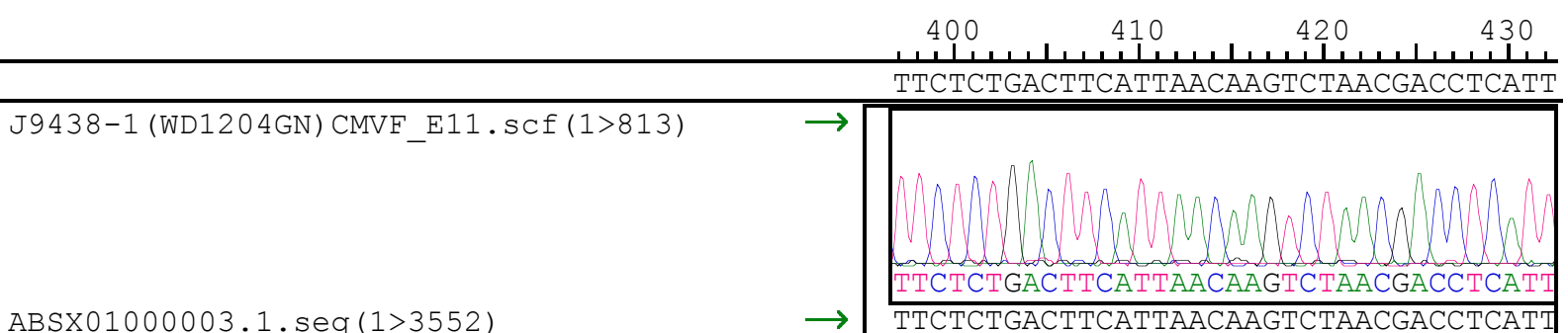

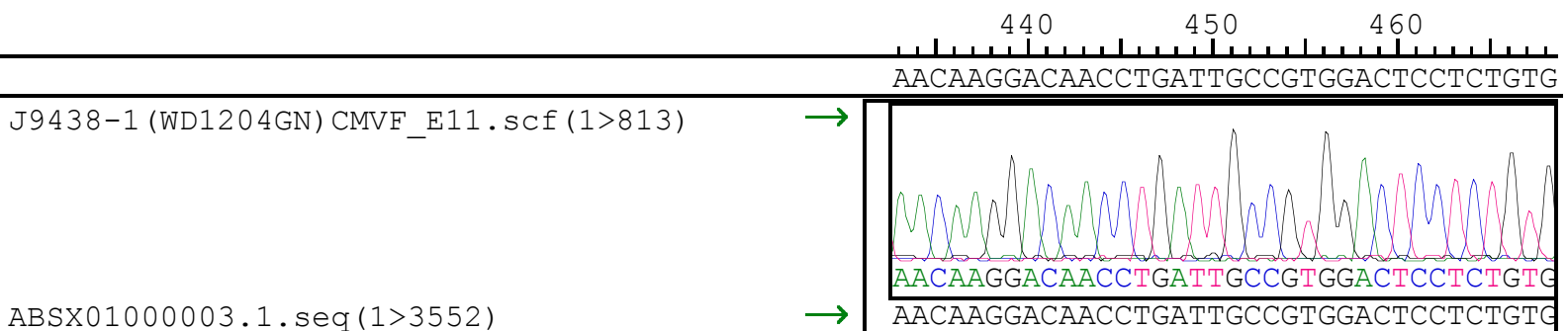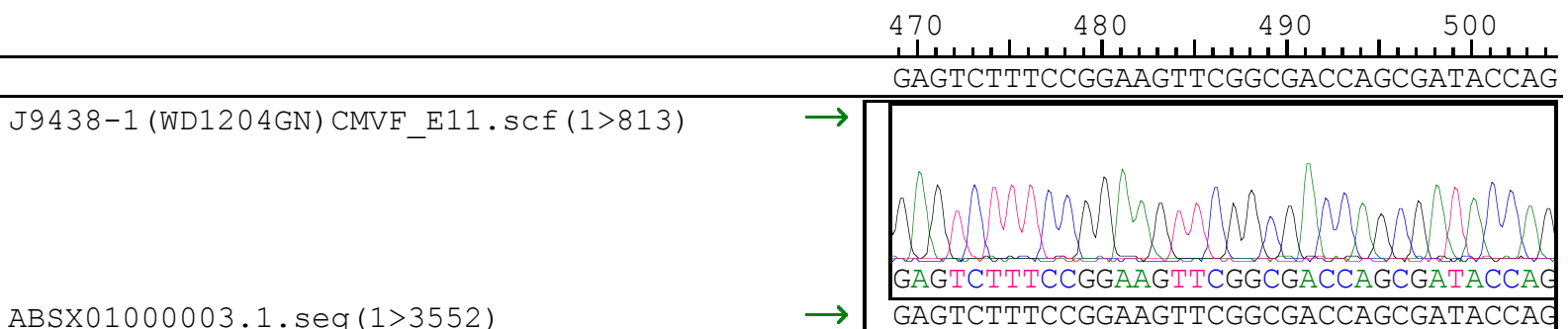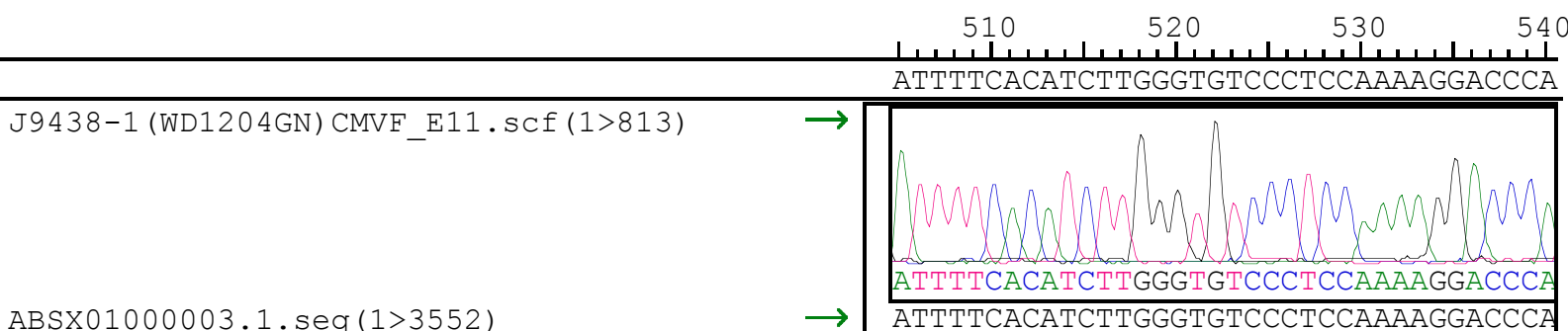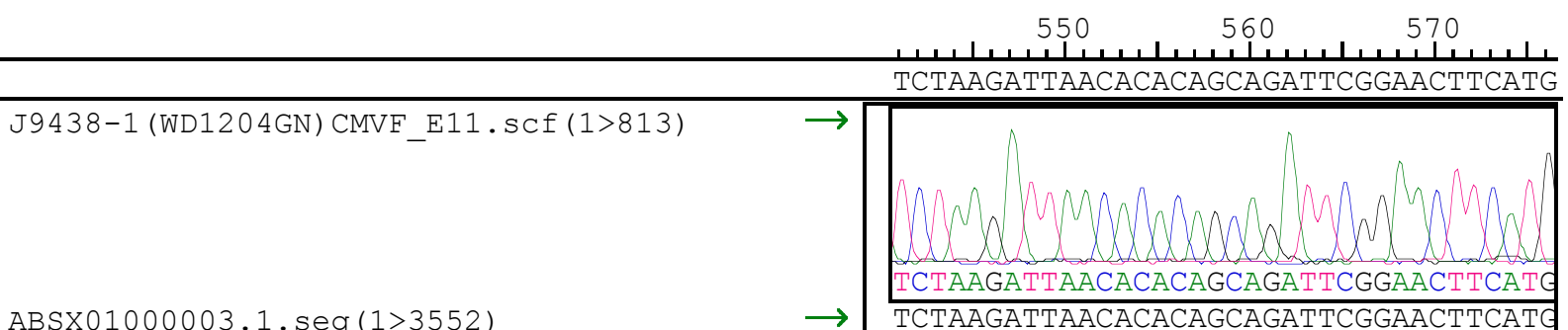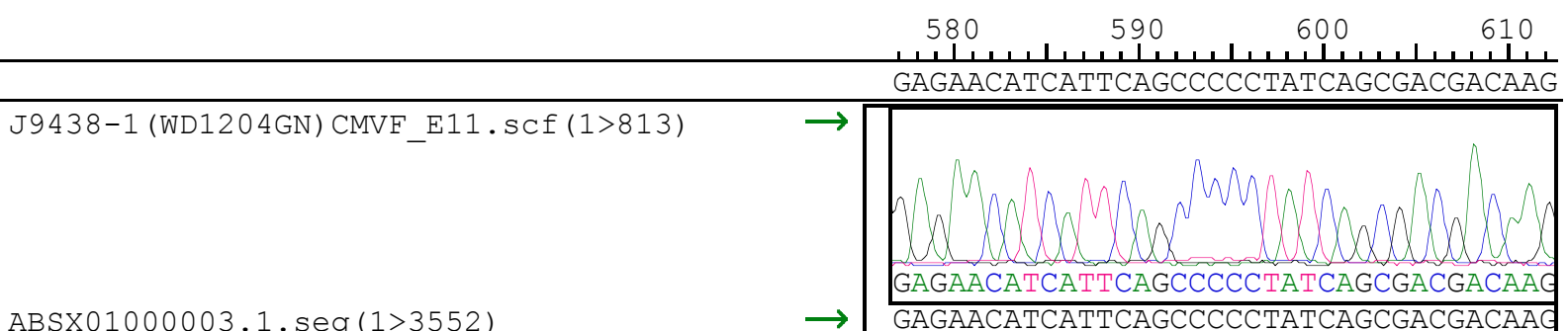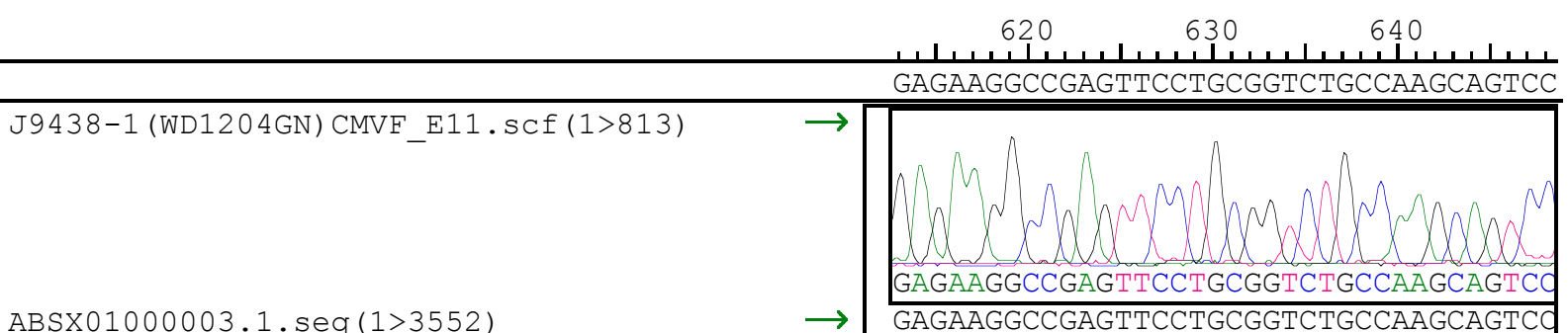

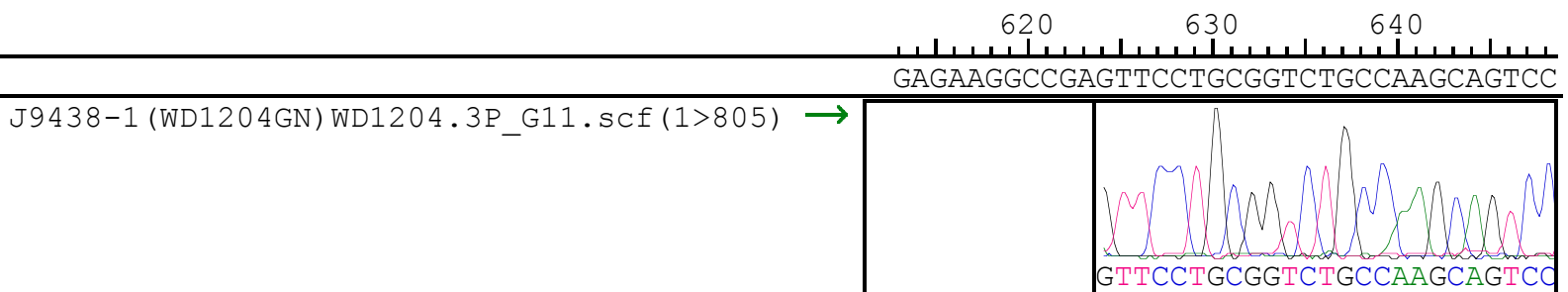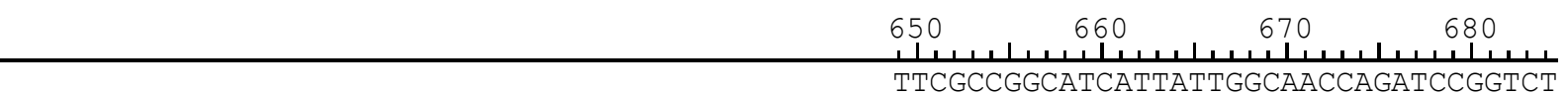

ABSX01000003.1.seq (1>3552) →

J9438-1 (WD1204GN) WD1204.3P\_G11.scf (1>805) →

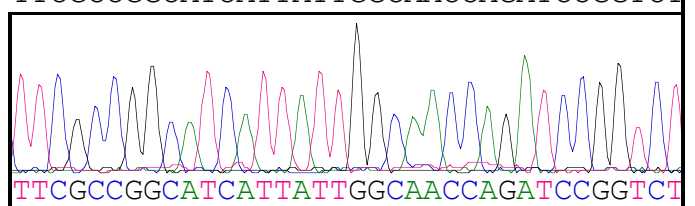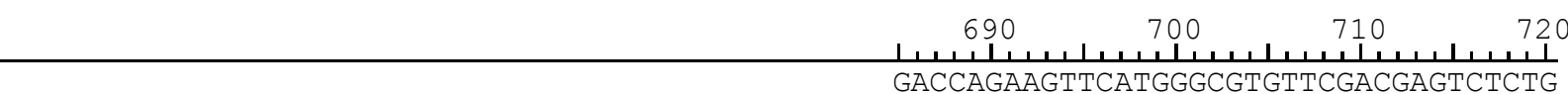

ABSX01000003.1.seq (1>3552) →

J9438-1 (WD1204GN) WD1204.3P\_G11.scf (1>805) →

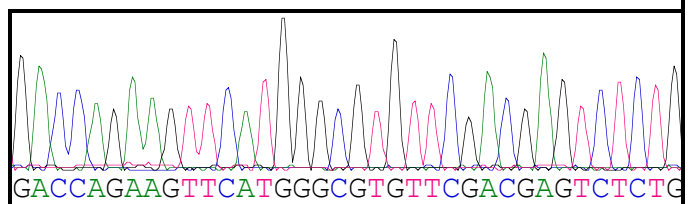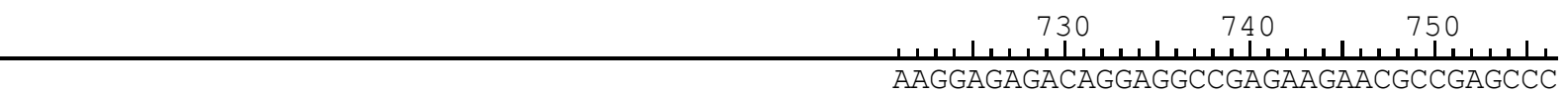

ABSX01000003.1.seq (1>3552) →

J9438-1 (WD1204GN) WD1204.3P\_G11.scf (1>805) →

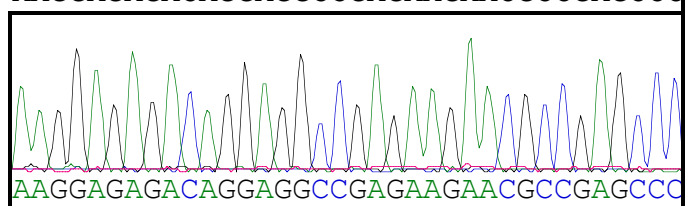

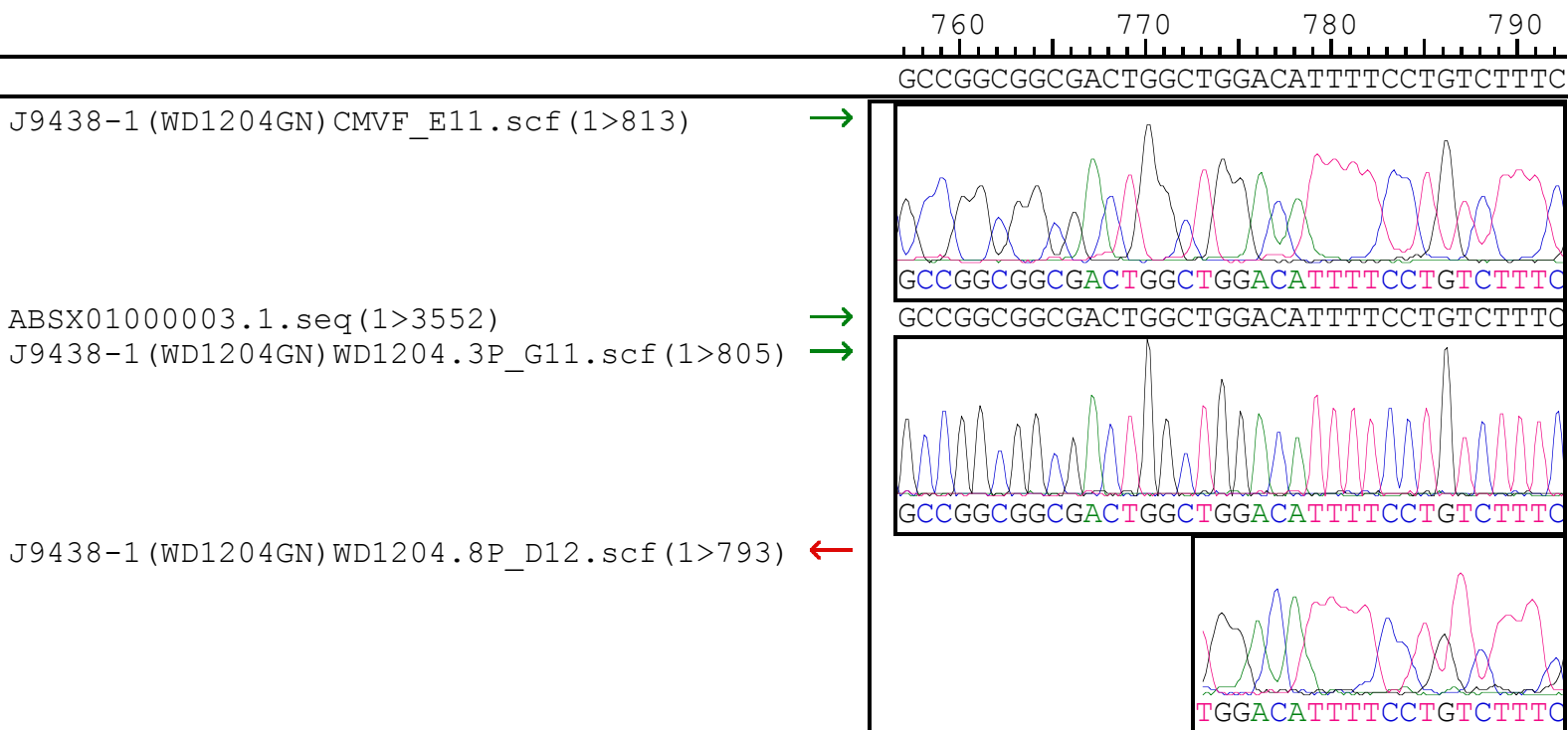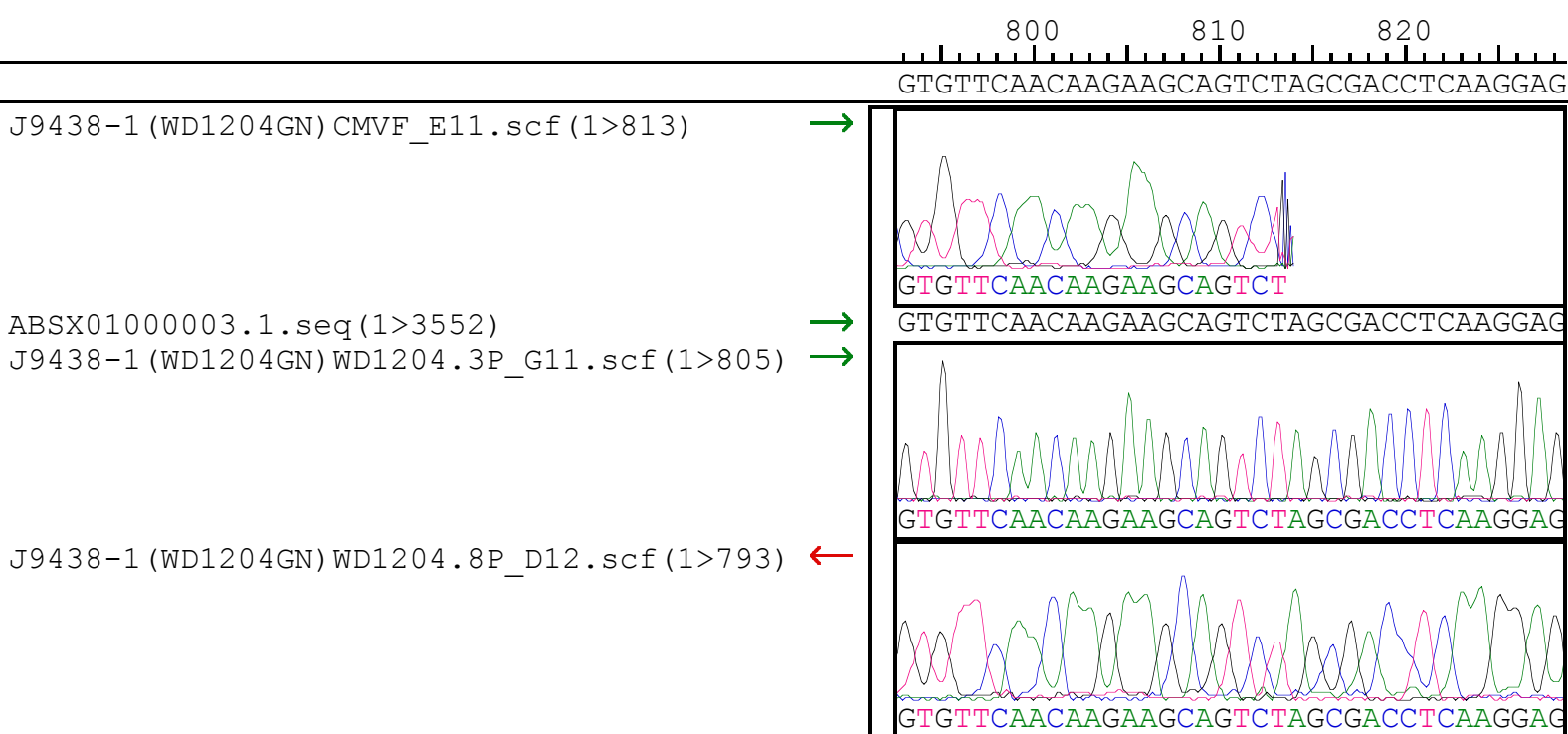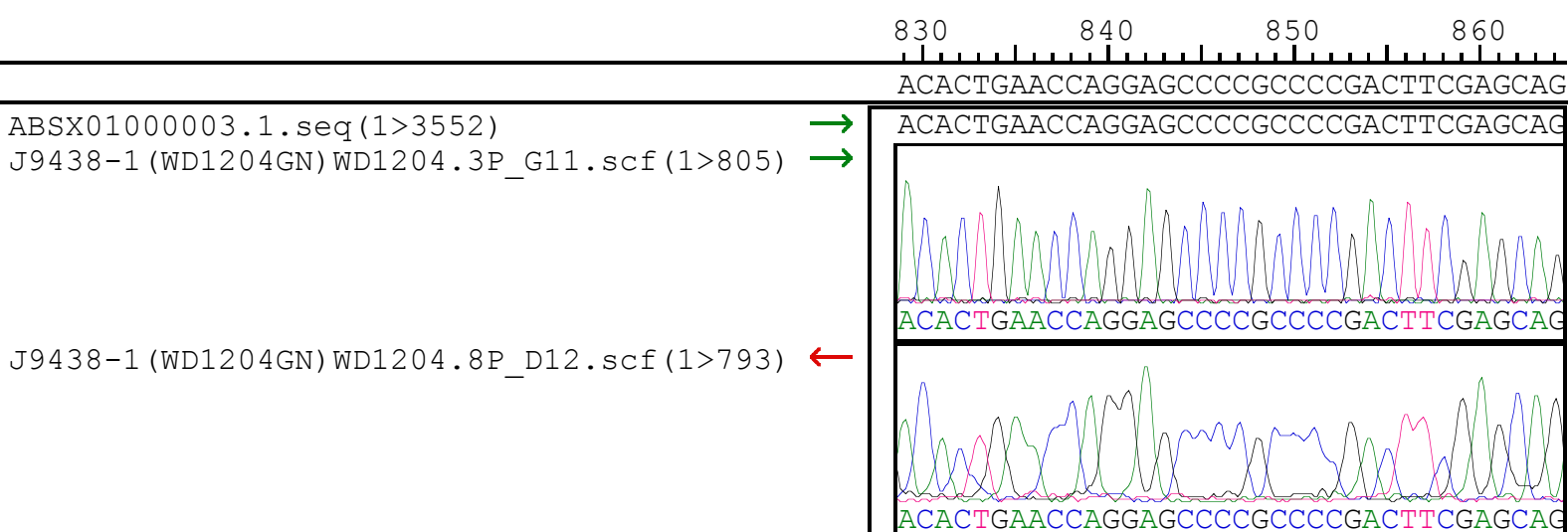

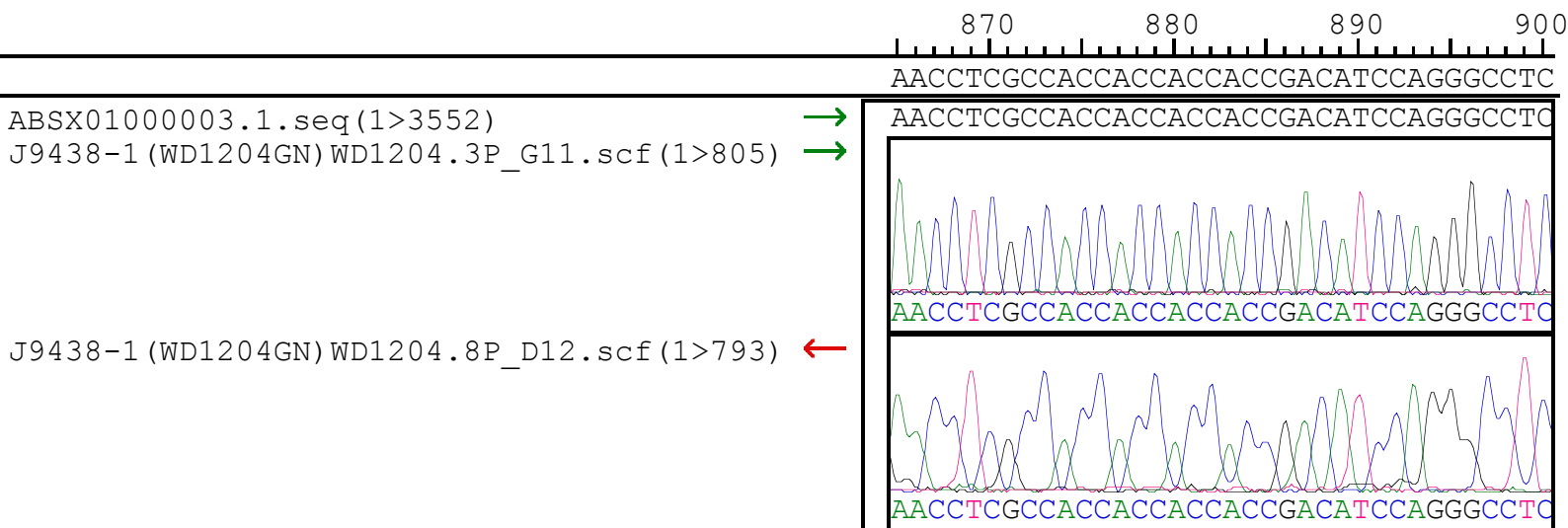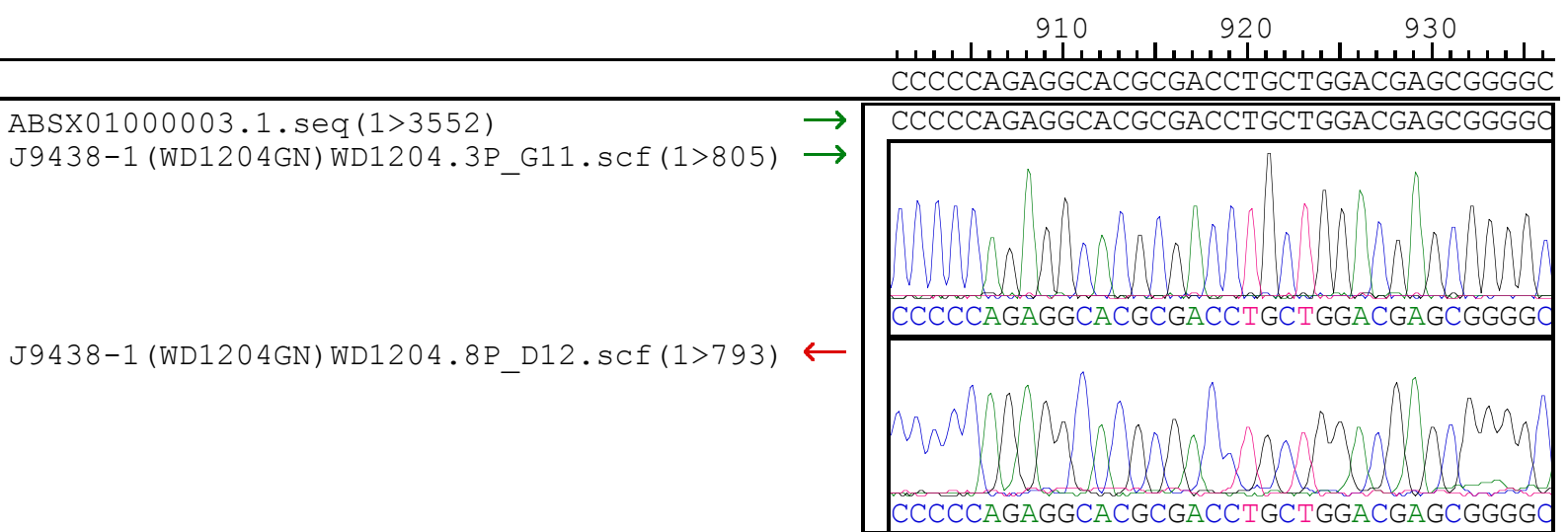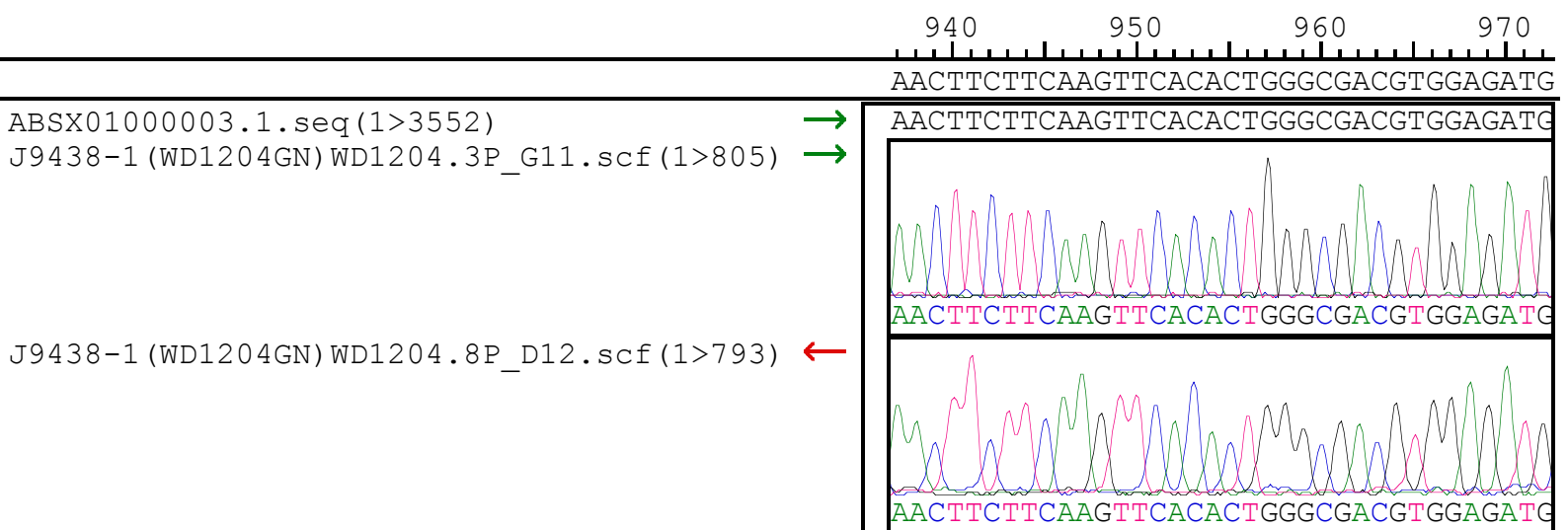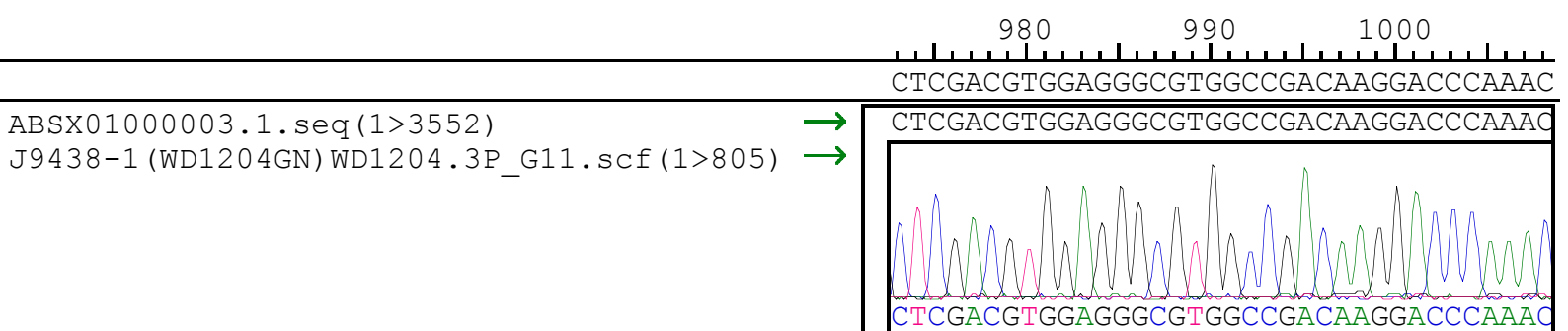

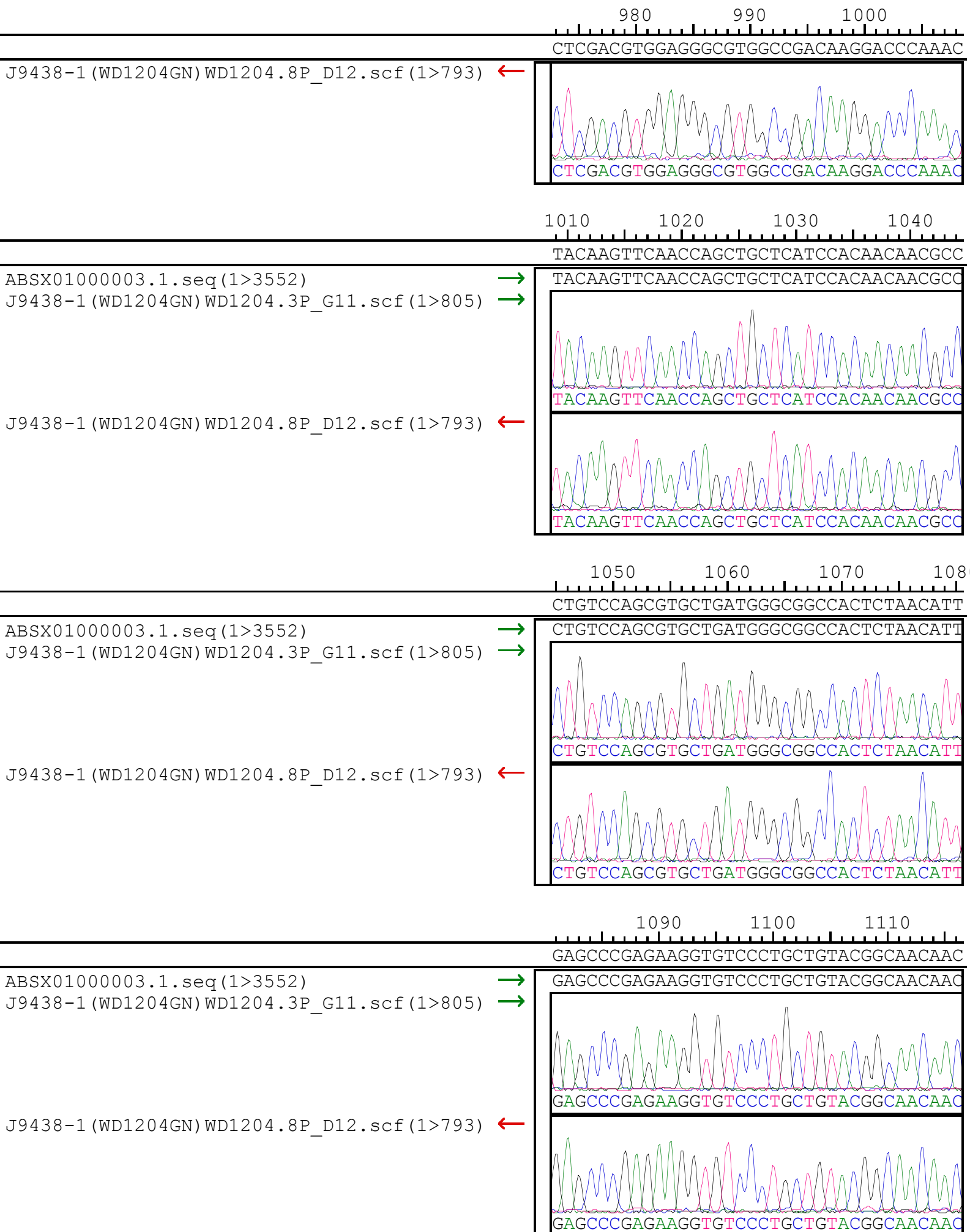

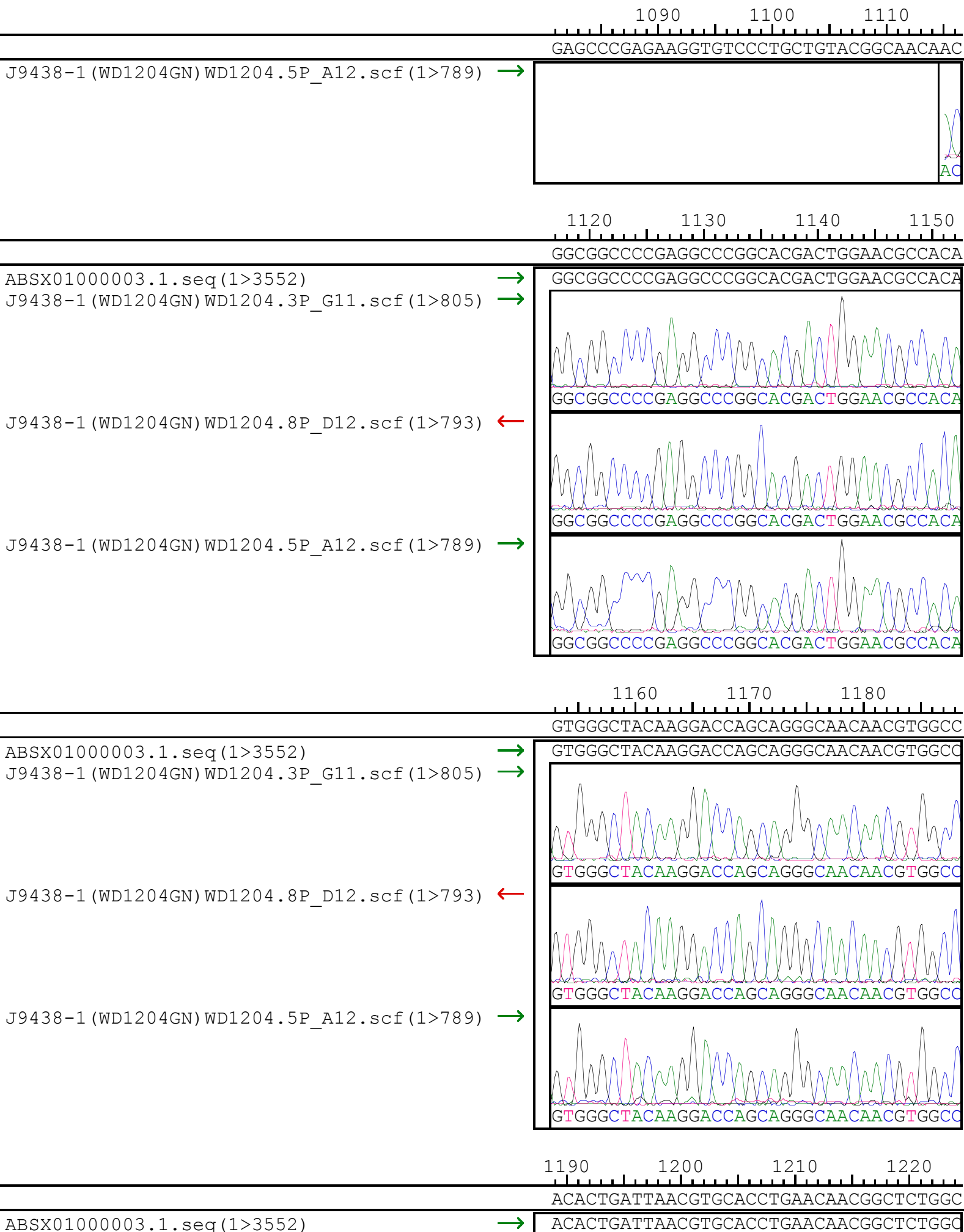

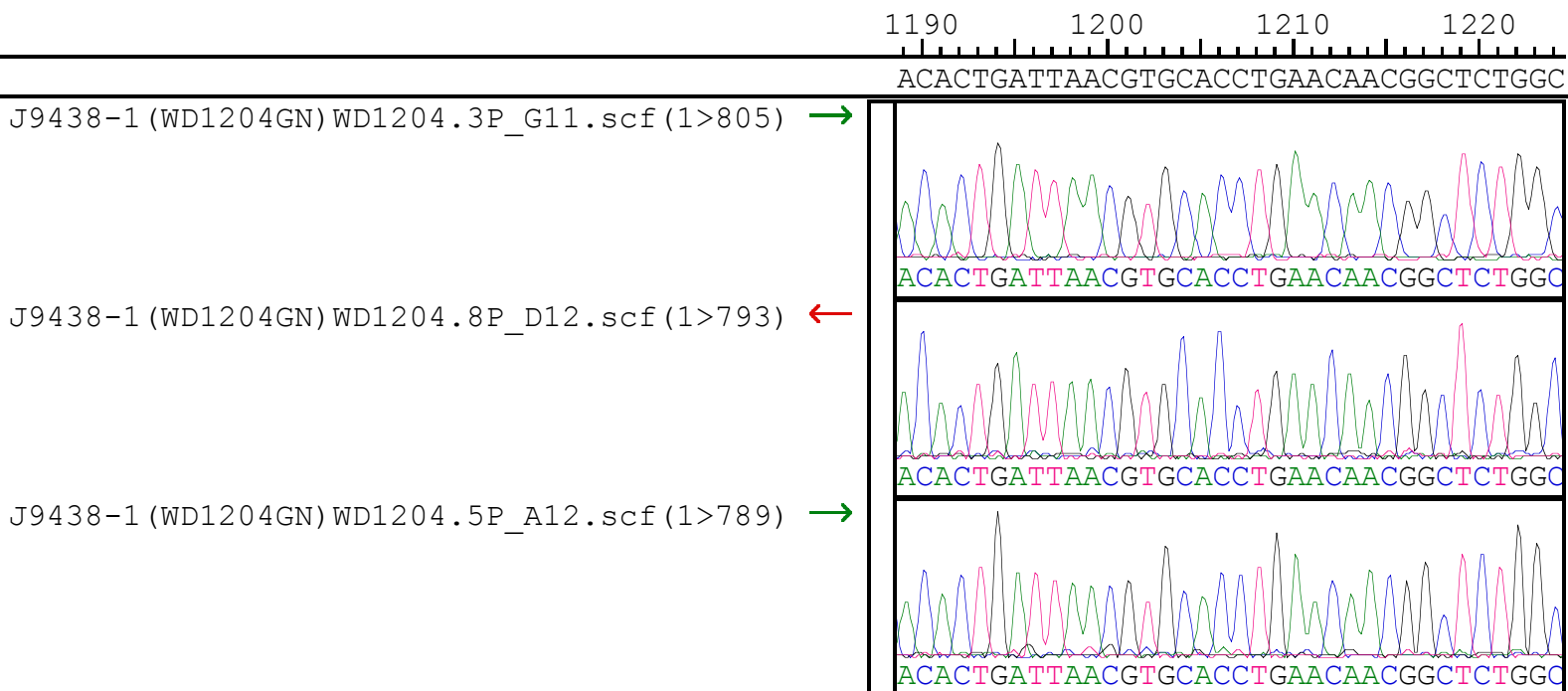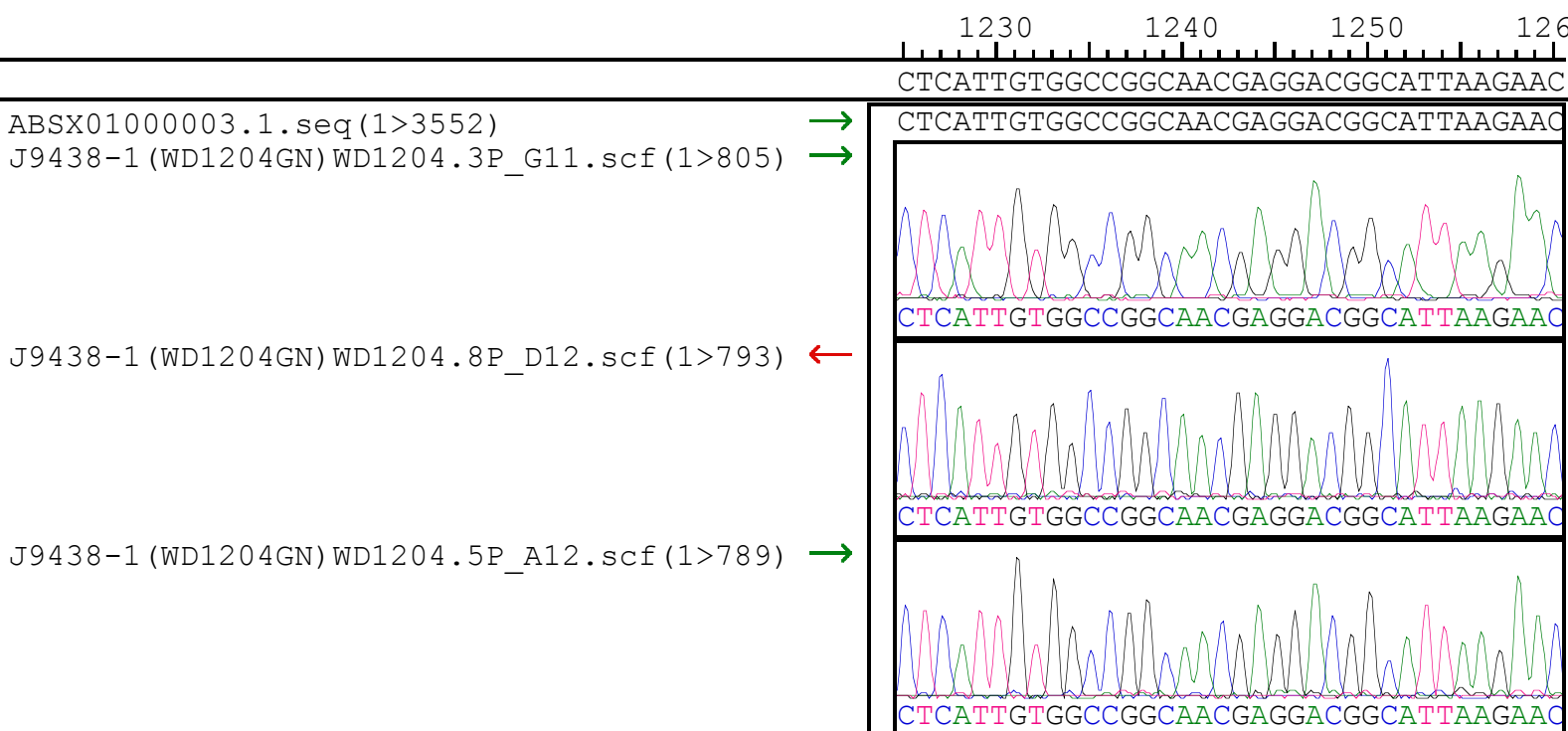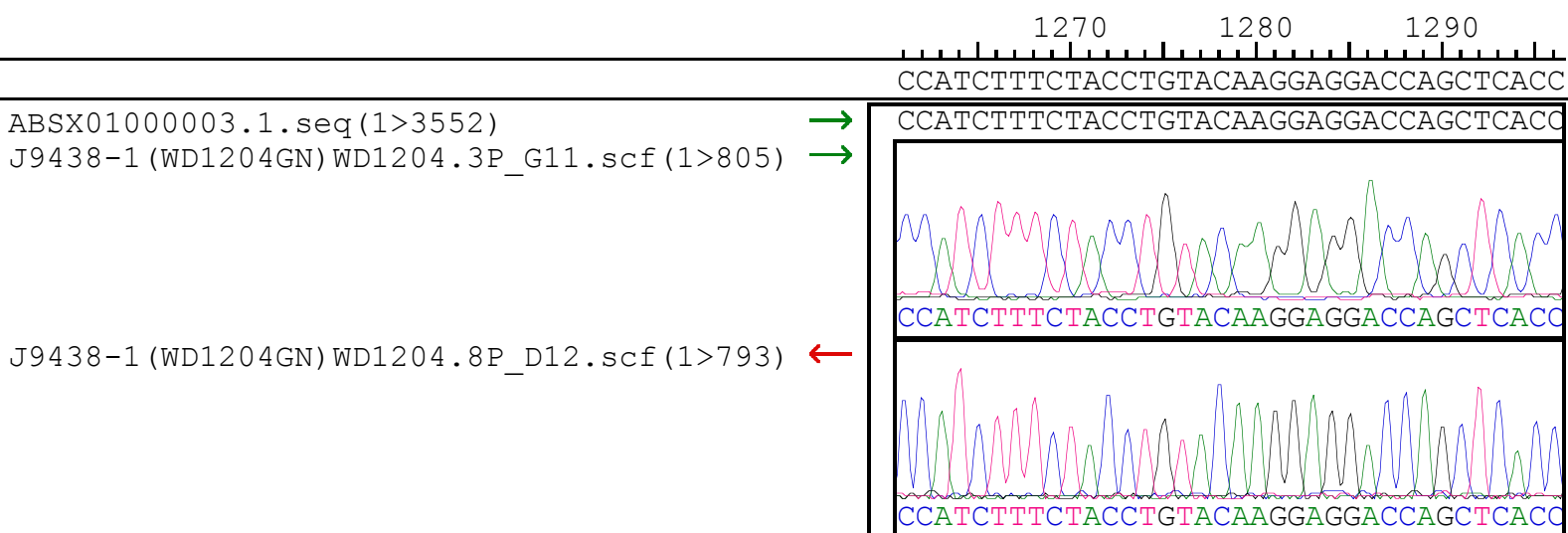

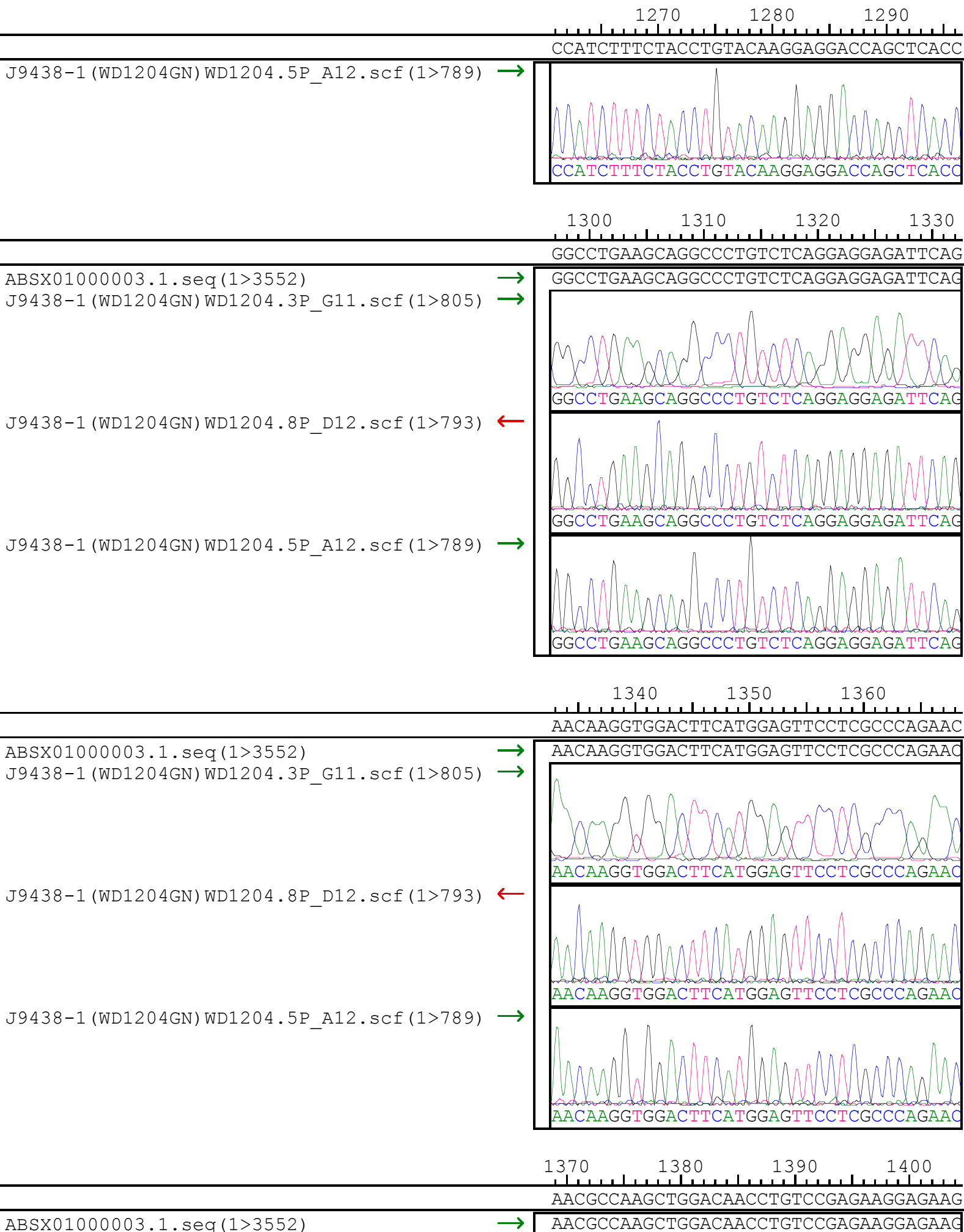

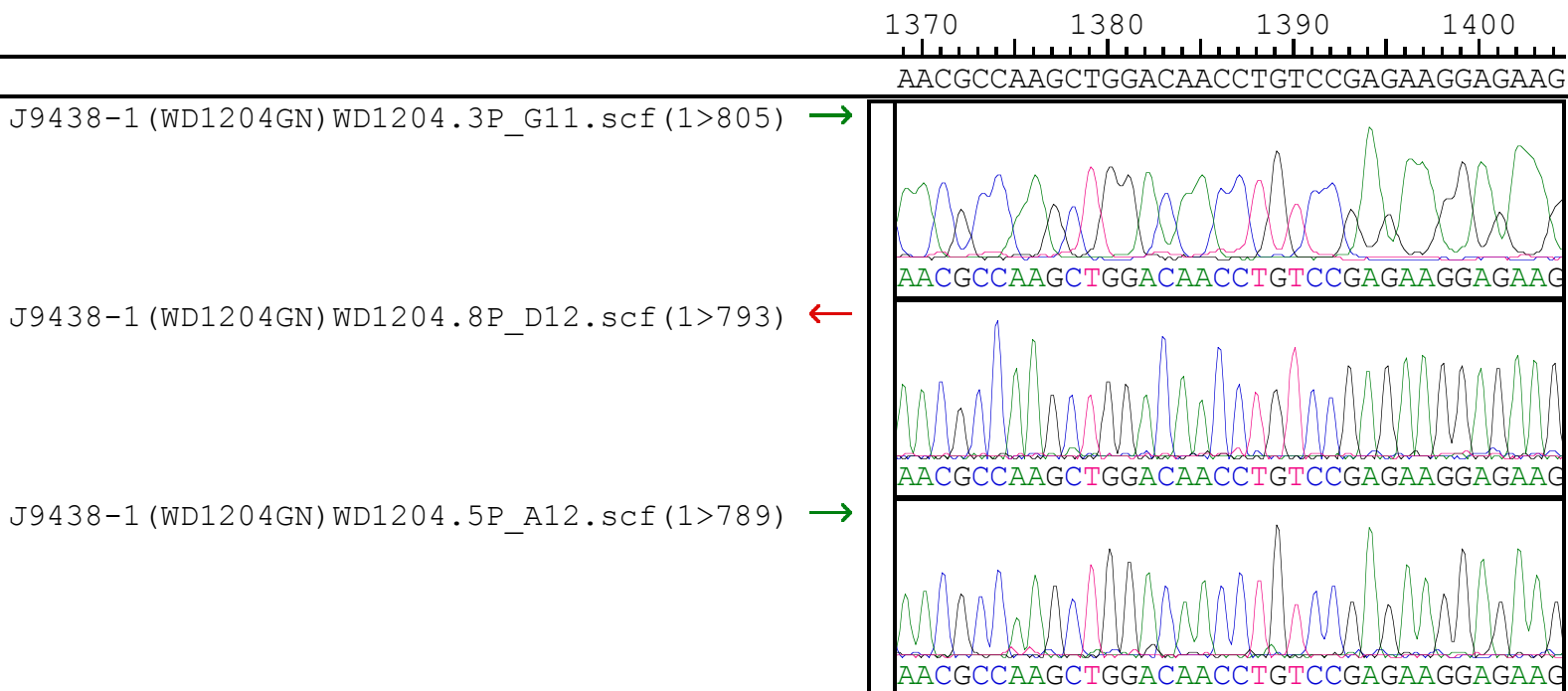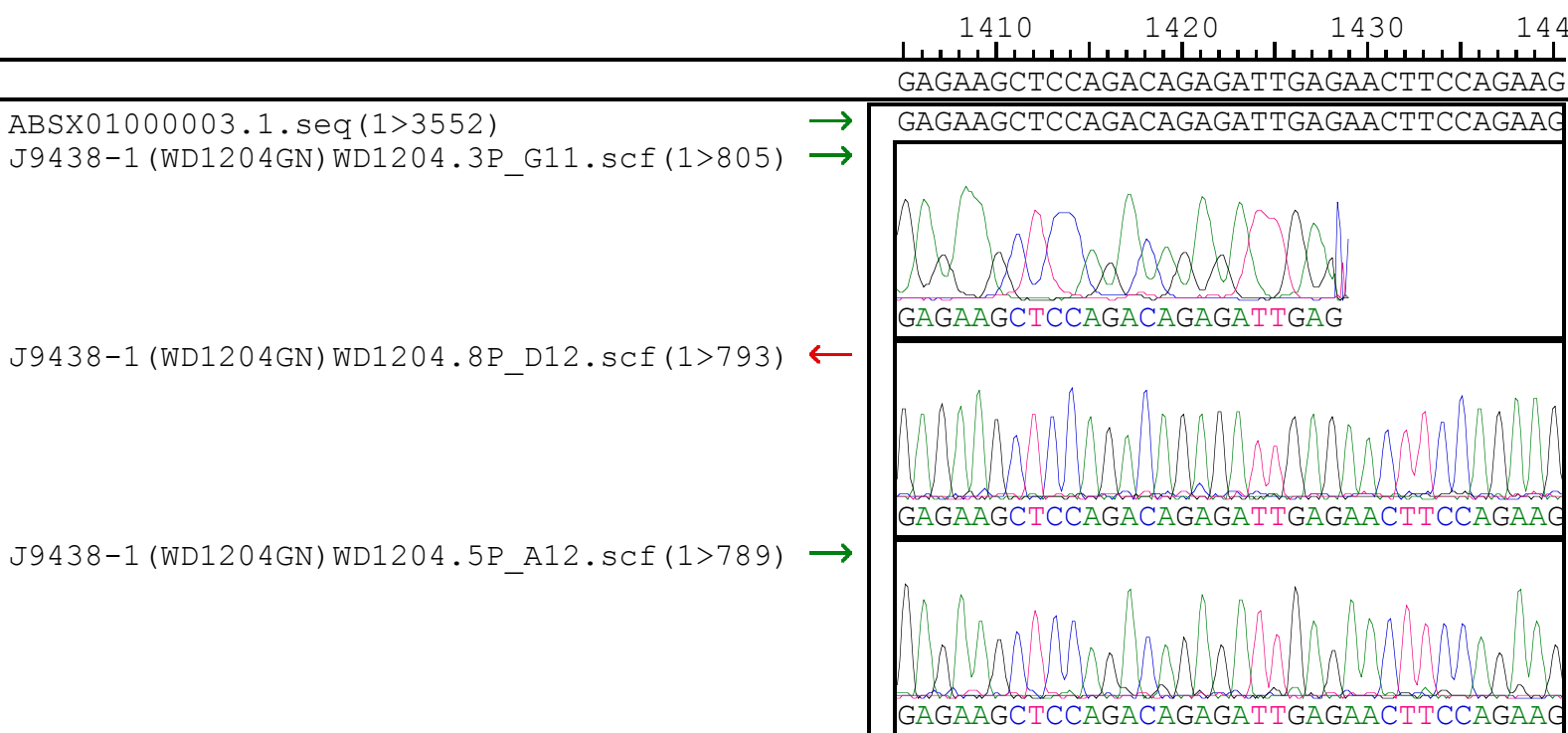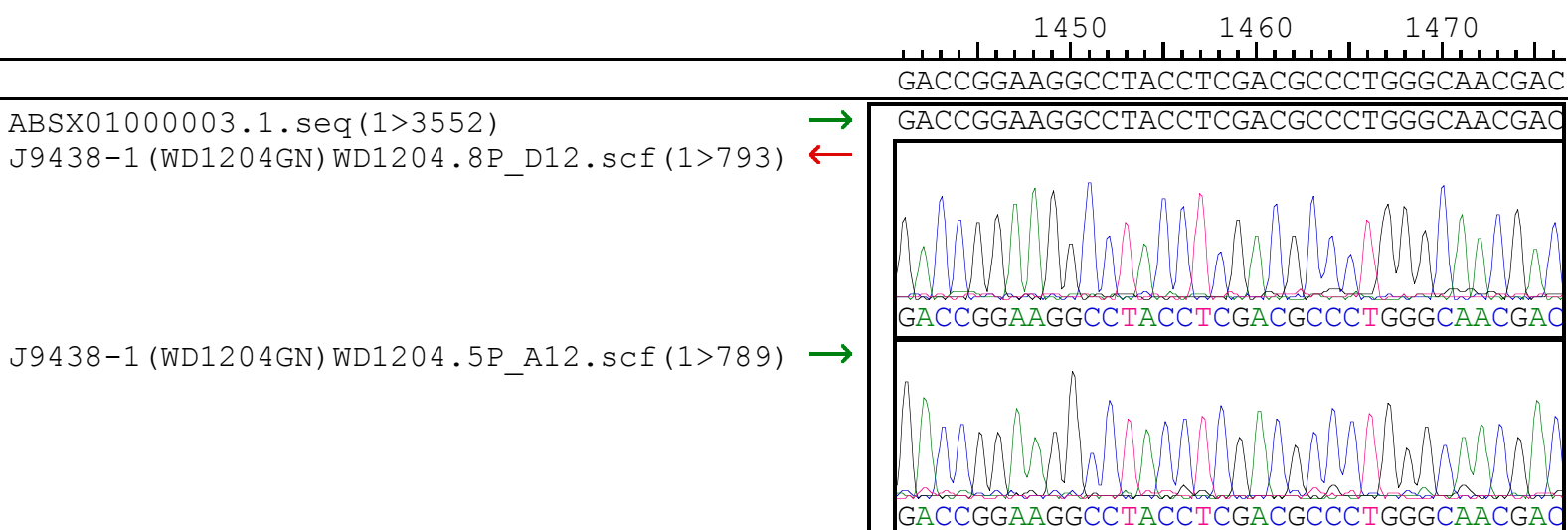

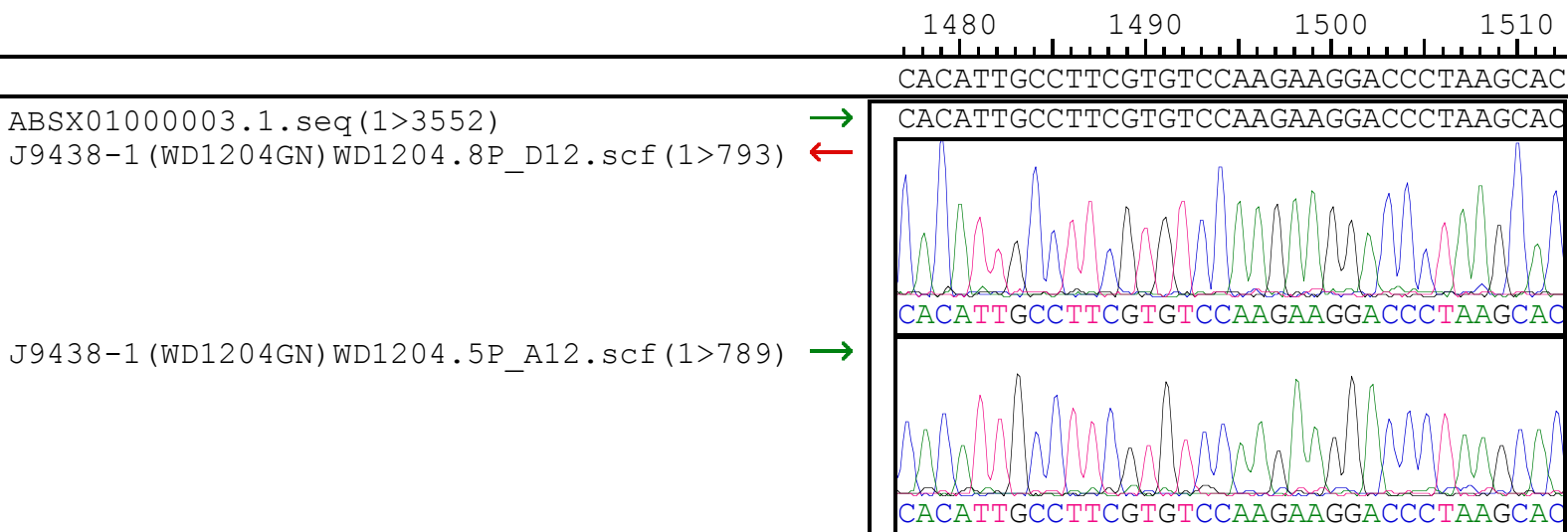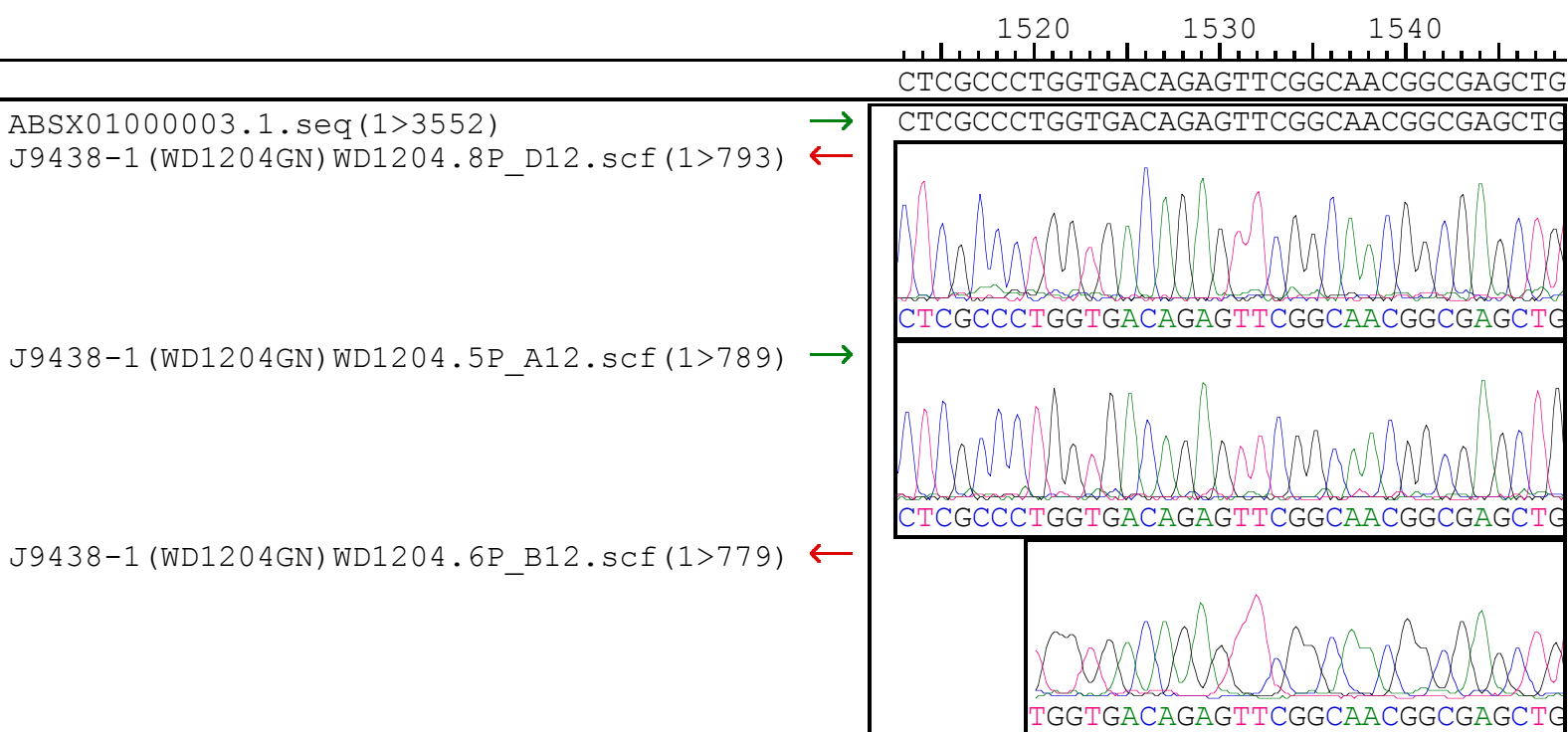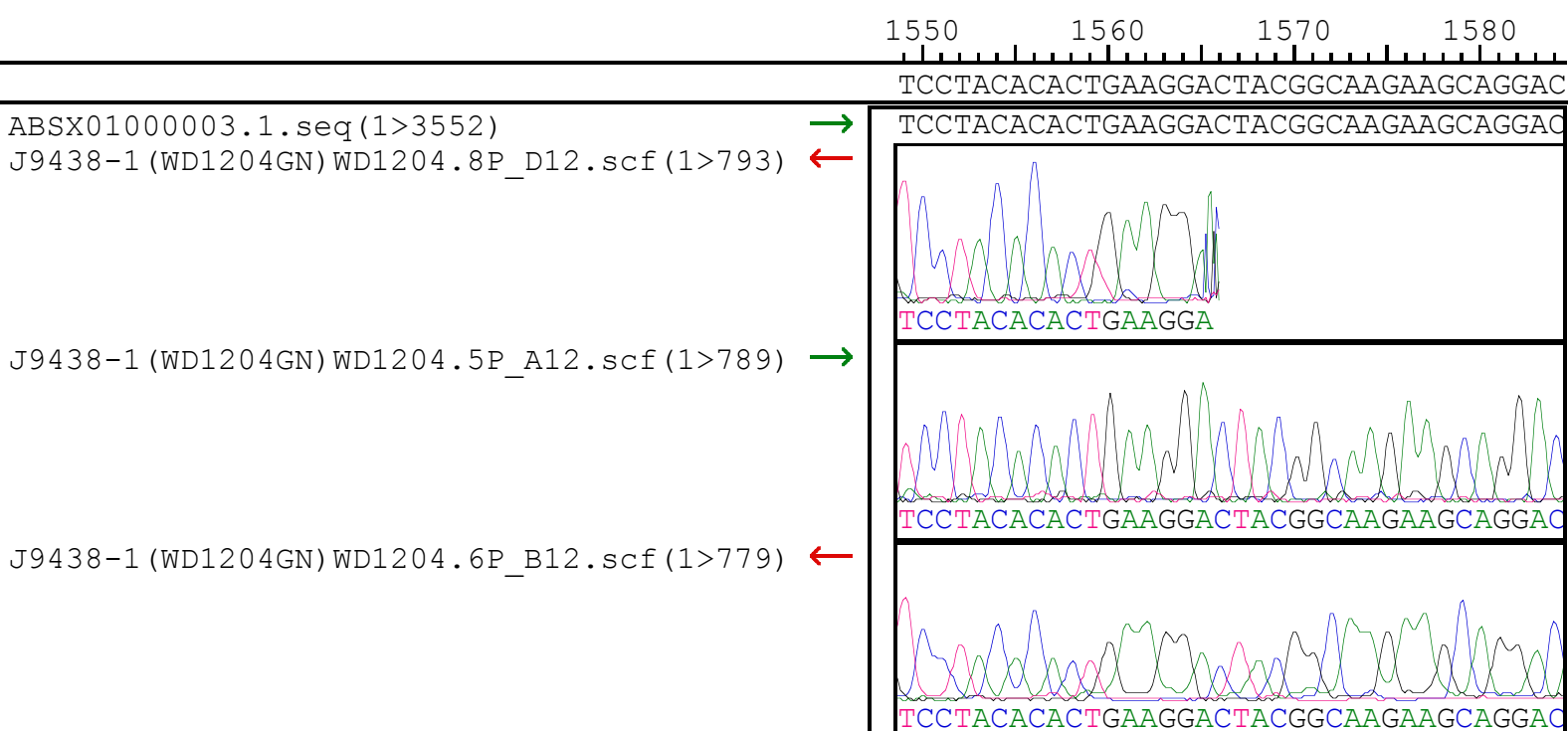

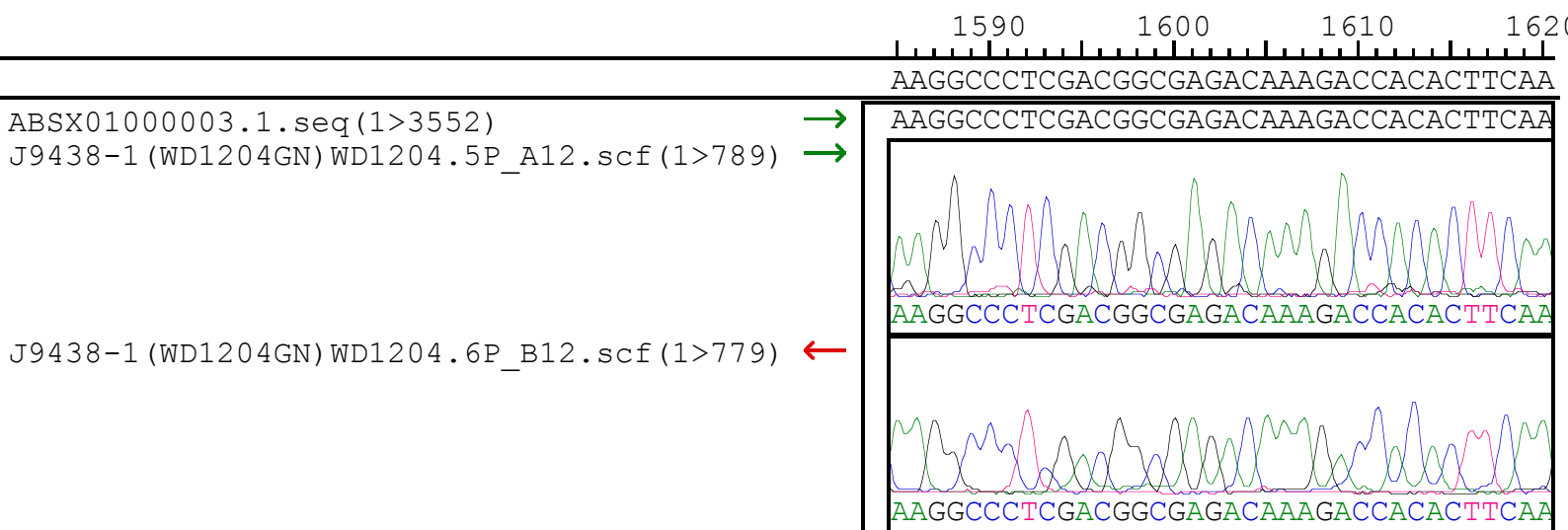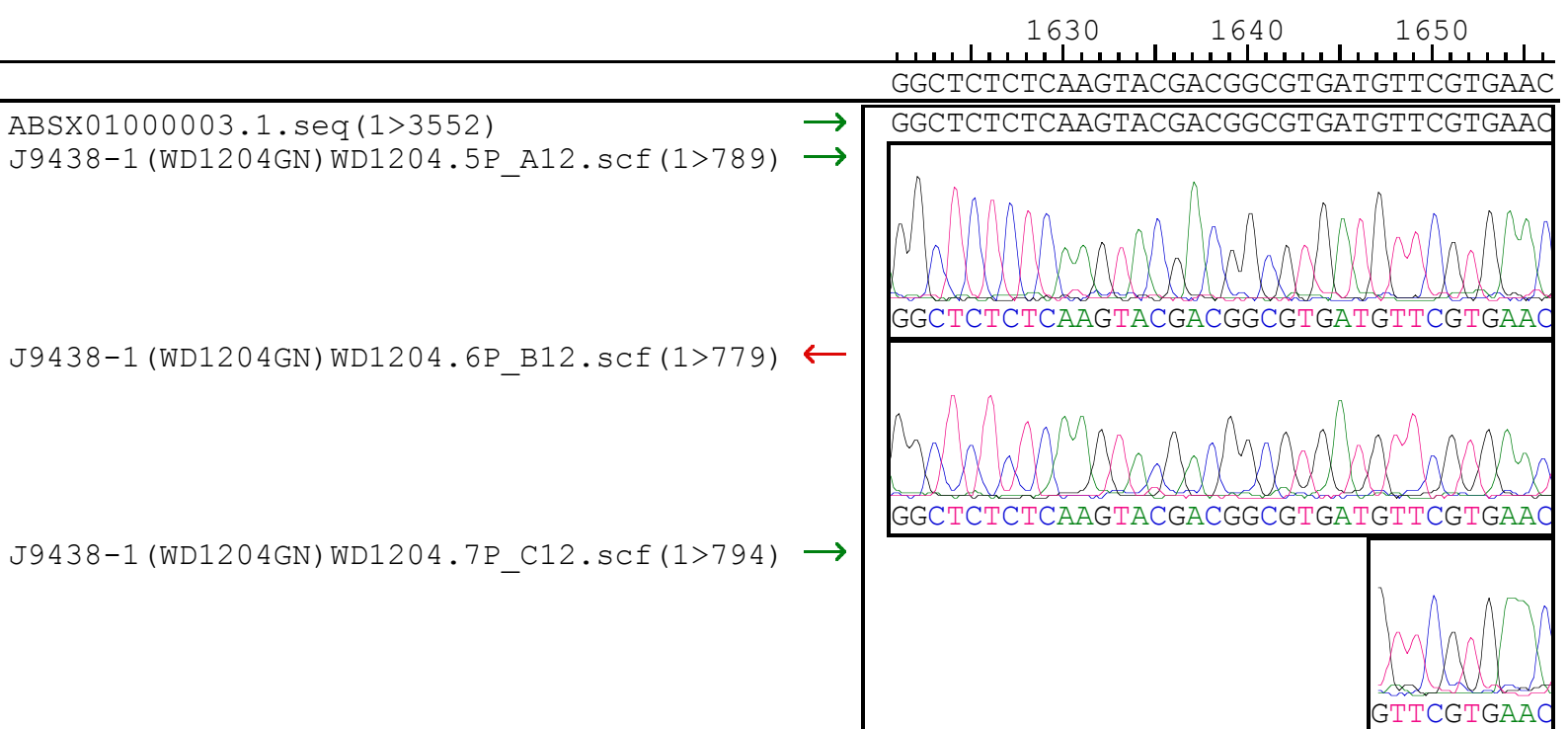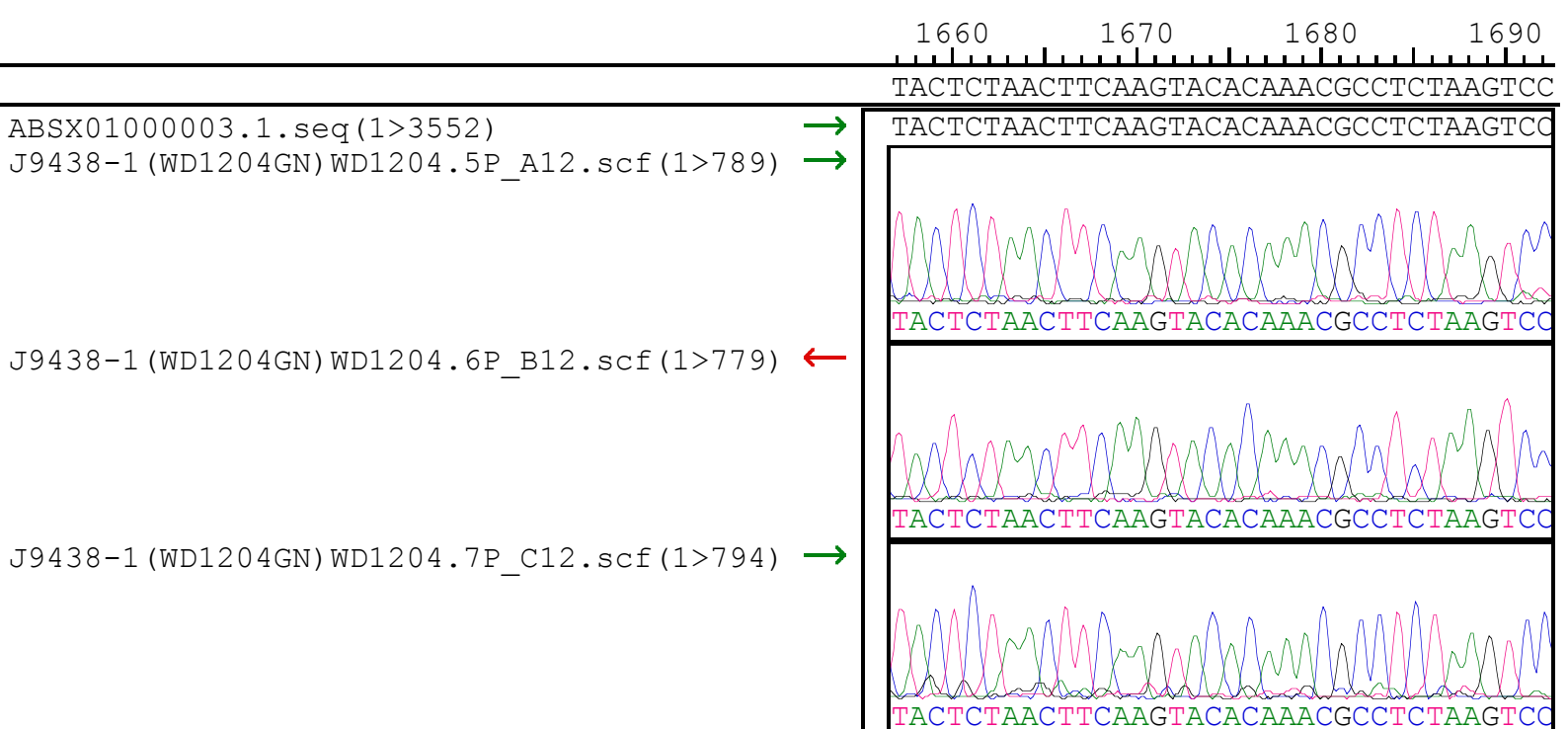

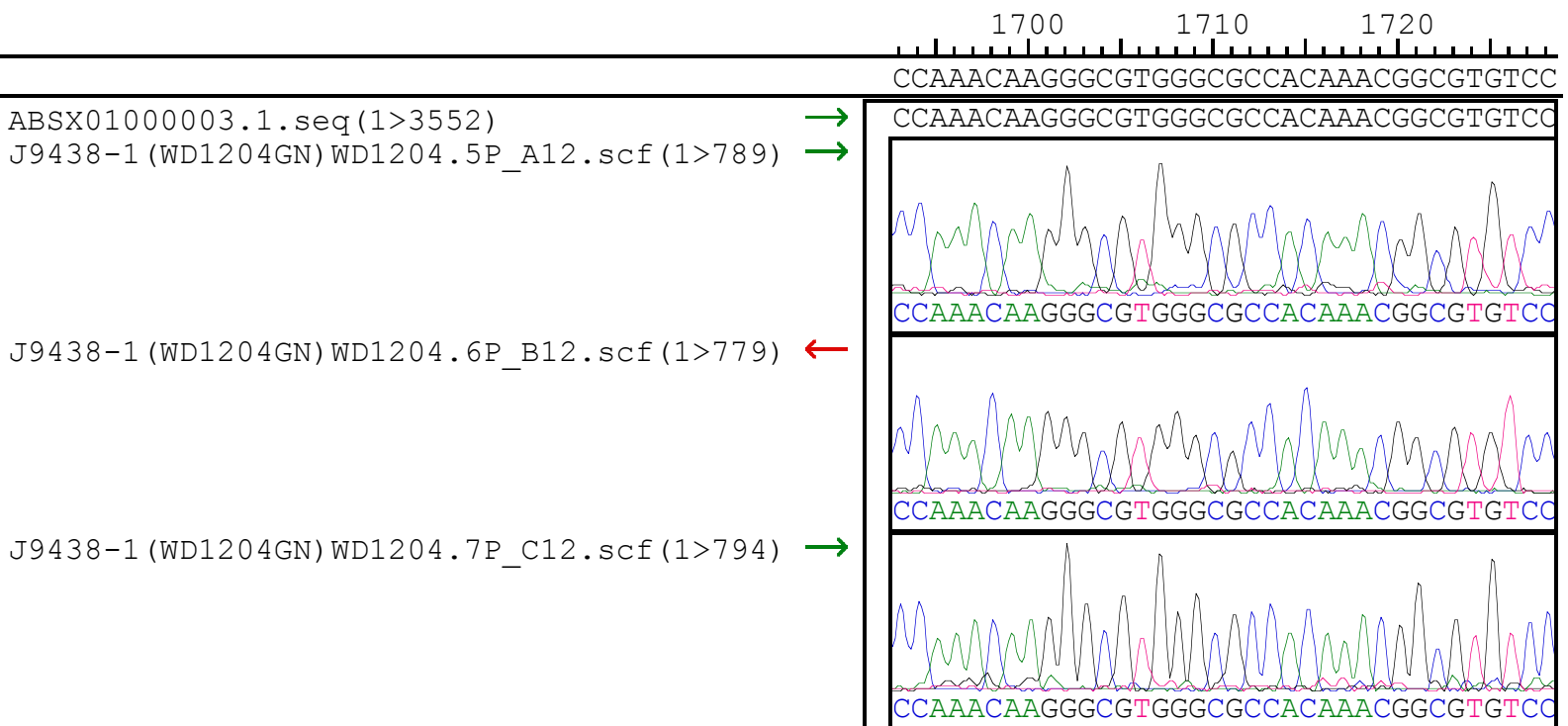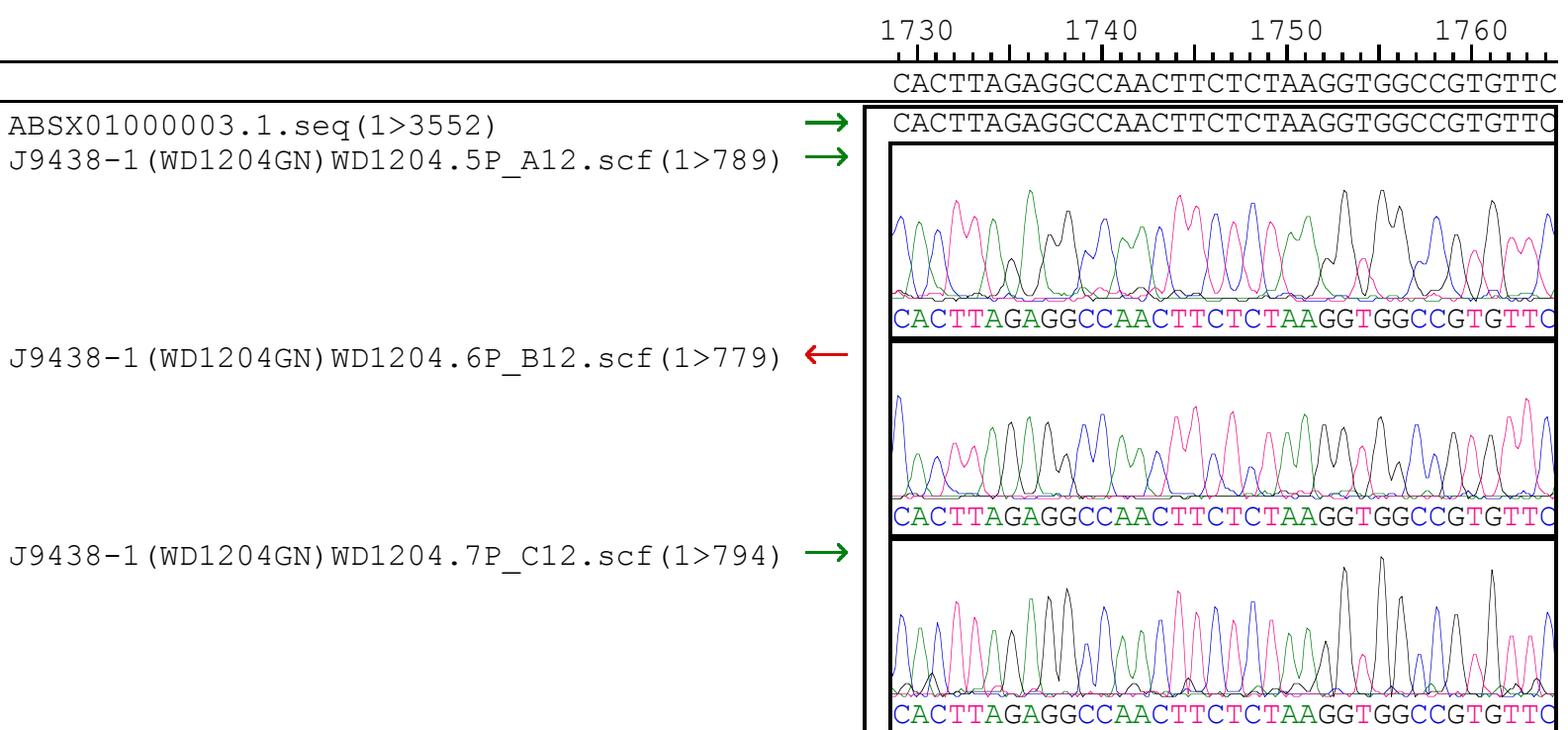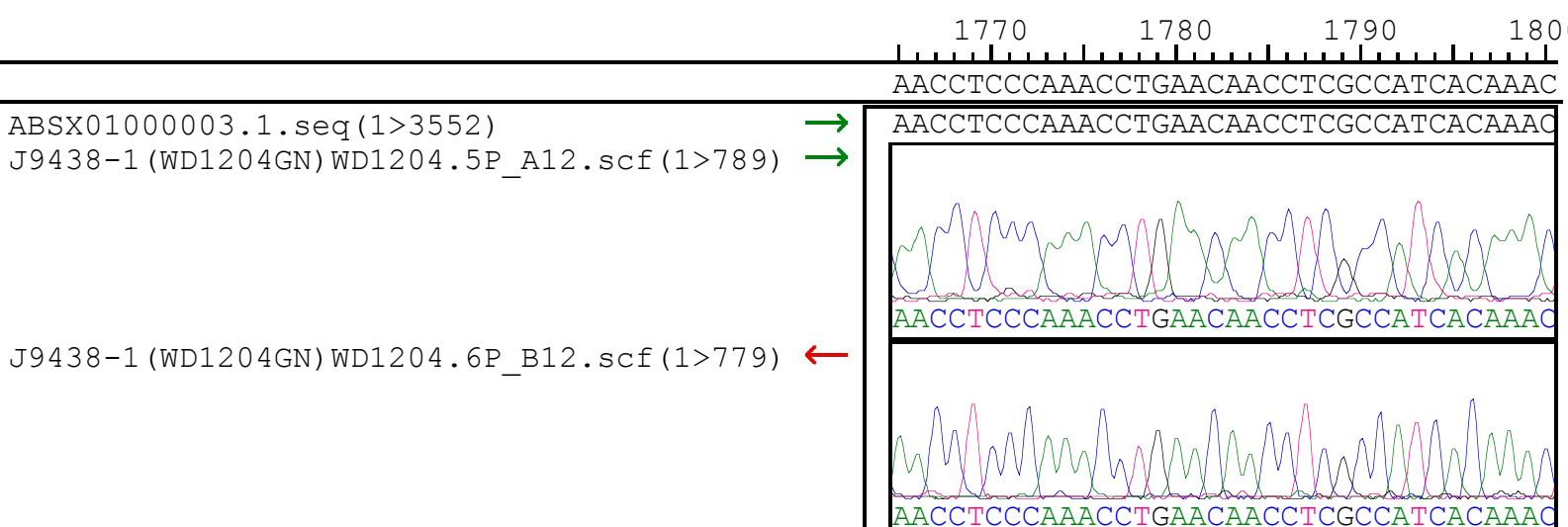

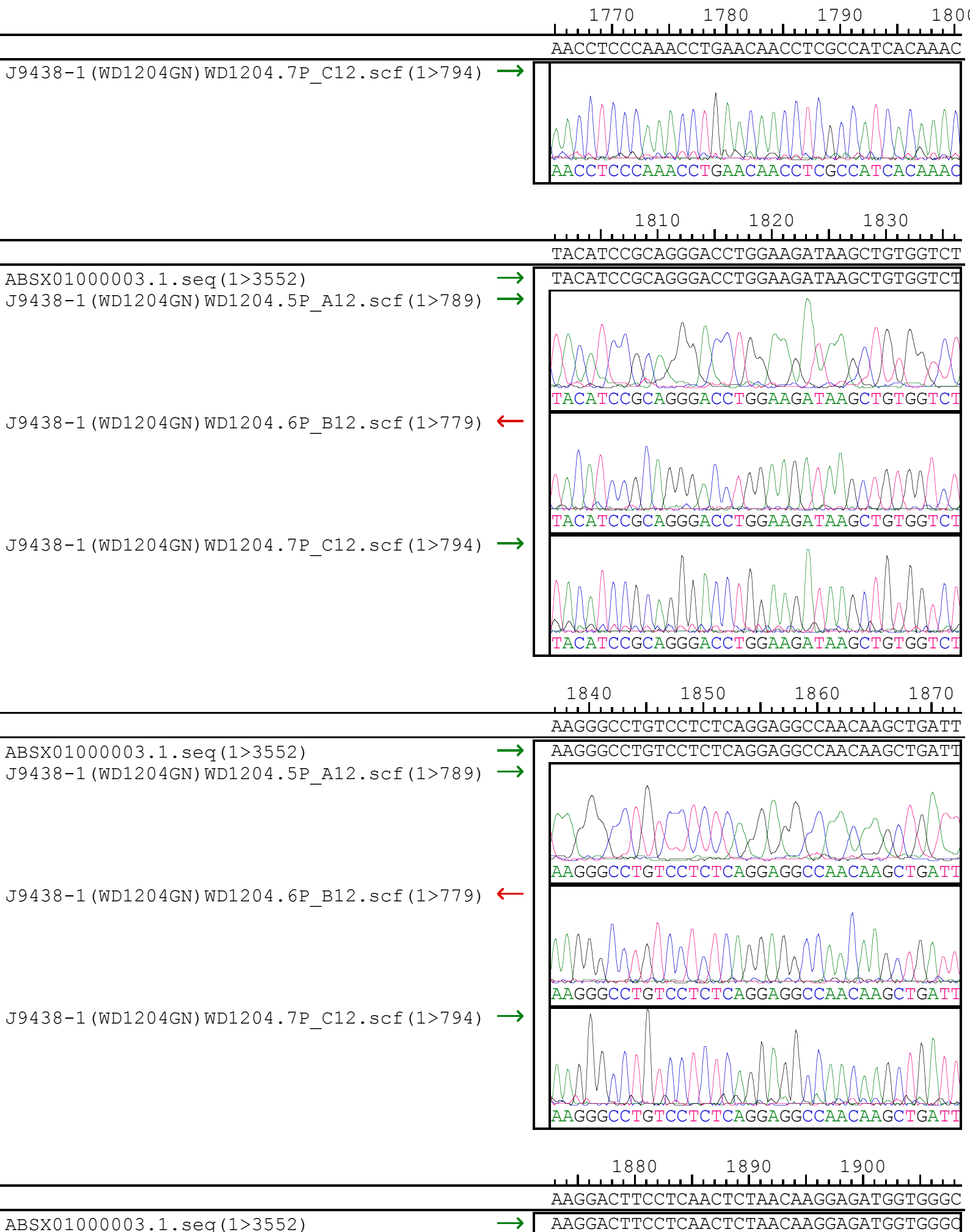

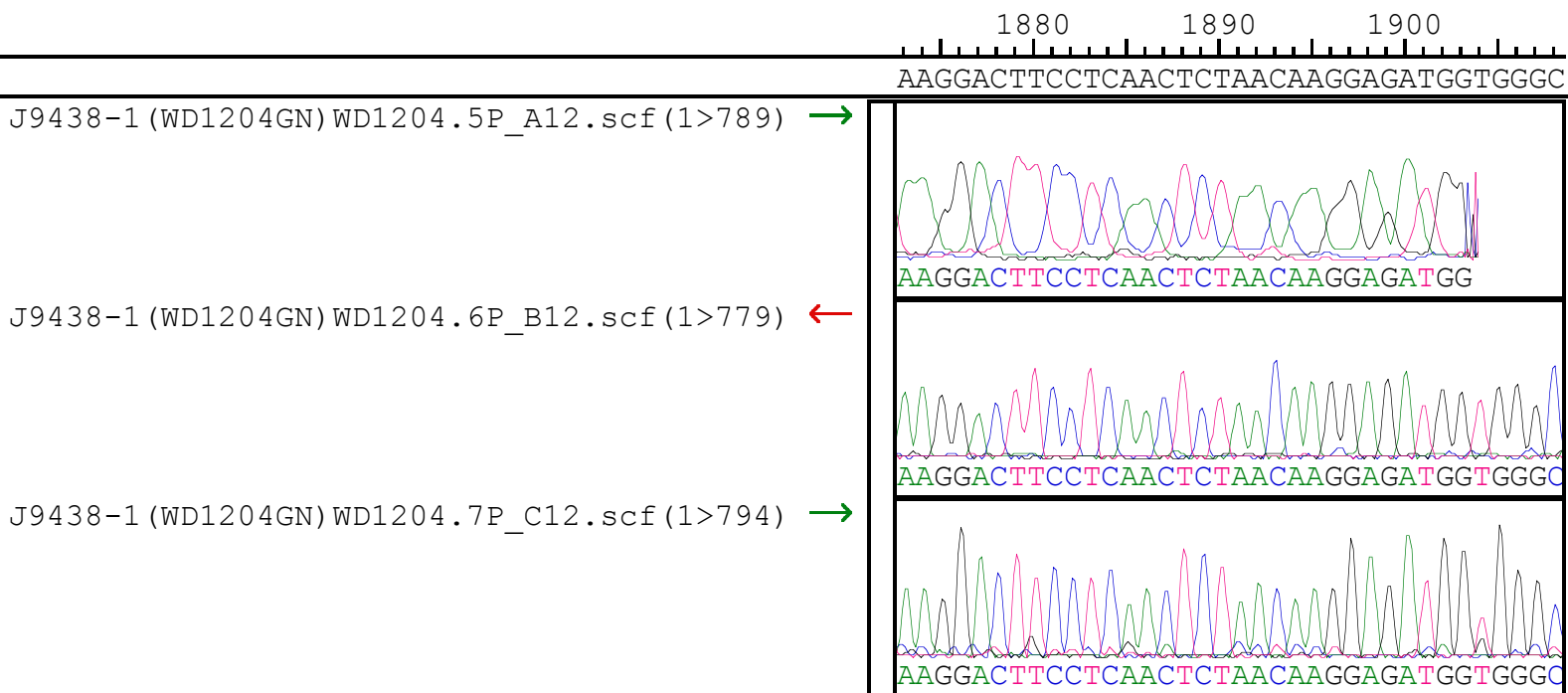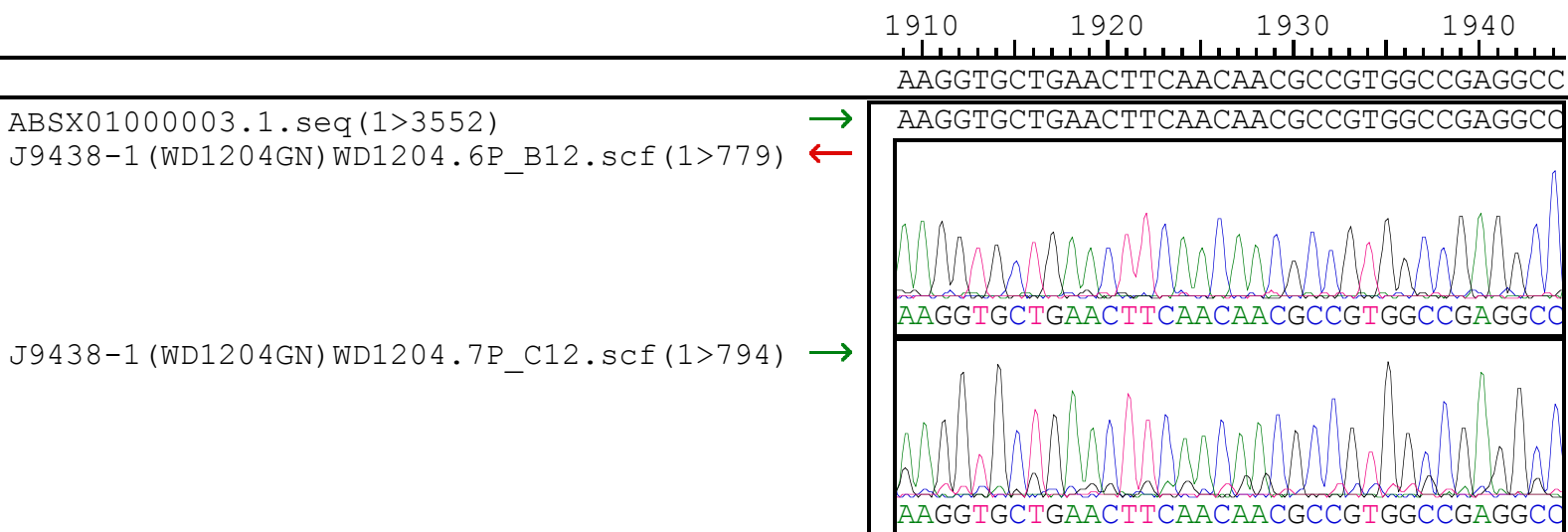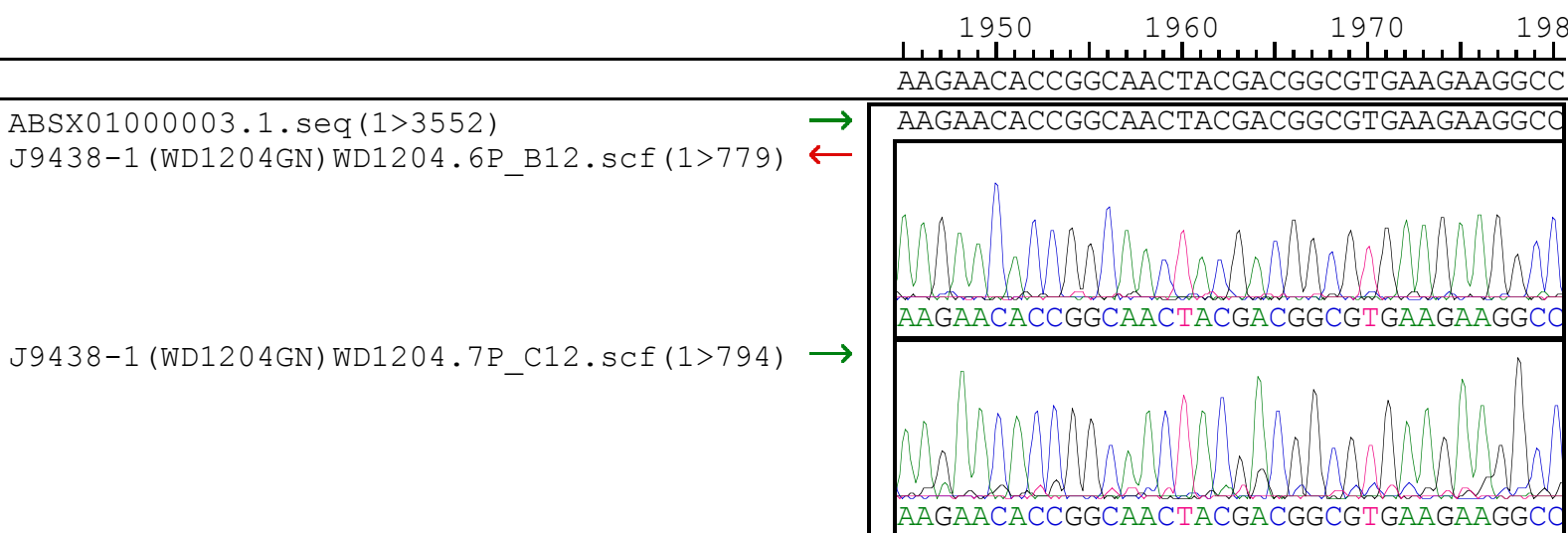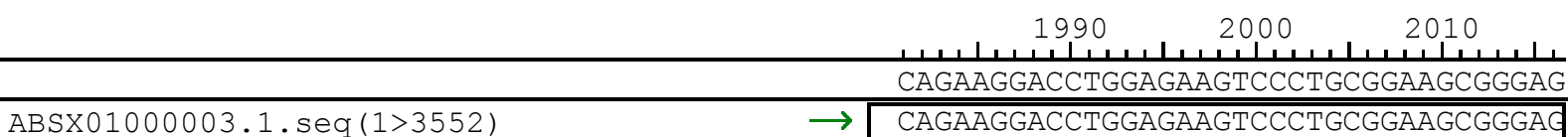

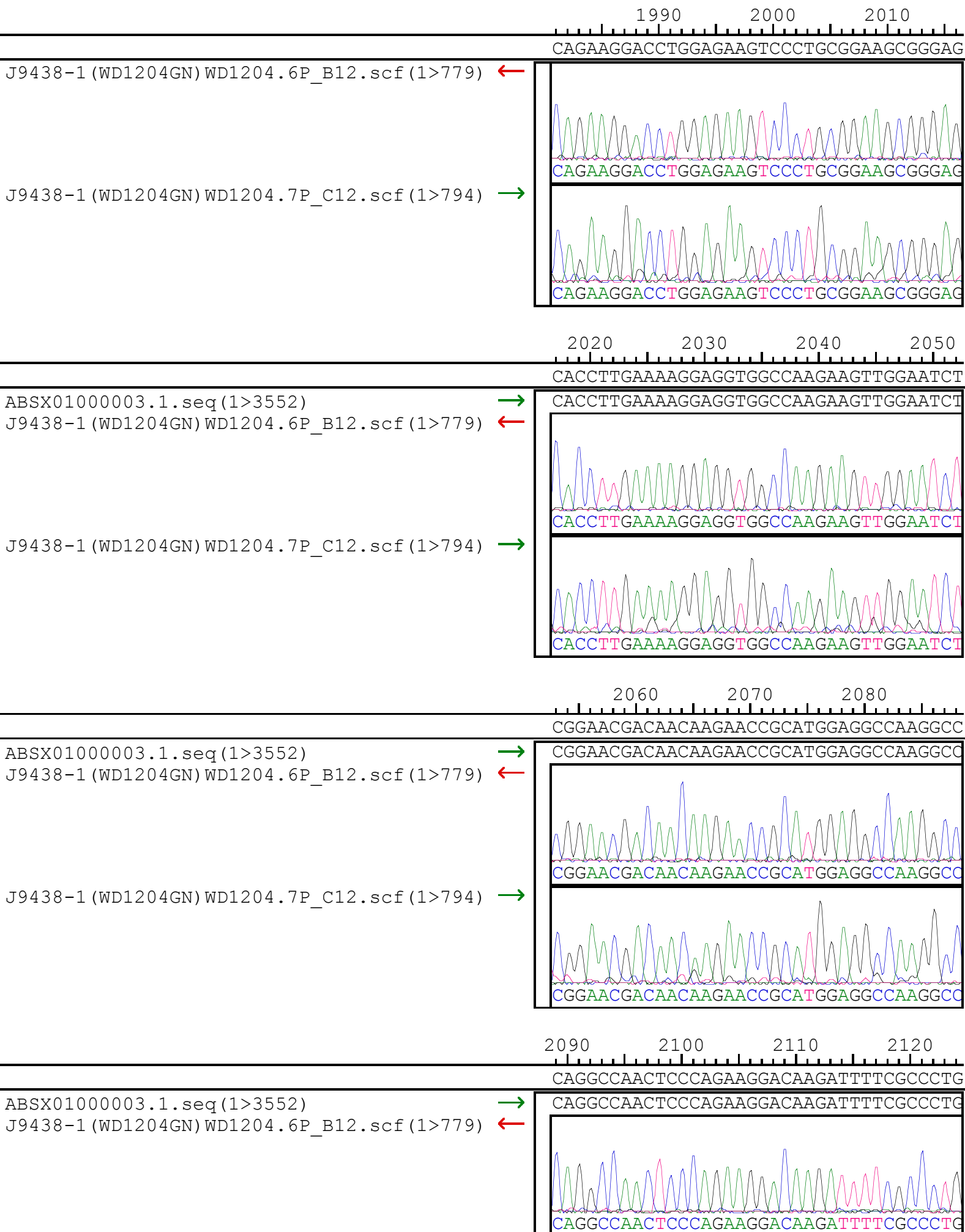

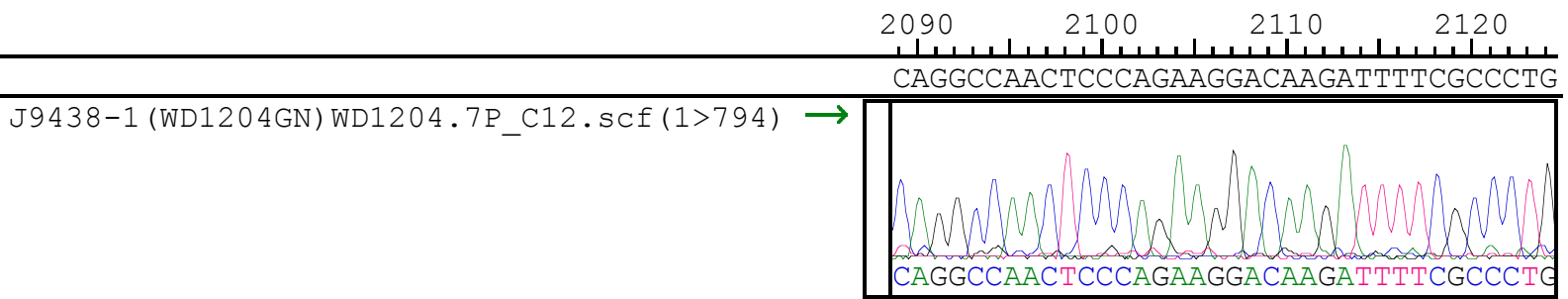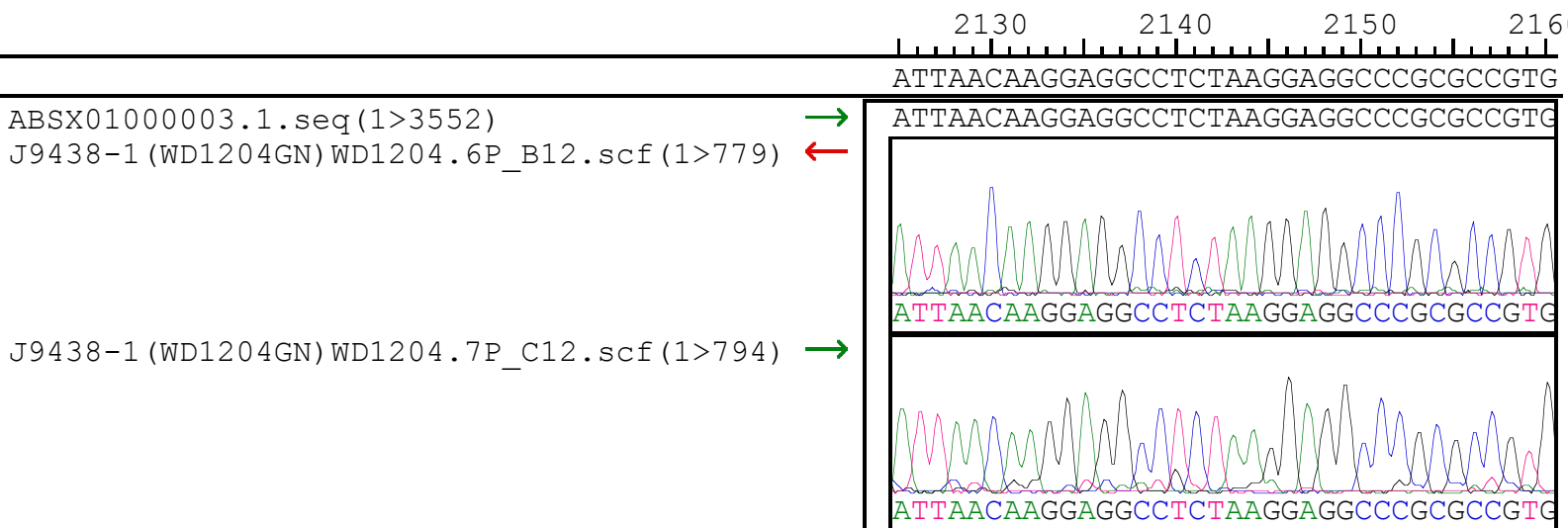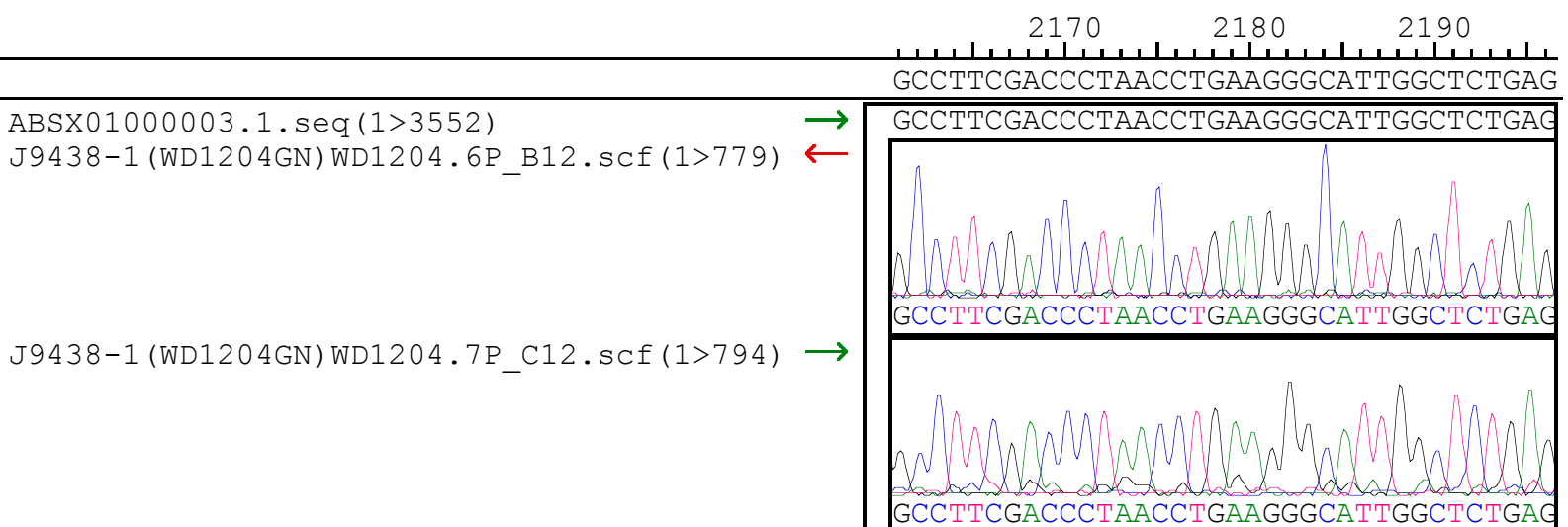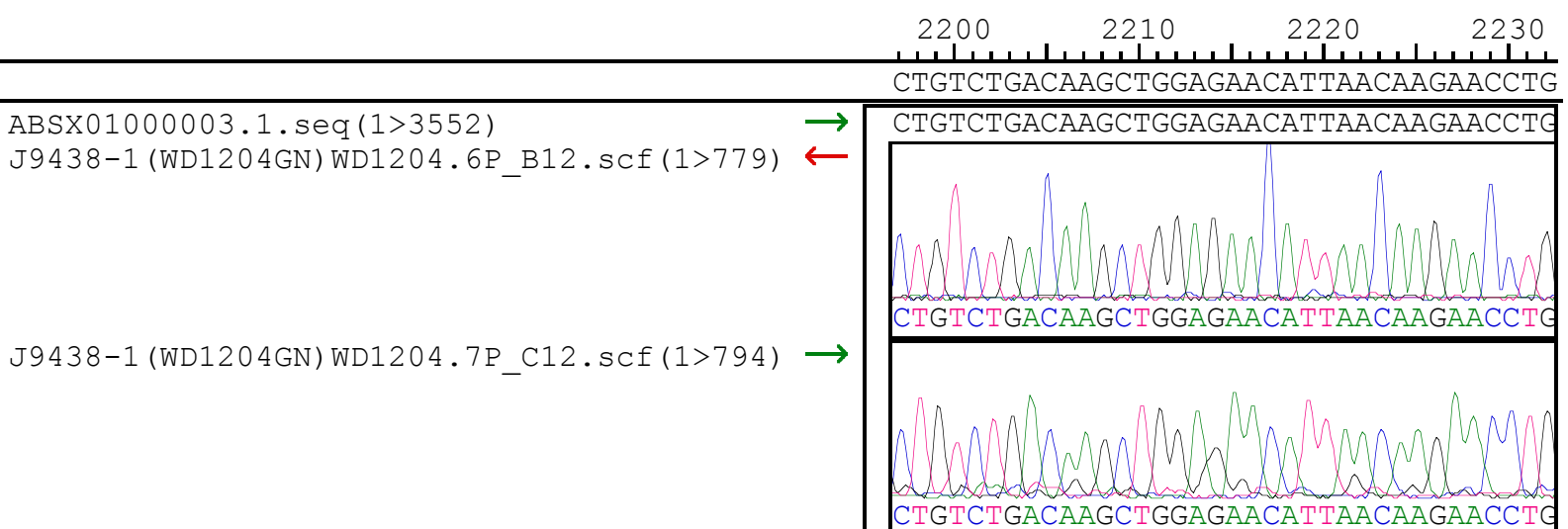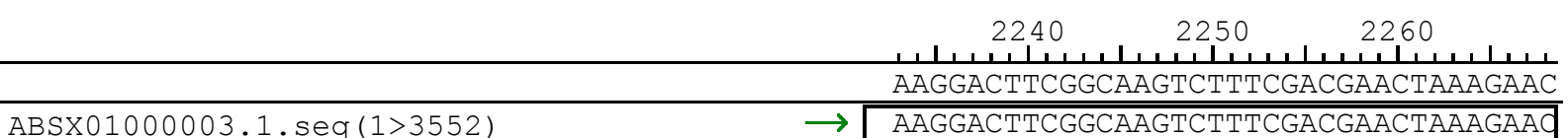

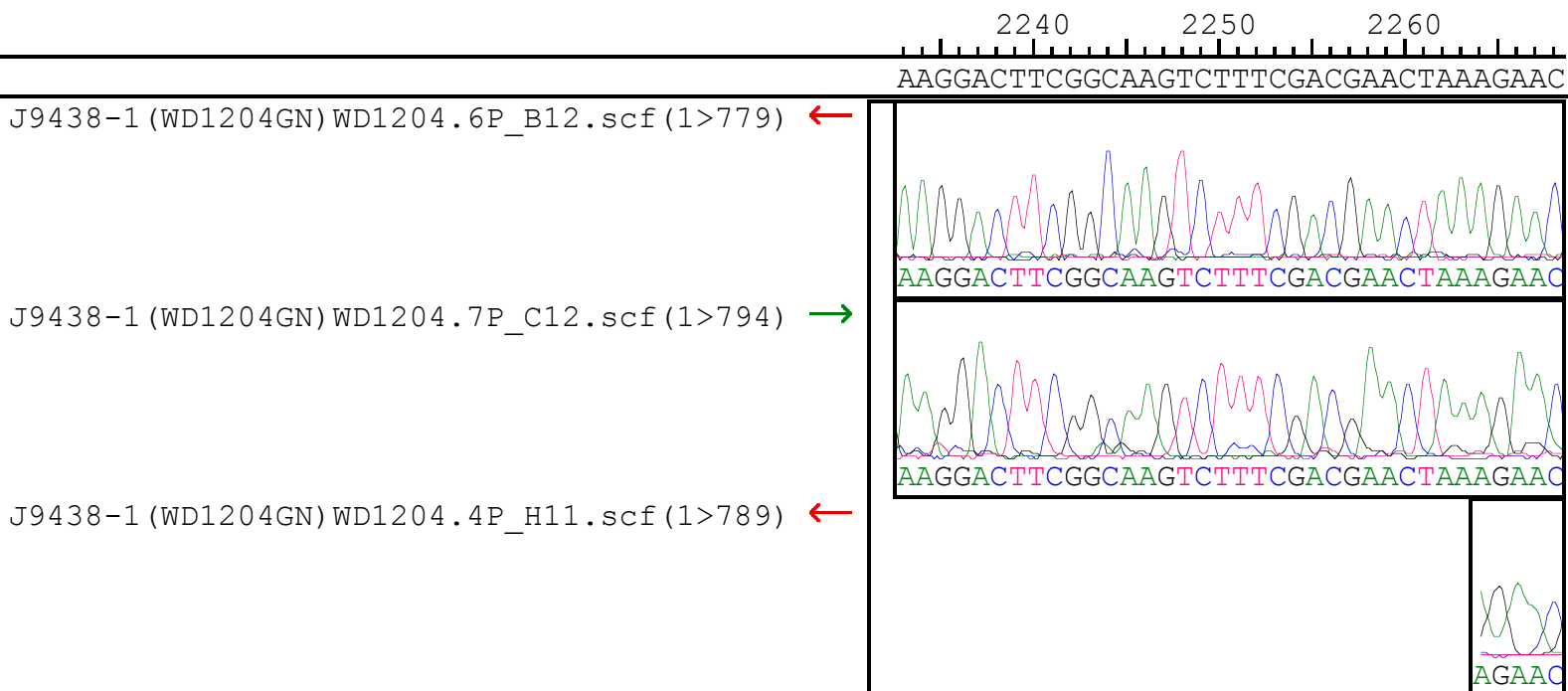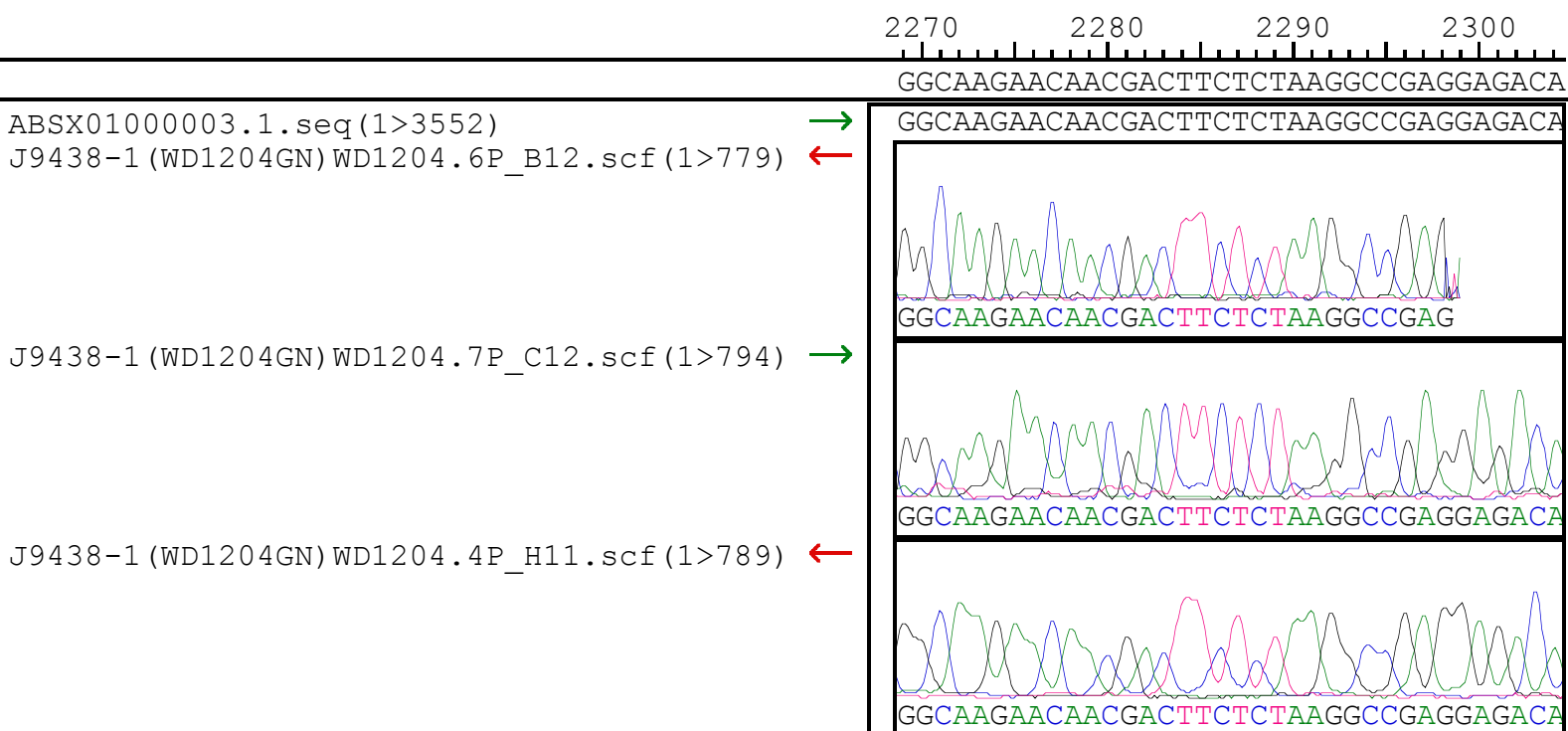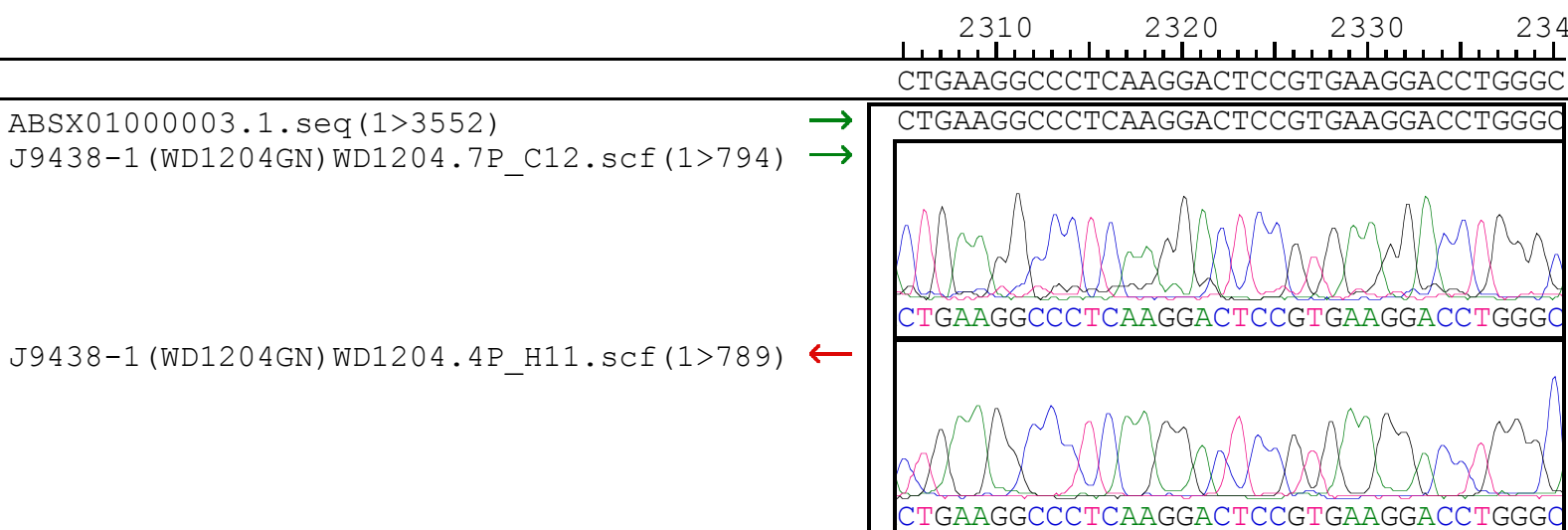

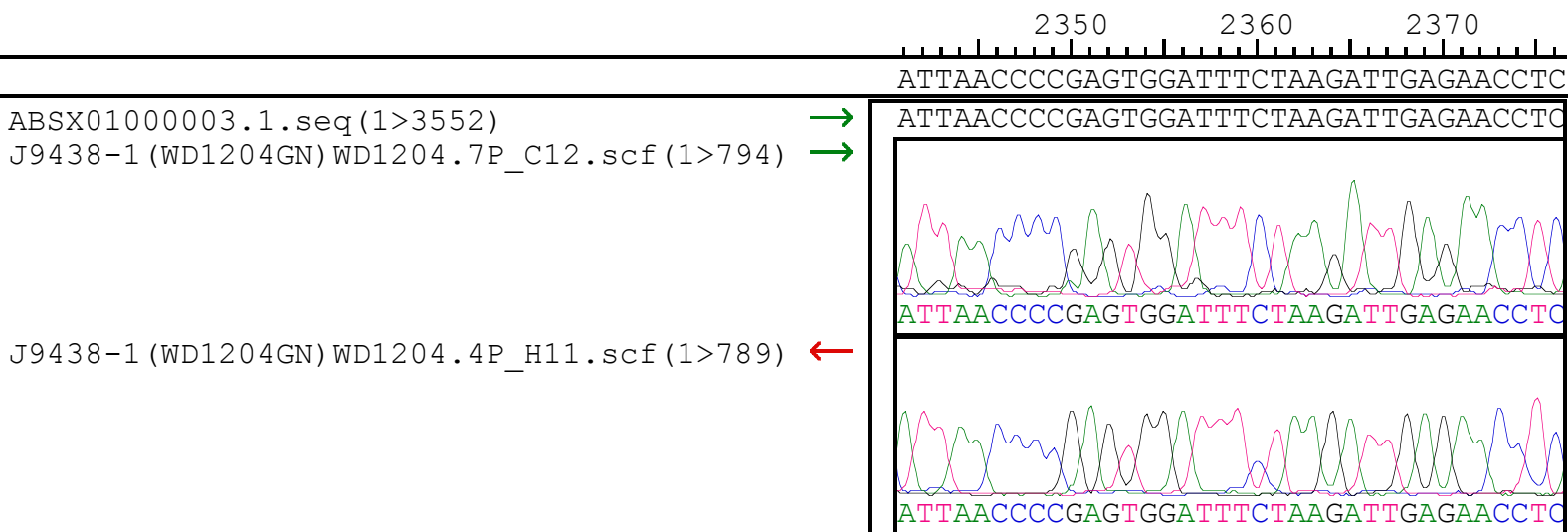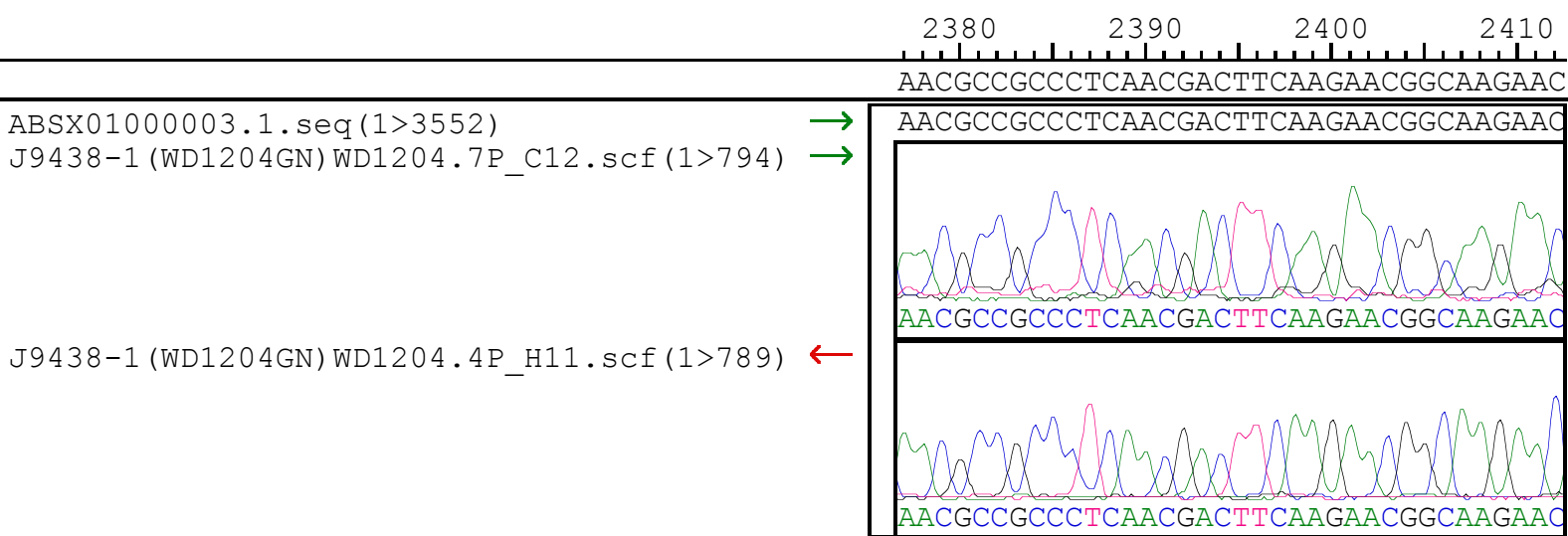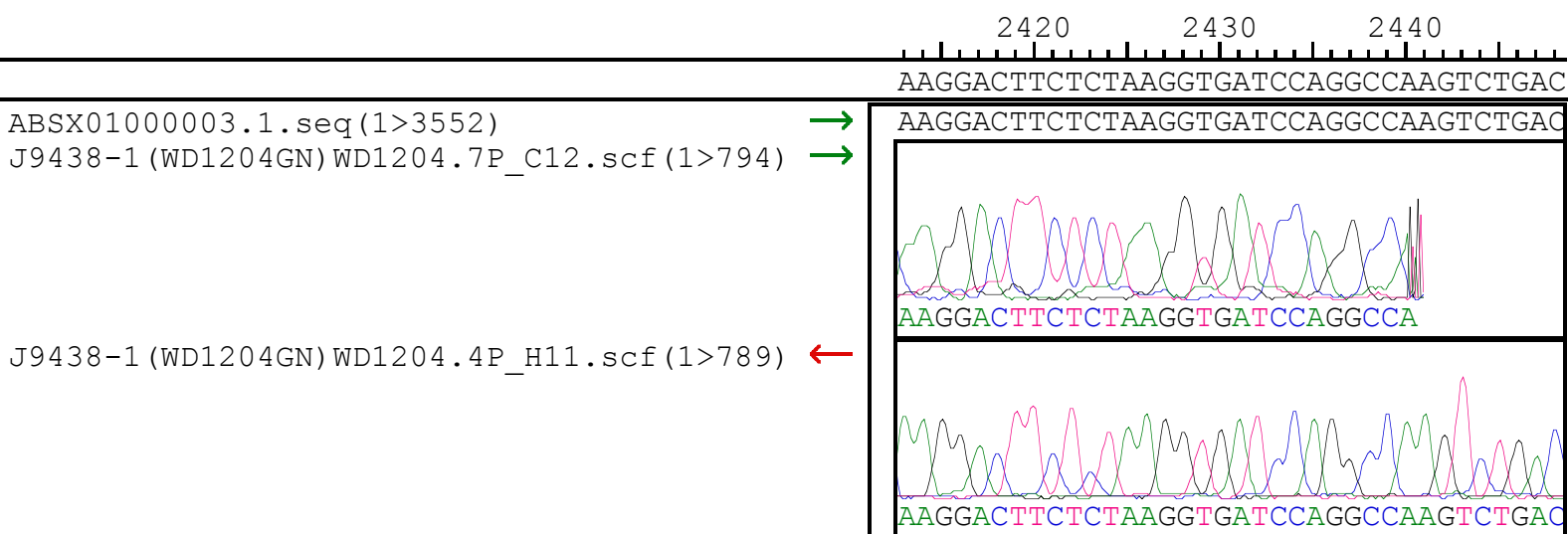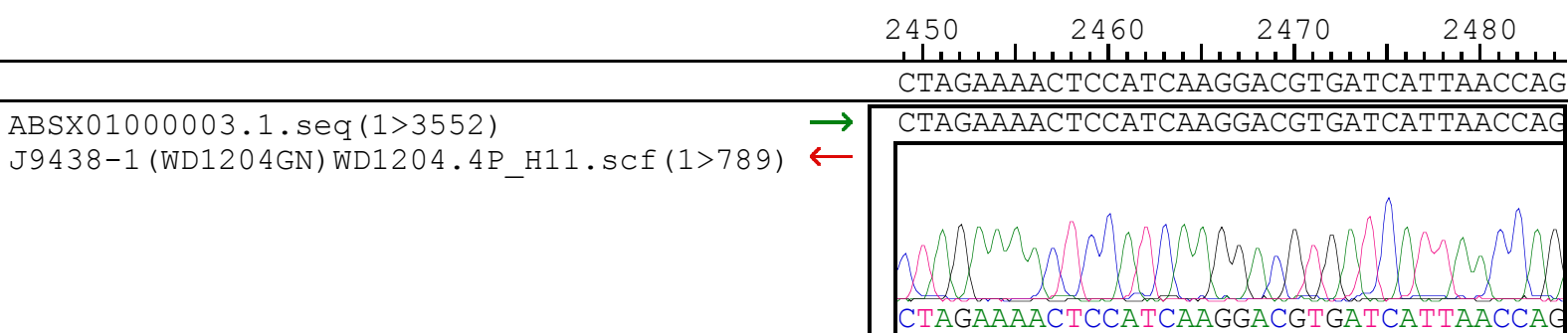

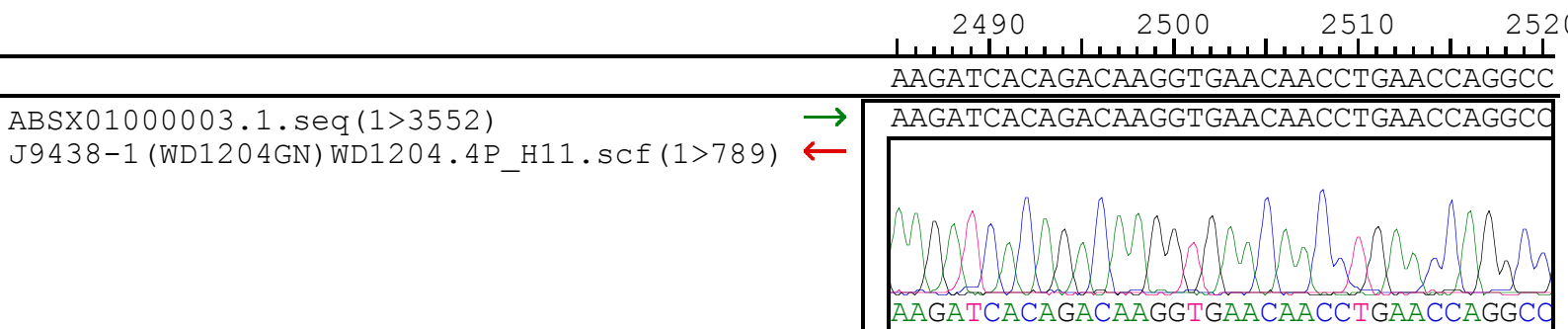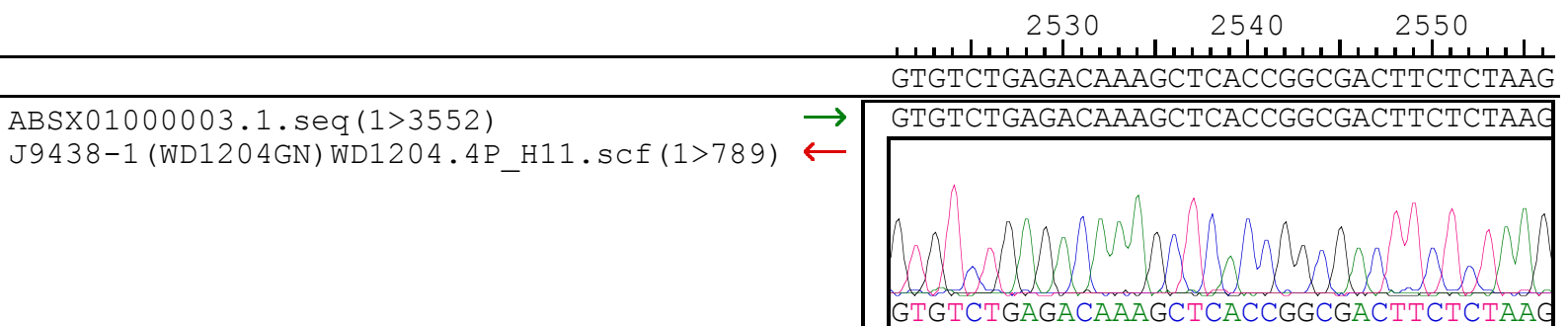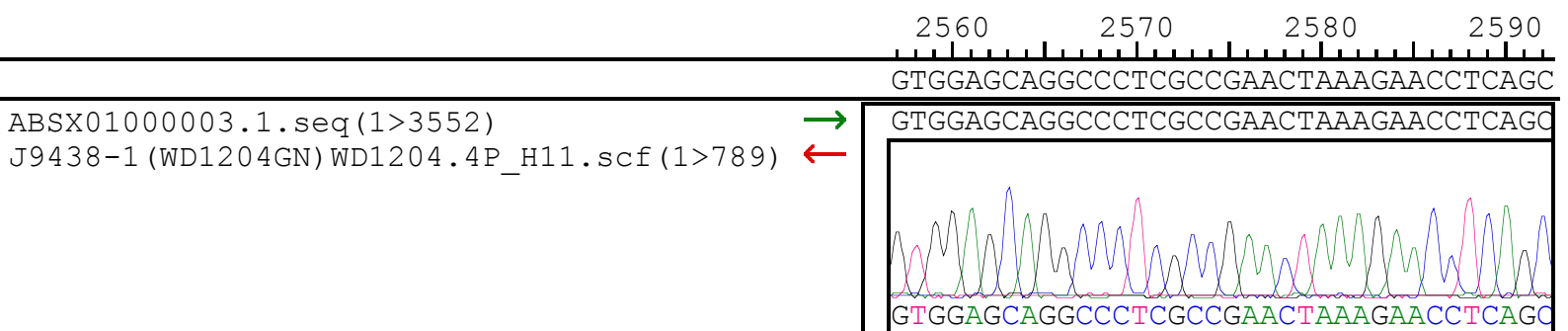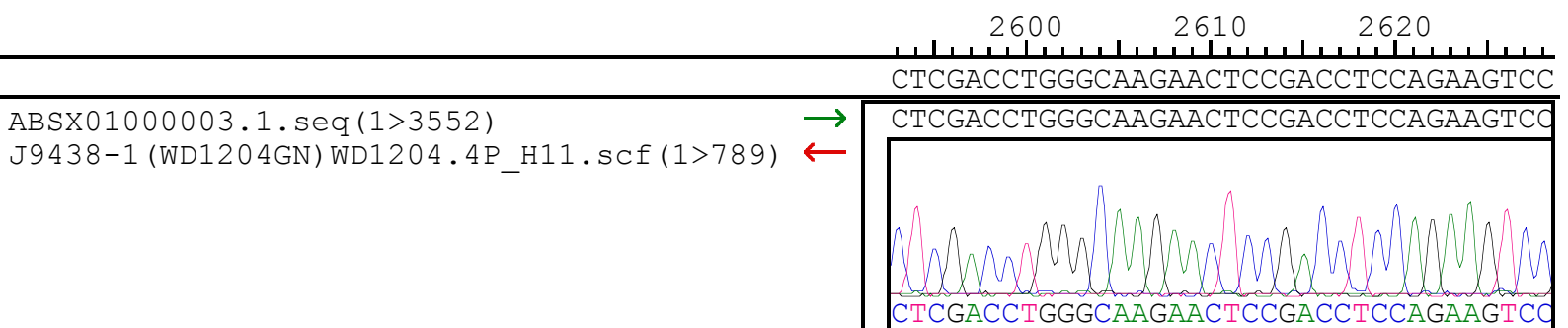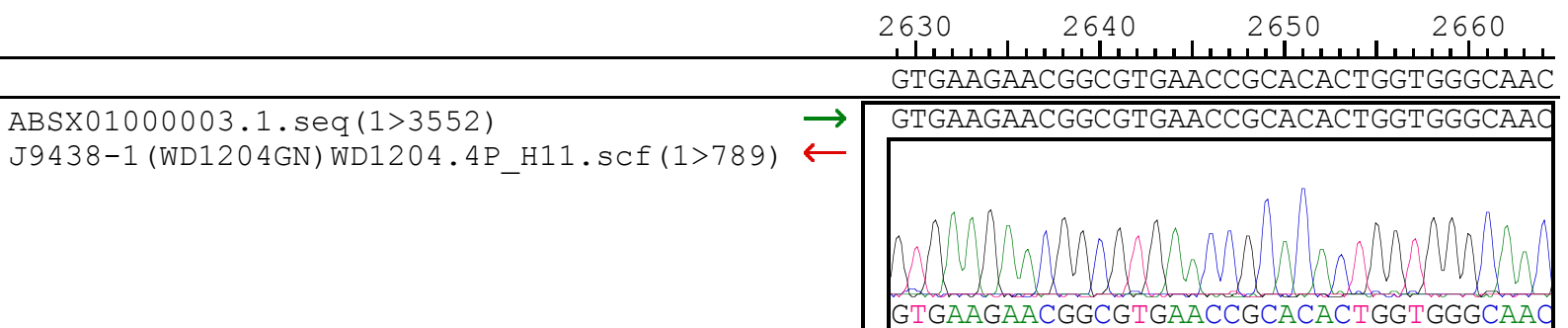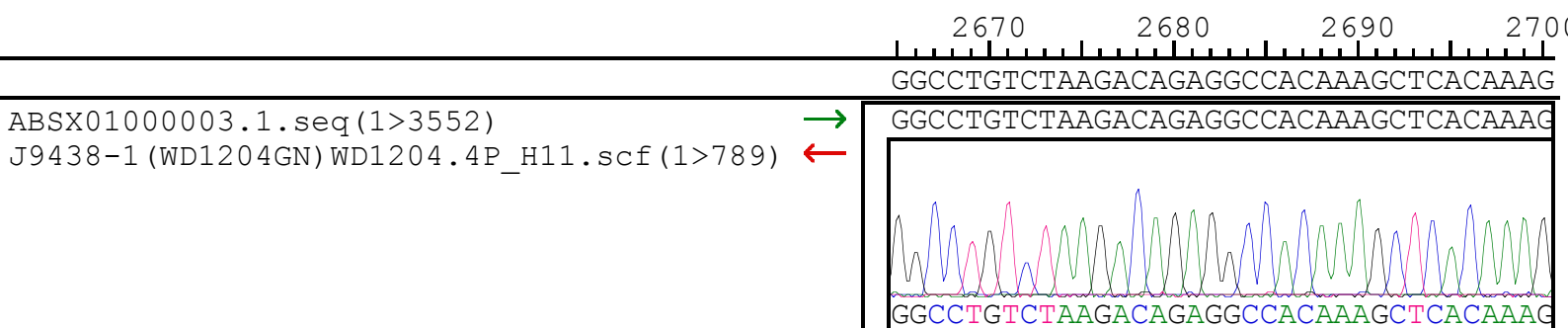

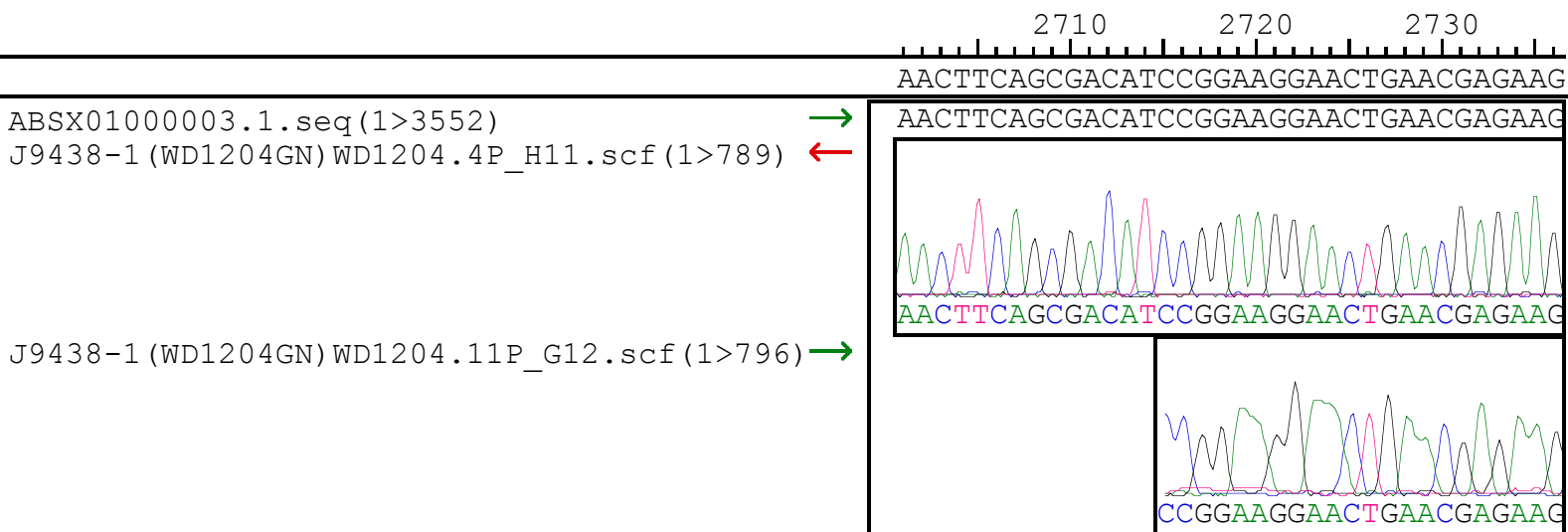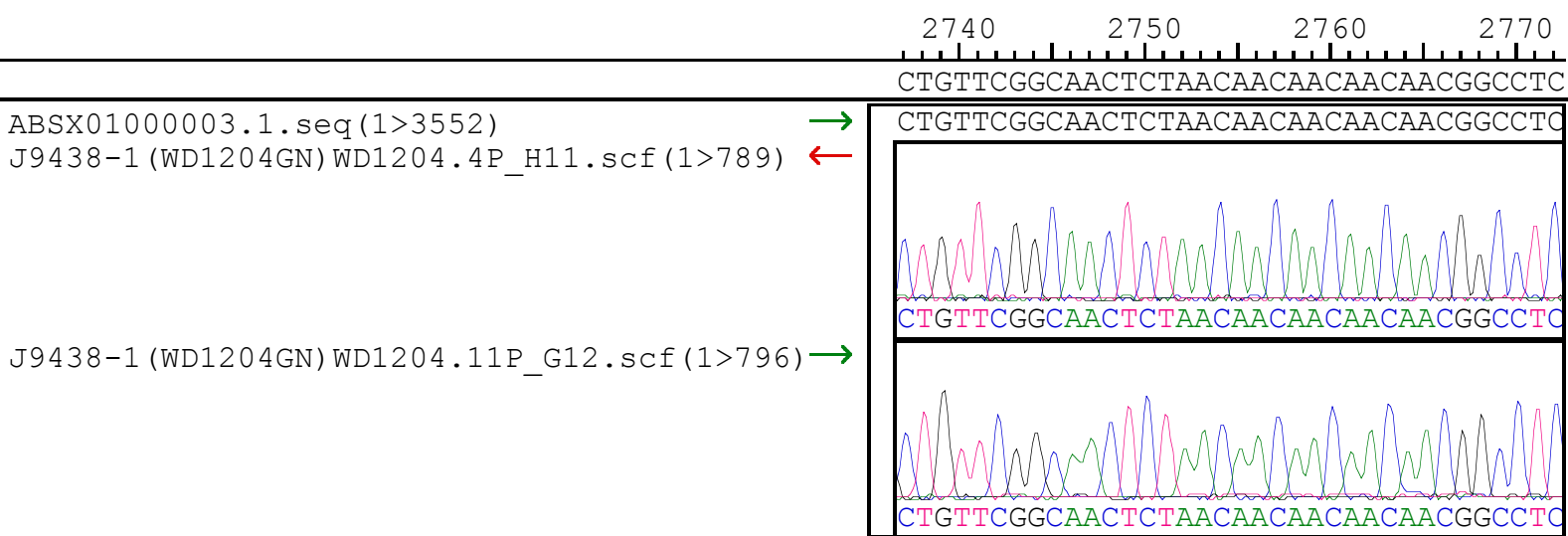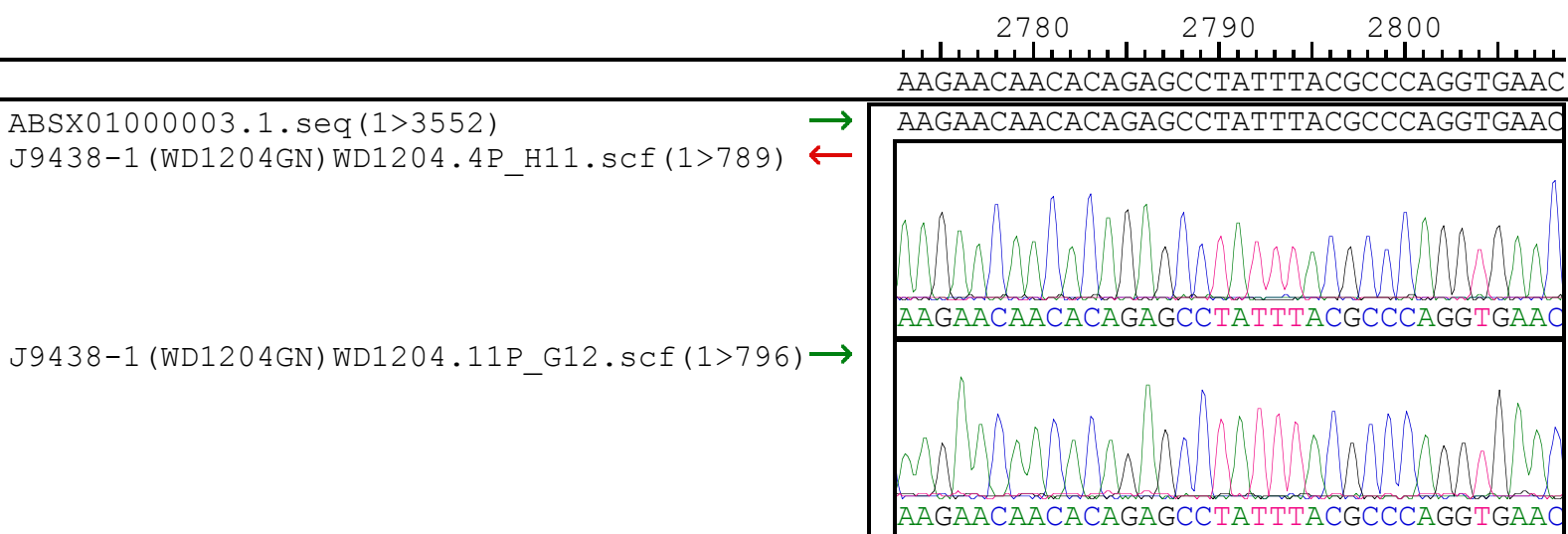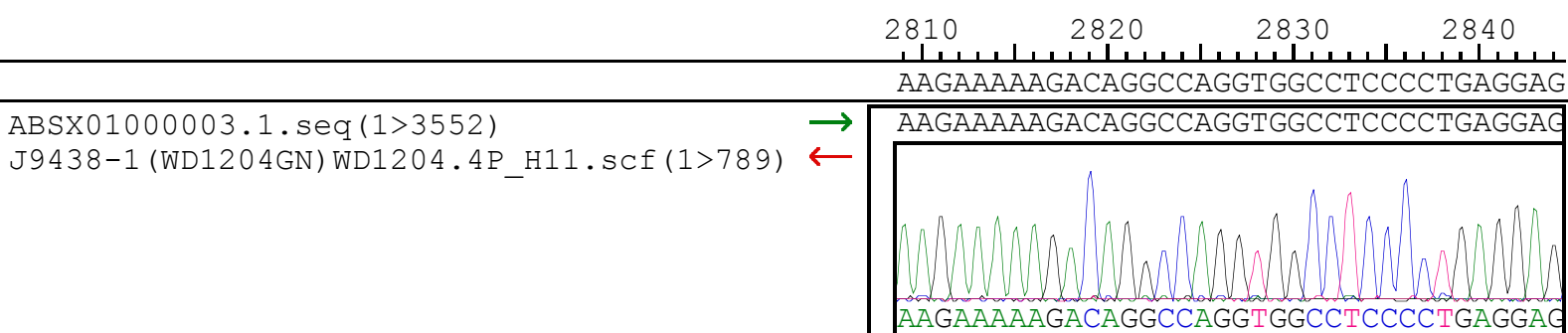

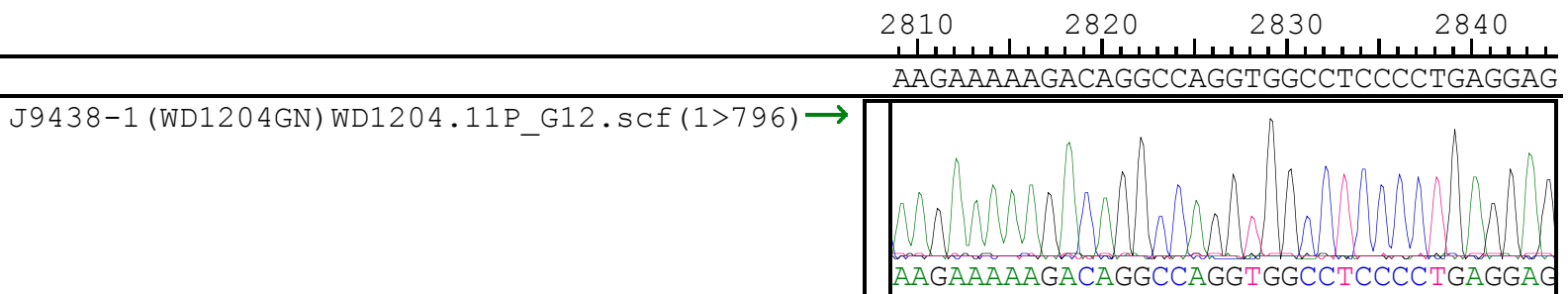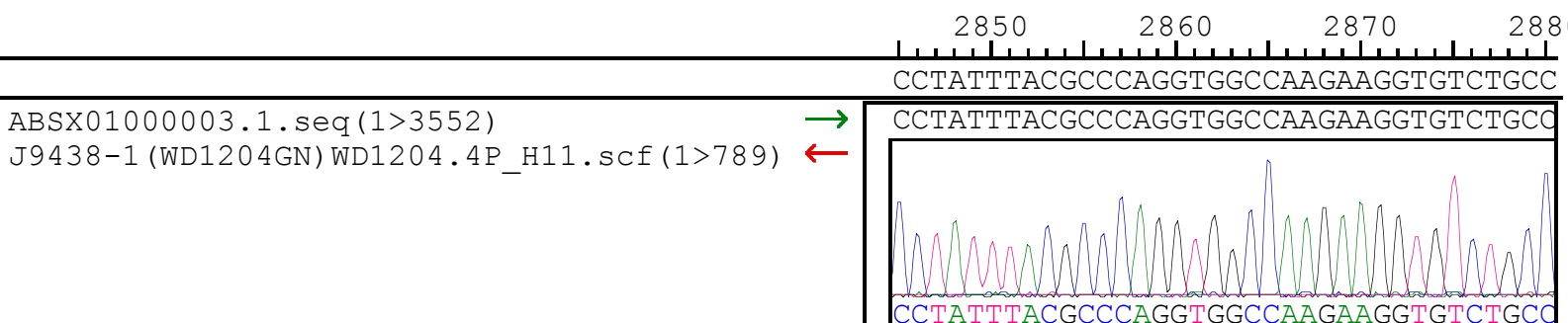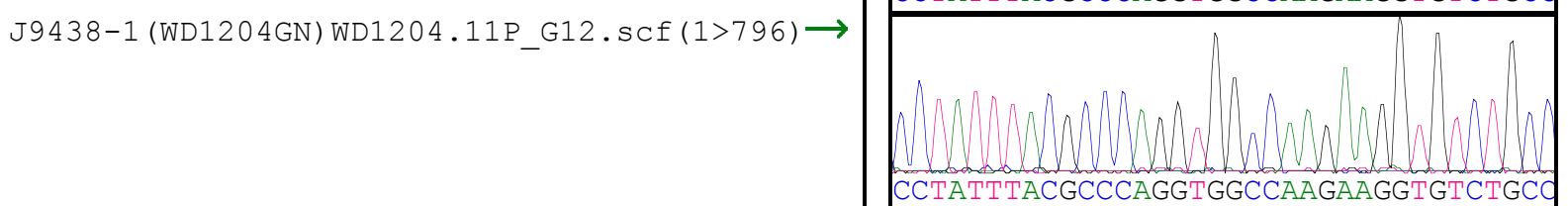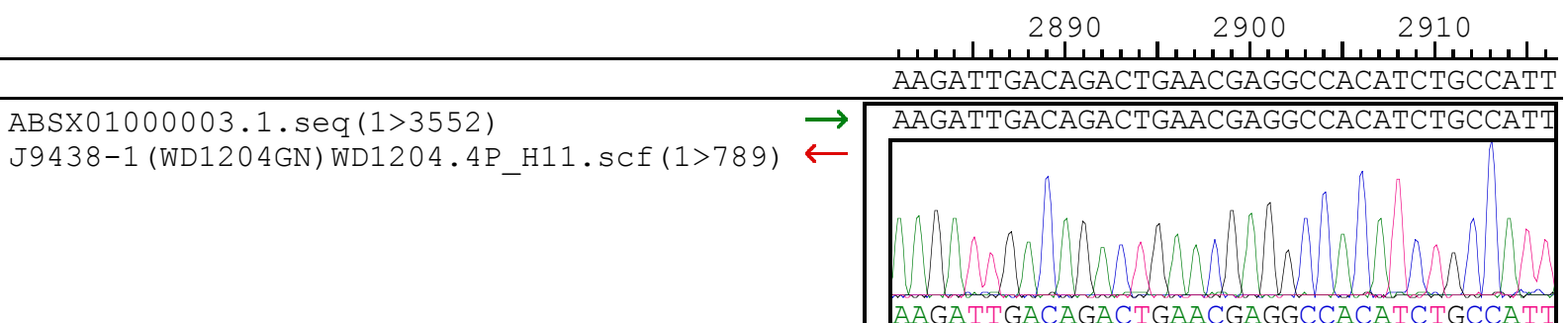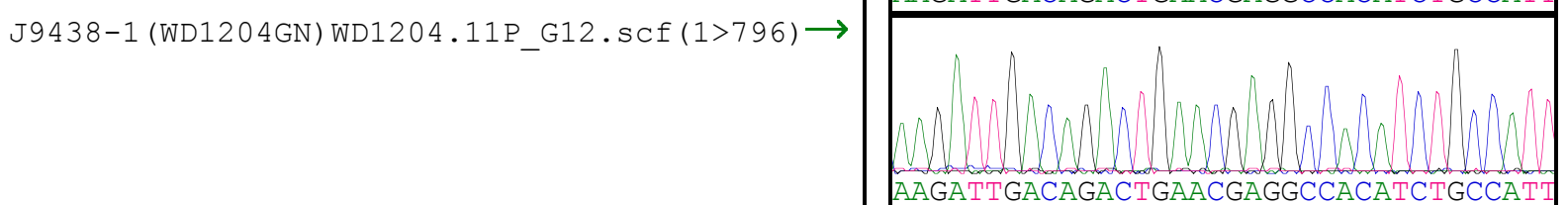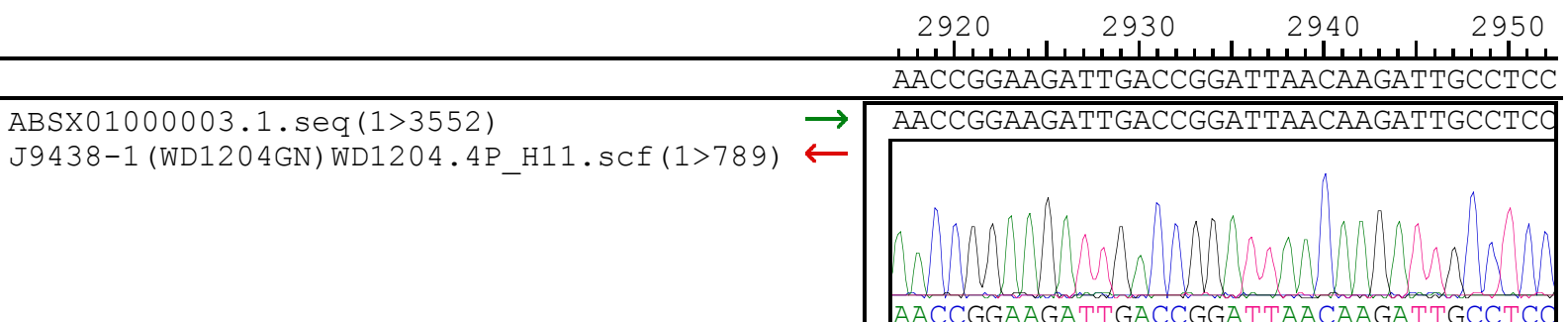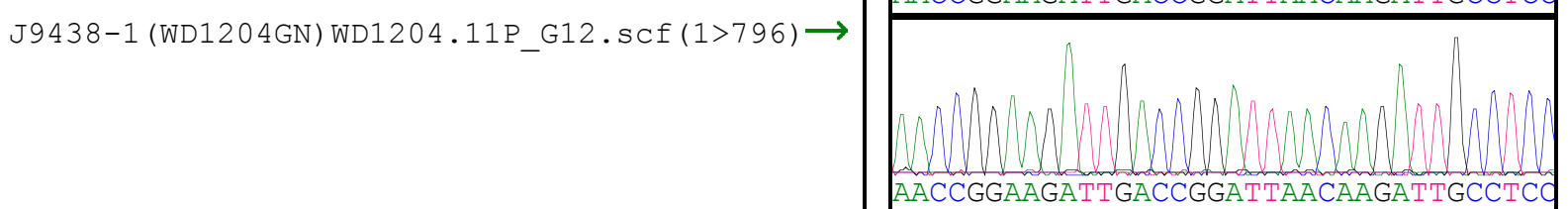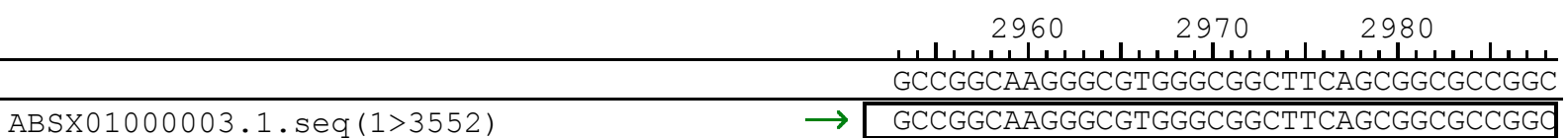

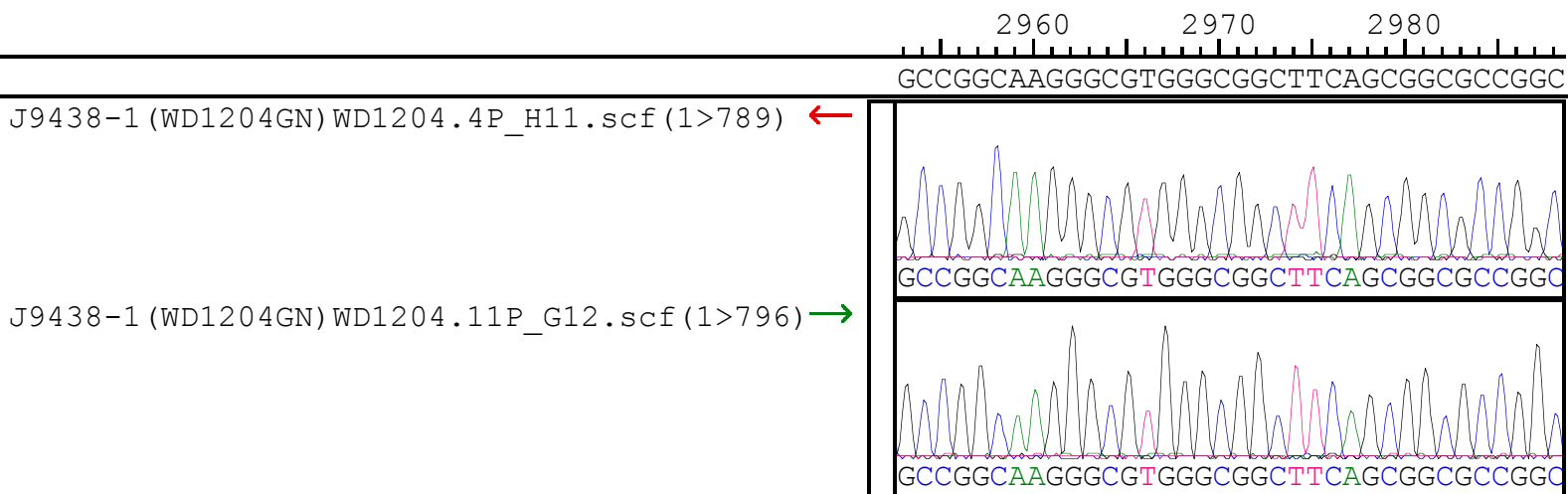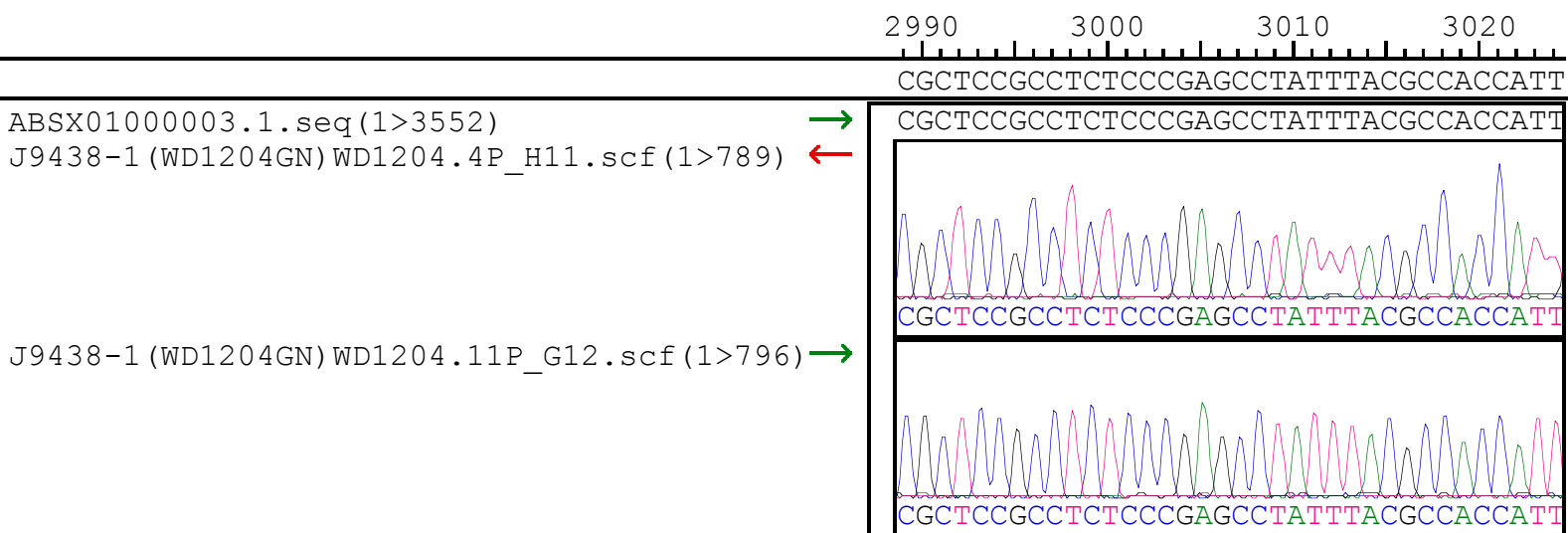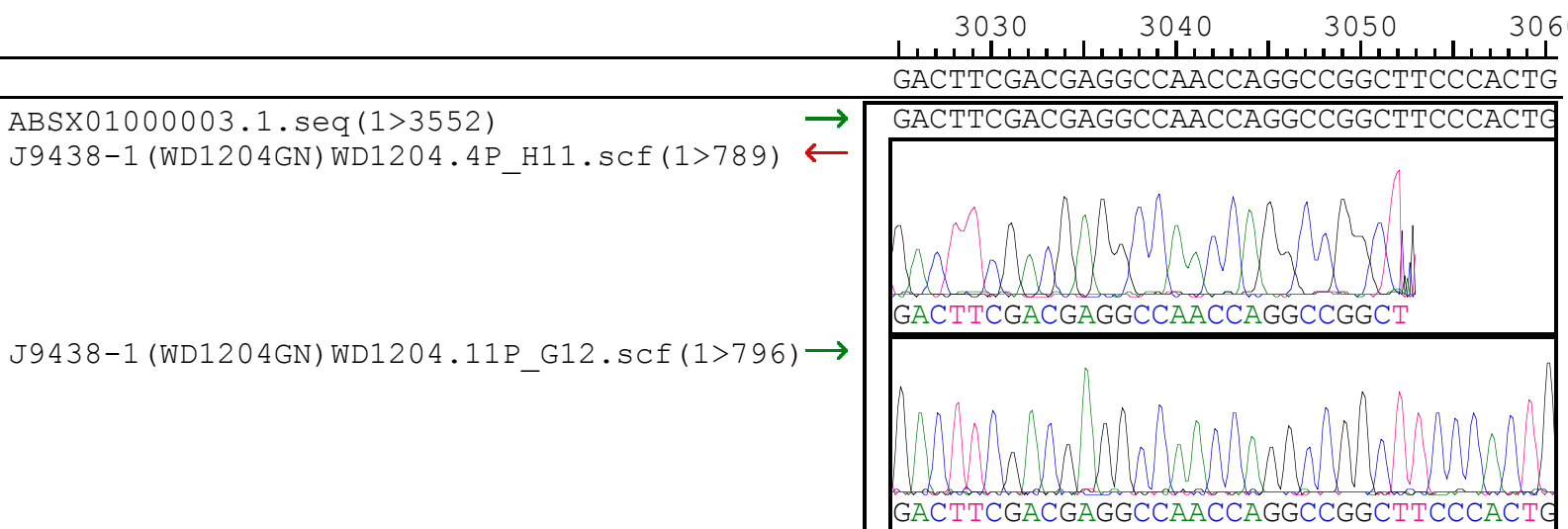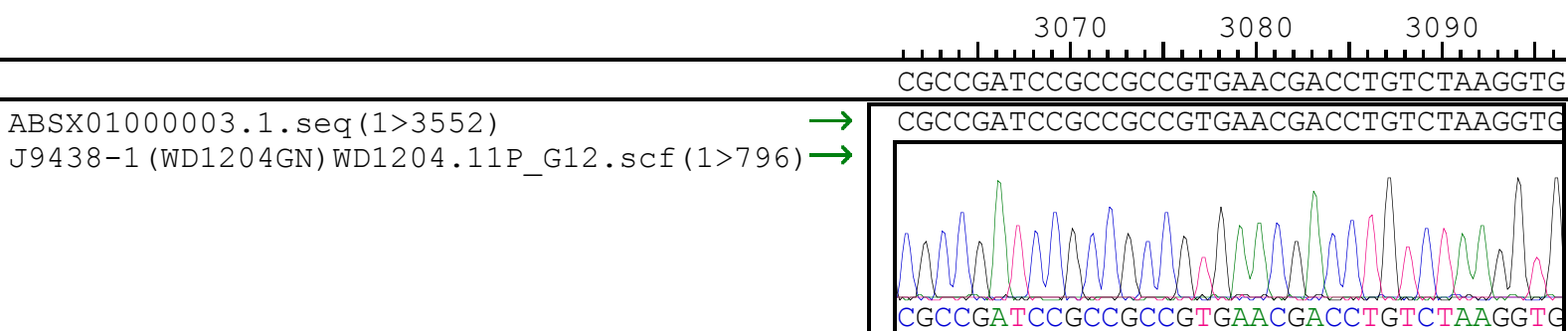

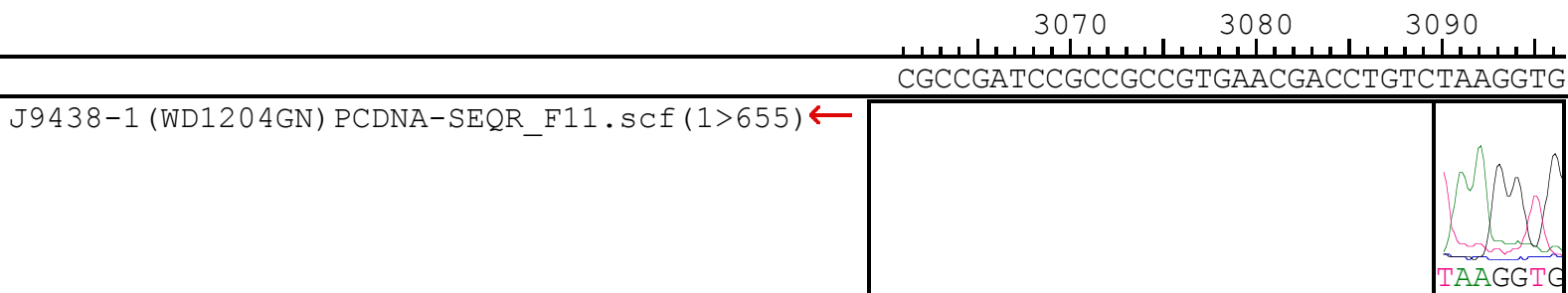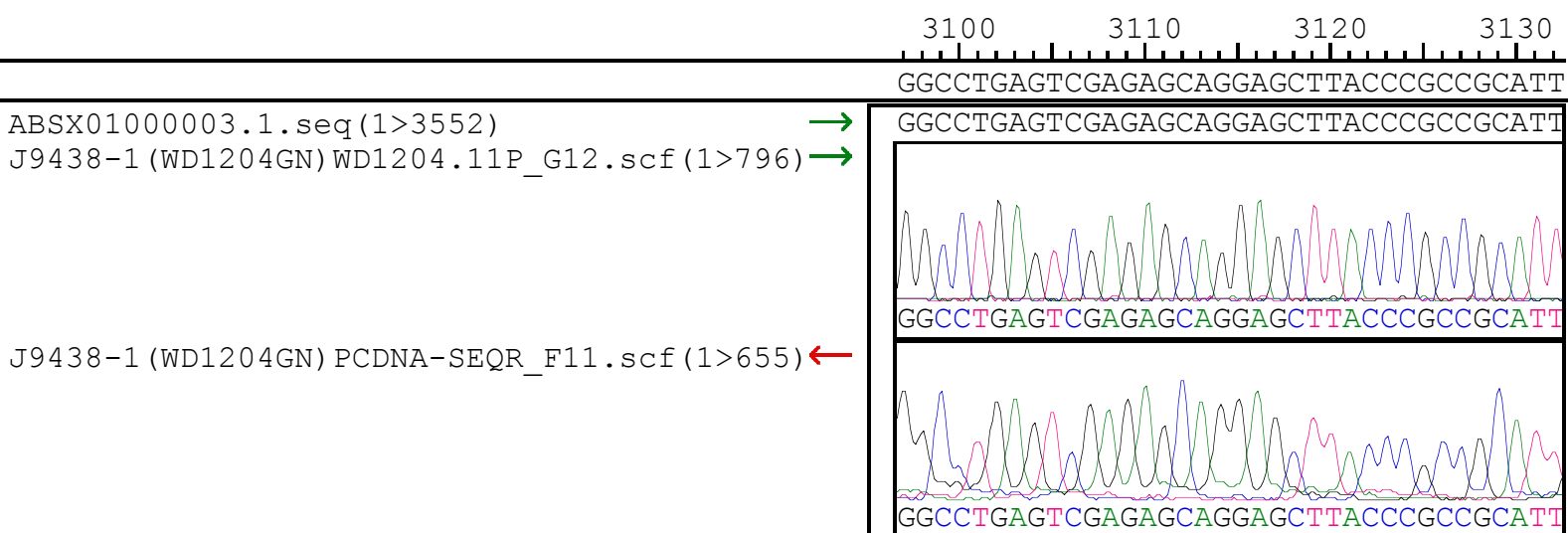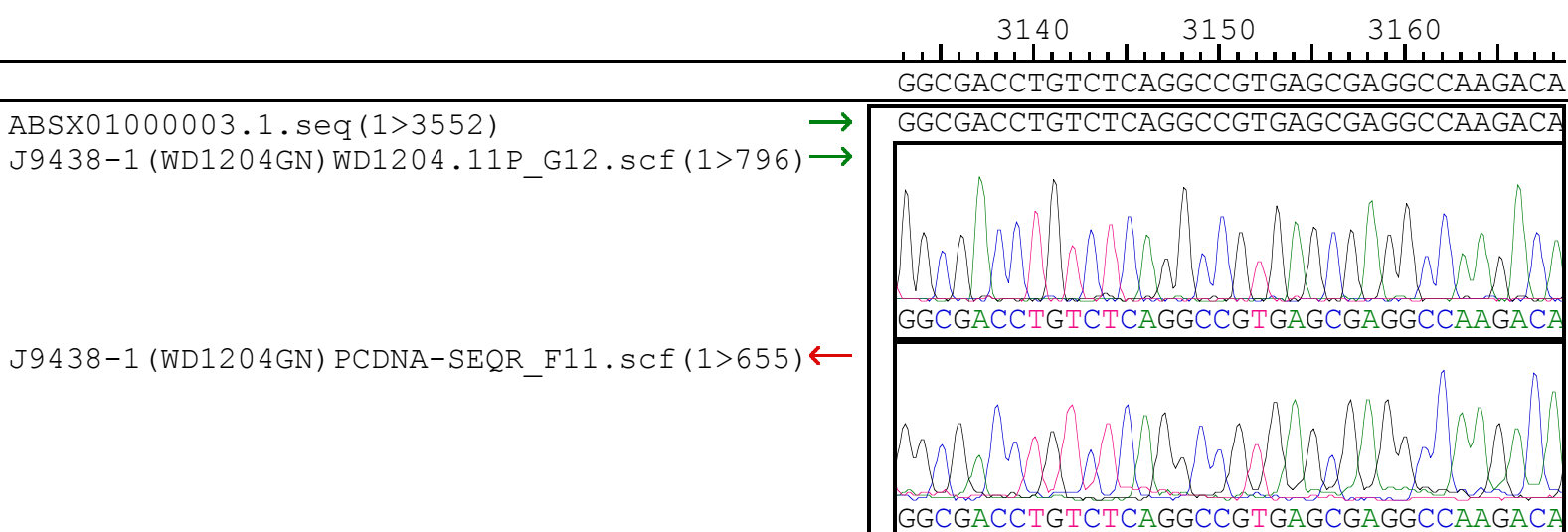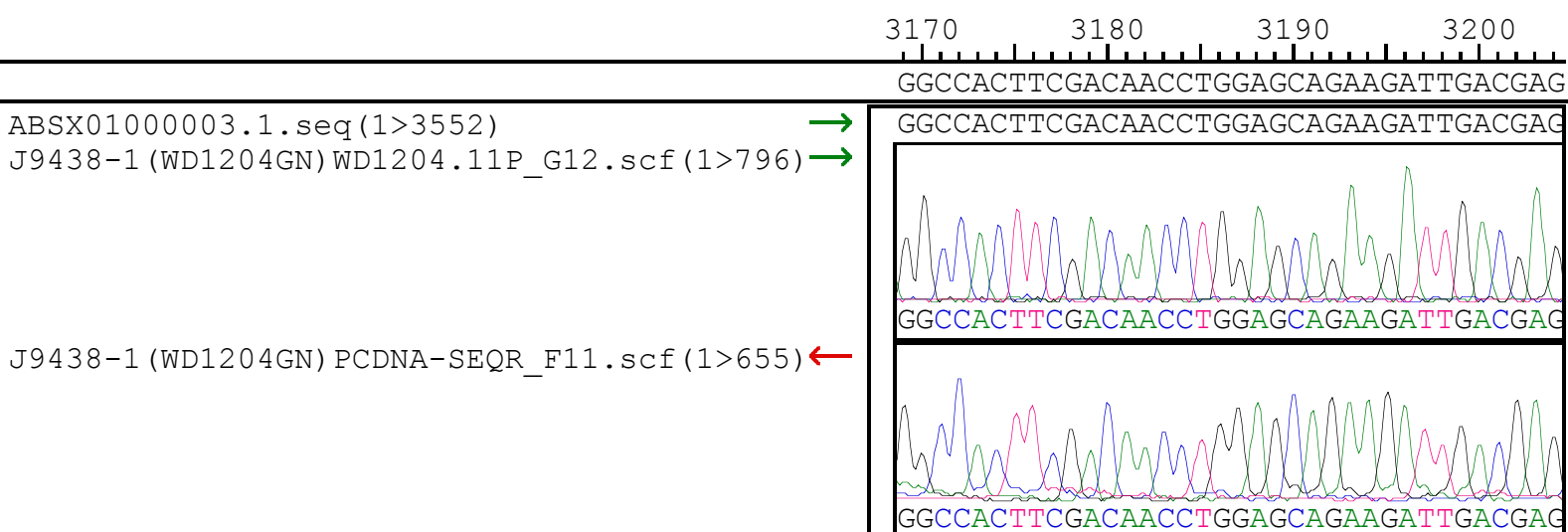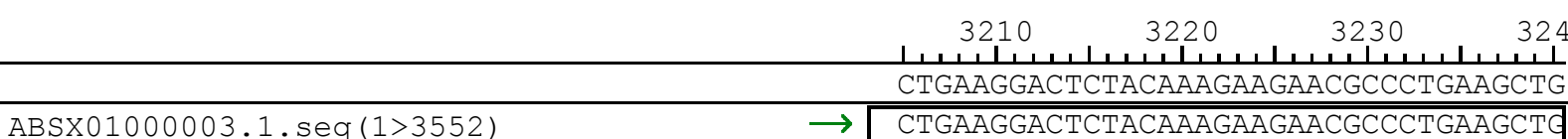

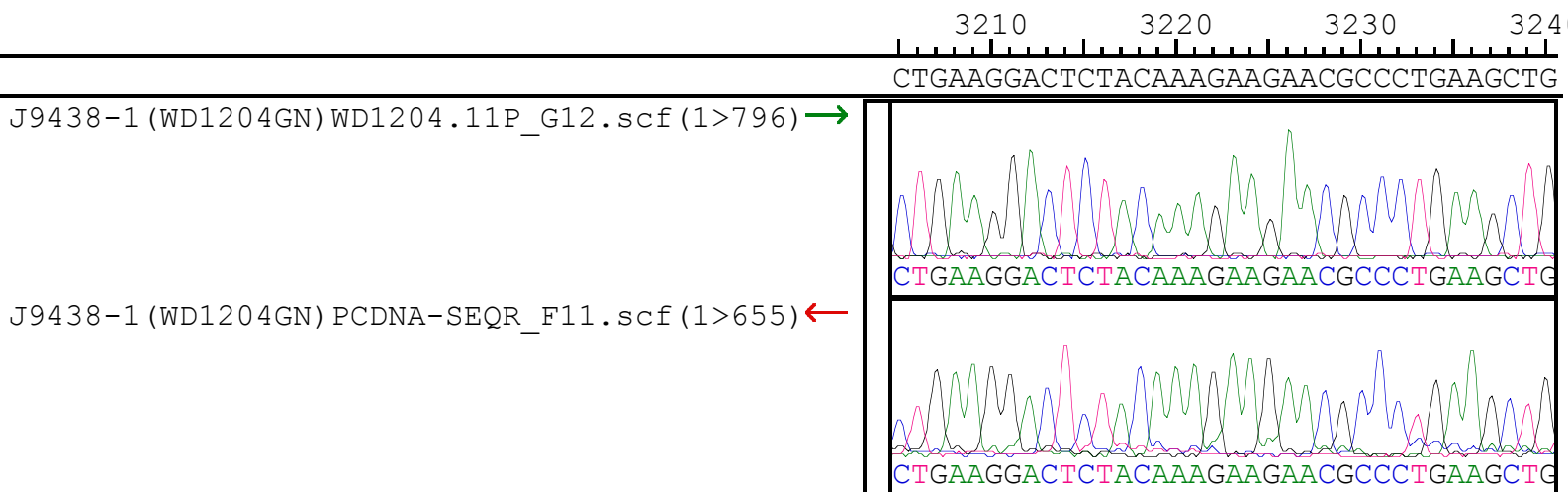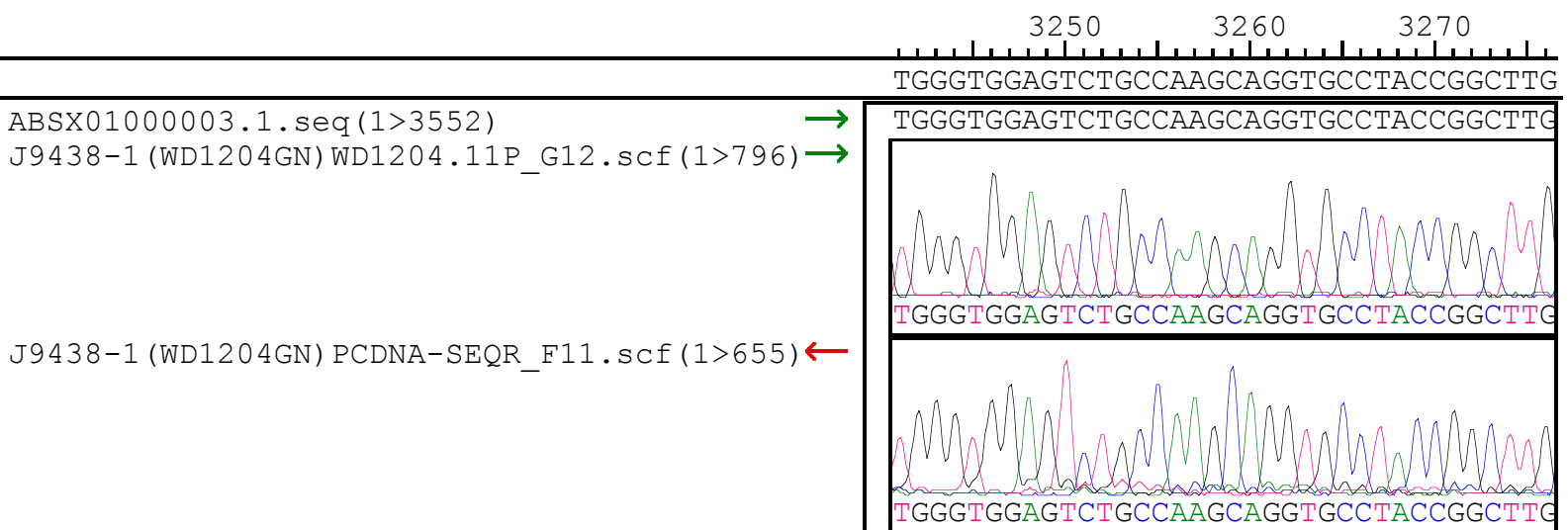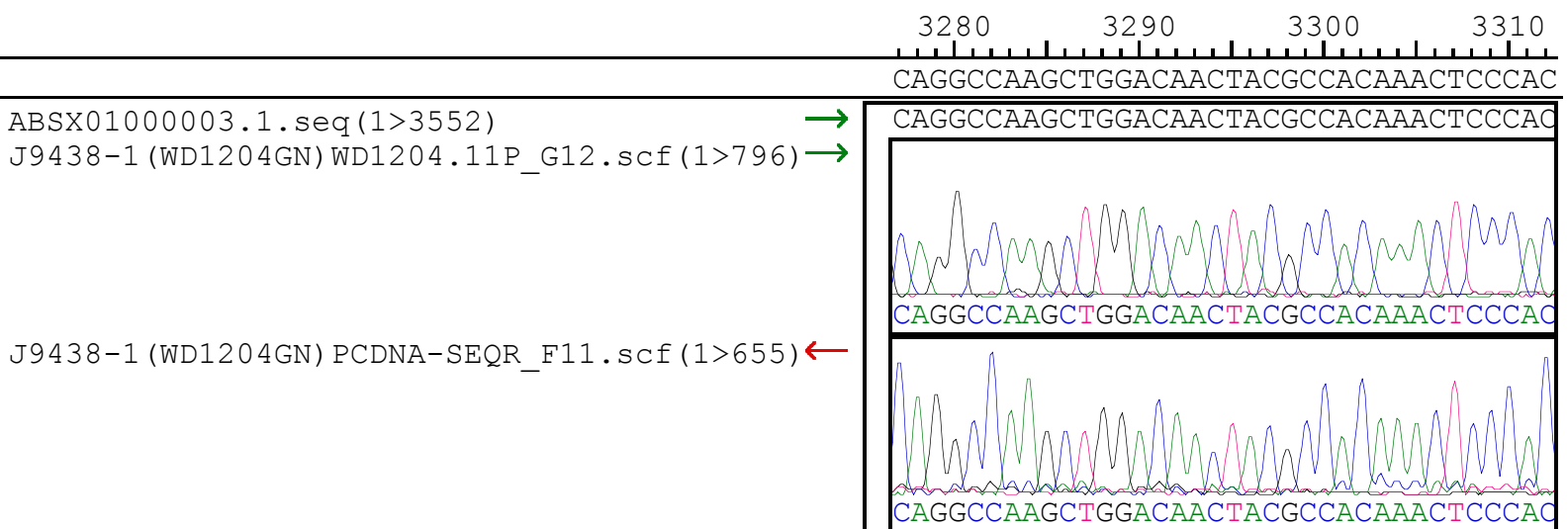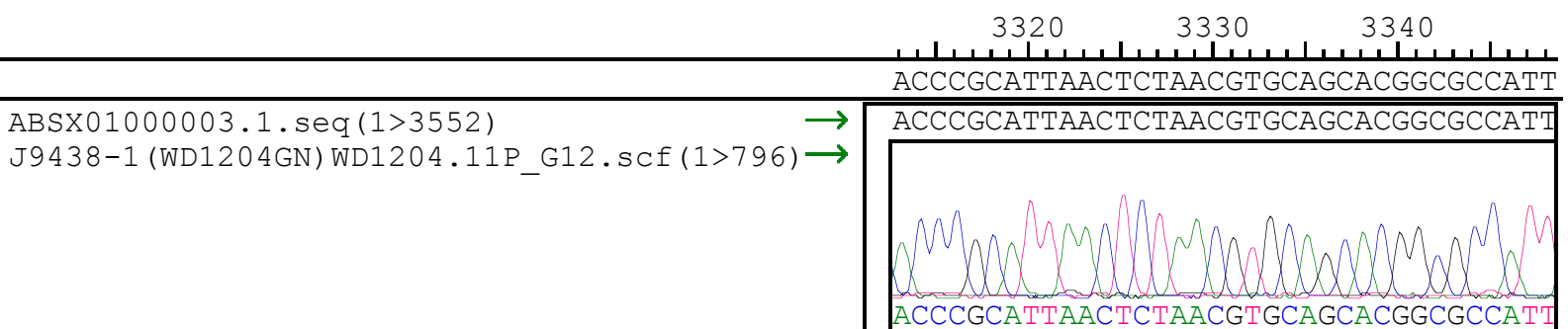

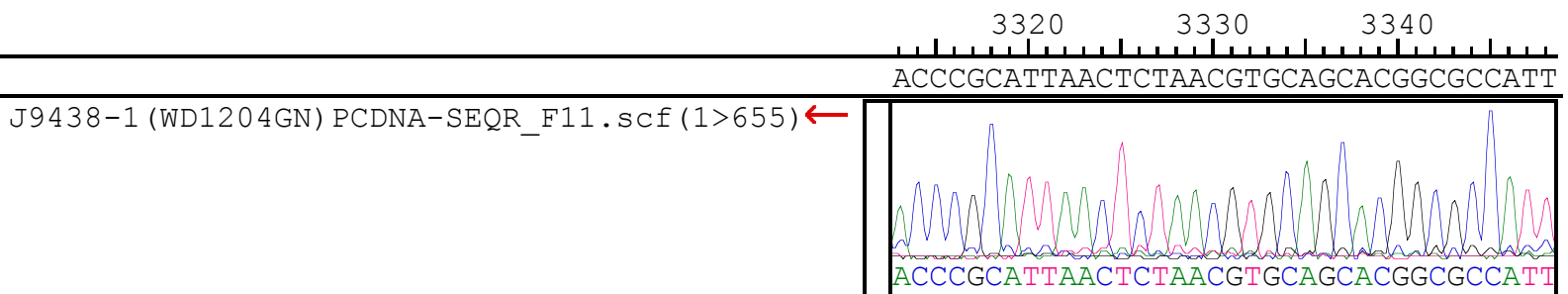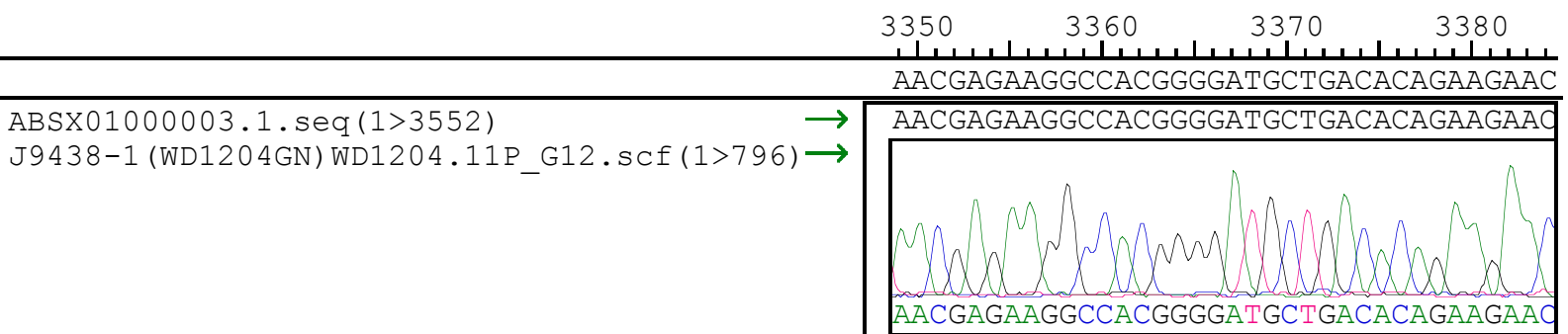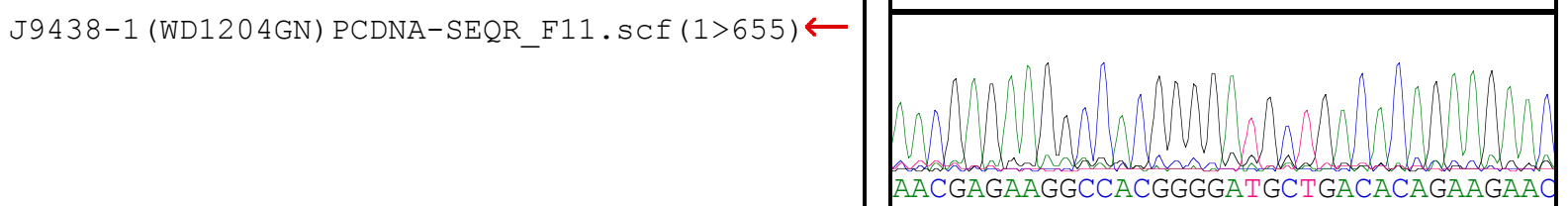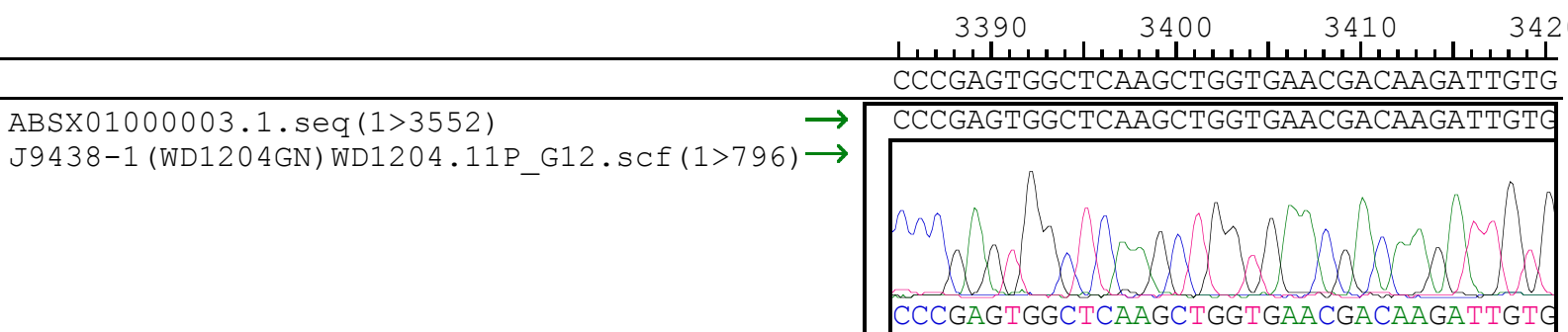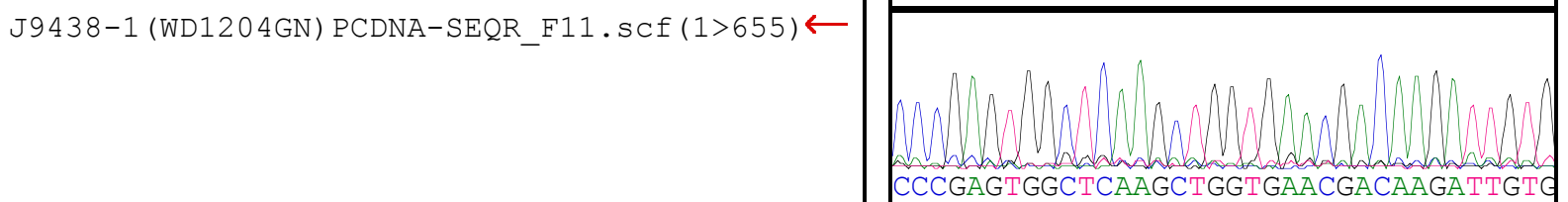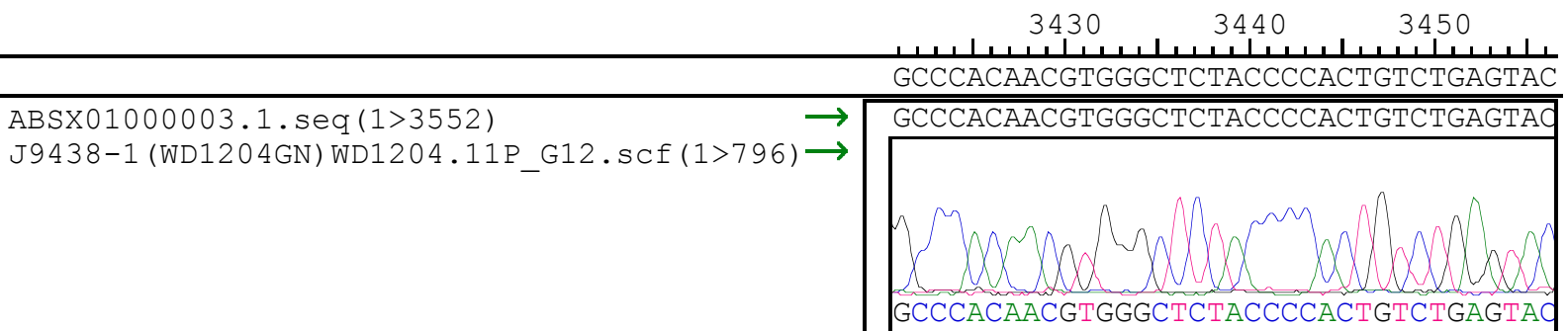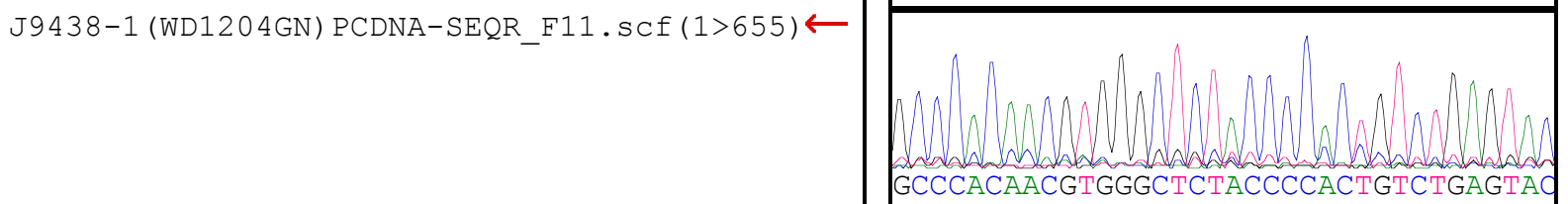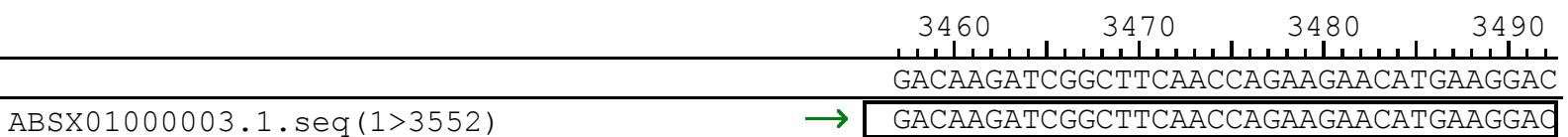

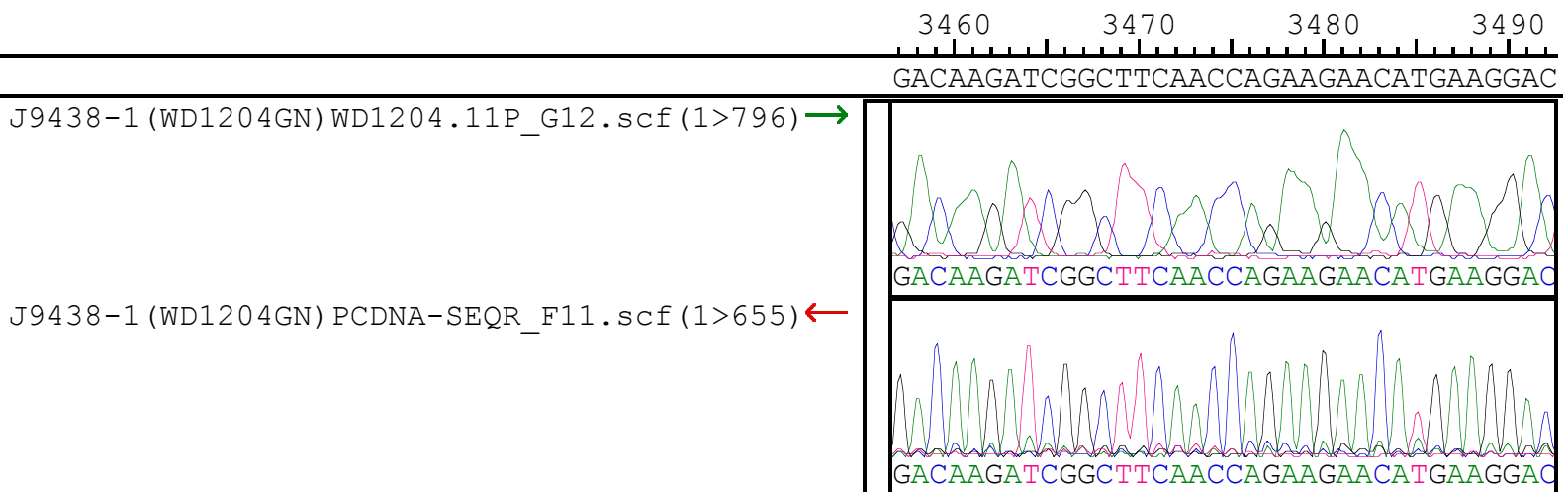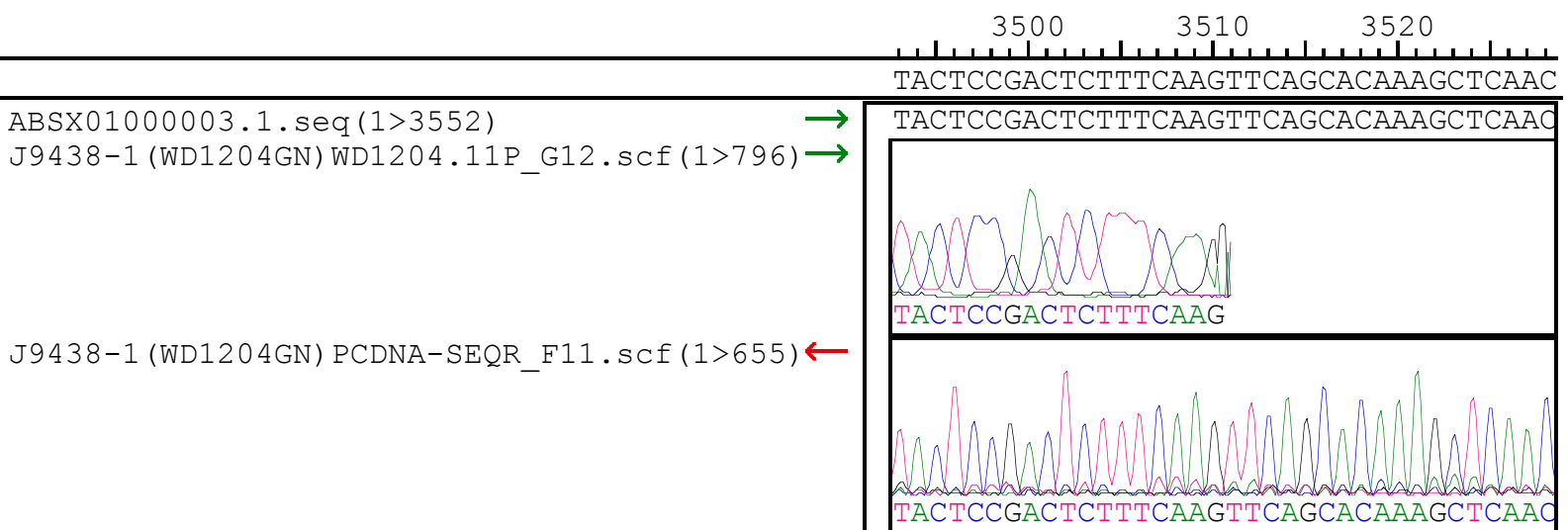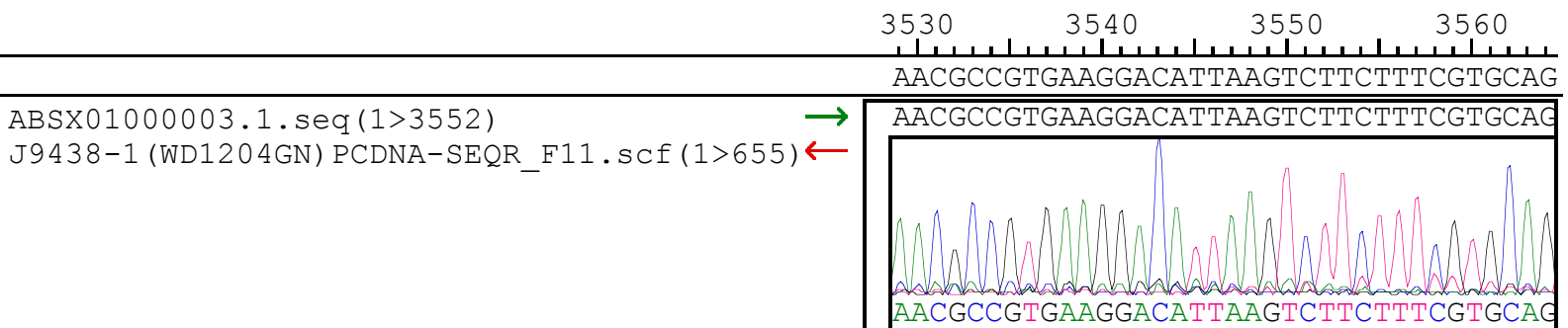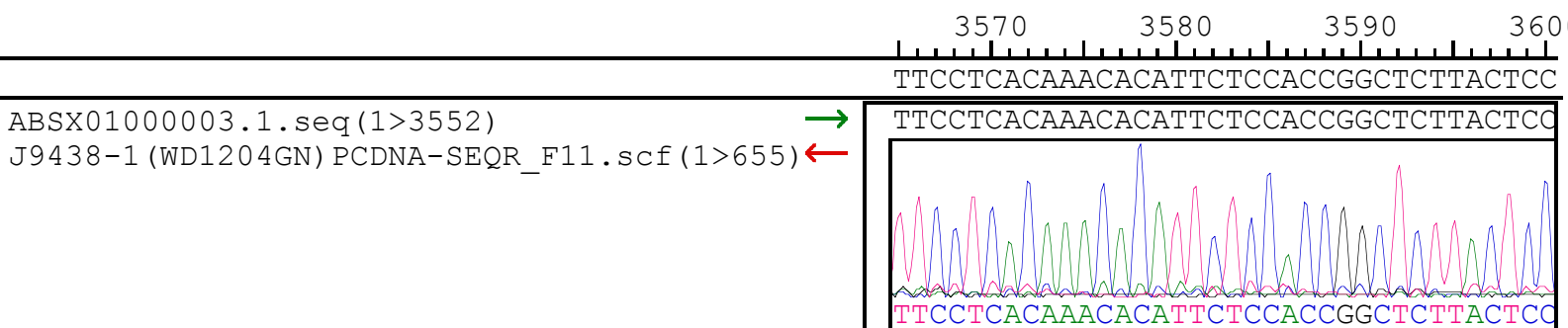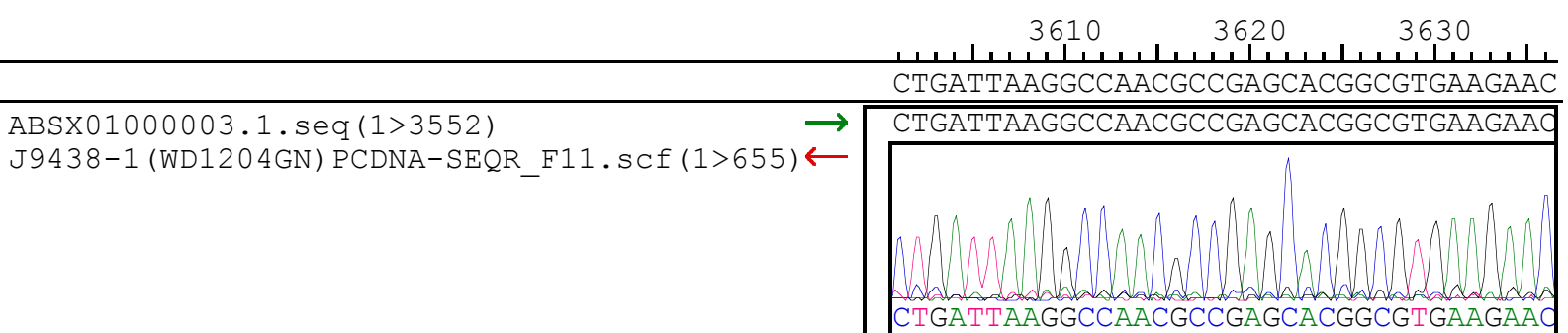

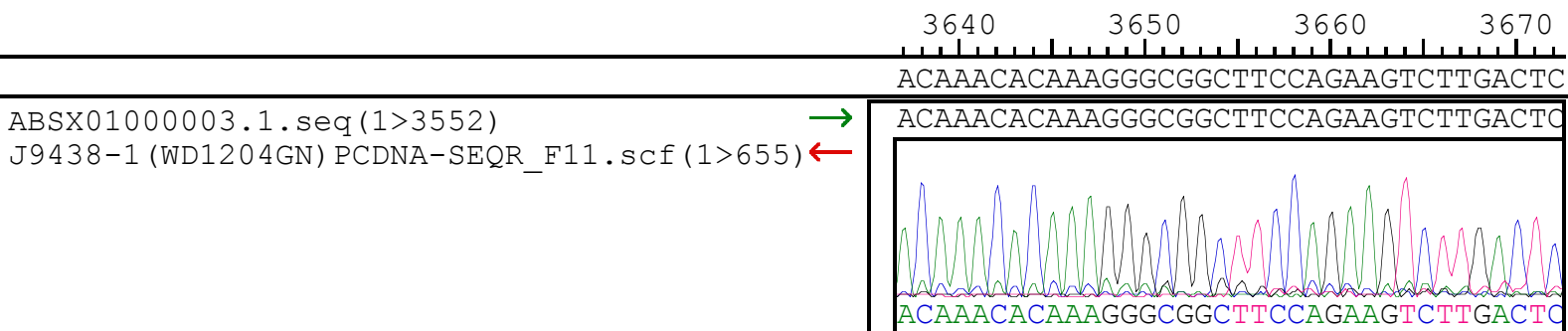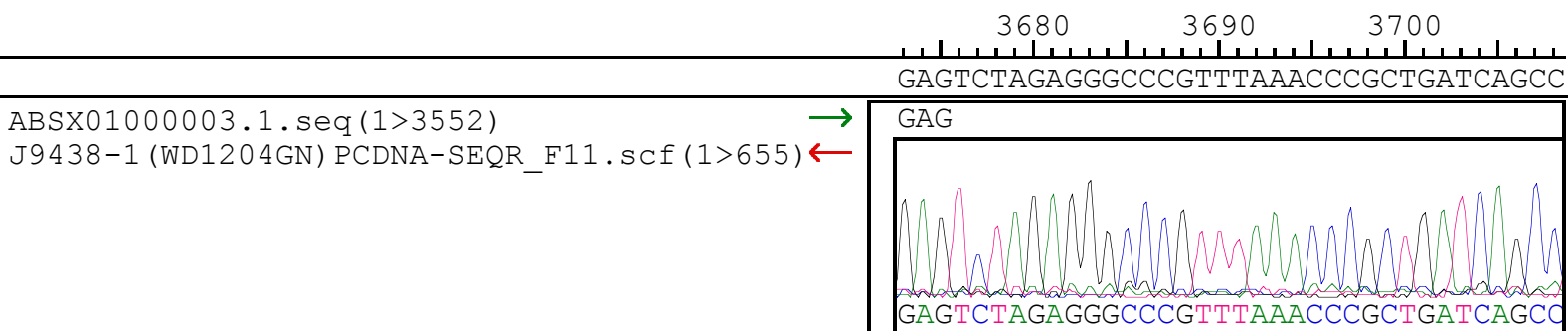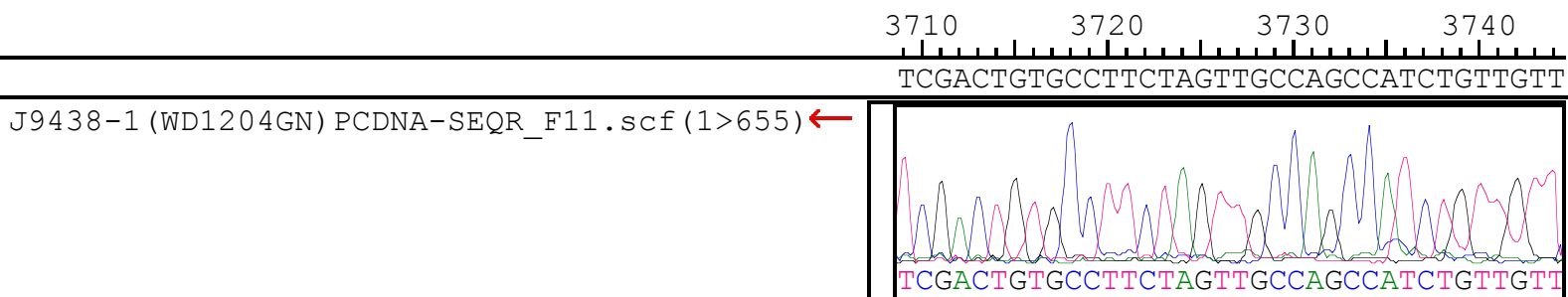

Supplement: Supplementary file 1 [file pathogens-11-00846-s001.zip › File S2.pdf]
